# *Parabacteroides distasonis* ameliorates hepatic fibrosis potentially via modulating intestinal bile acid metabolism and hepatocyte pyroptosis in male mice

**DOI:** 10.1038/s41467-023-37459-z

**Published:** 2023-04-01

**Authors:** Qi Zhao, Man-Yun Dai, Ruo-Yue Huang, Jing-Yi Duan, Ting Zhang, Wei-Min Bao, Jing-Yi Zhang, Shao-Qiang Gui, Shu-Min Xia, Cong-Ting Dai, Ying-Mei Tang, Frank J. Gonzalez, Fei Li

**Affiliations:** 1 Laboratory of Metabolomics and Drug-Induced Liver Injury, Department of Gastroenterology & Hepatology, Frontiers Science Center for Disease-related Molecular Network, West China Hospital, Sichuan University, Chengdu, 610041 China; 2 State Key Laboratory of Phytochemistry and Plant Resources in West China, Kunming Institute of Botany, Chinese Academy of Sciences, Kunming, 650201 China; 3 University of Chinese Academy of Sciences, Beijing, 100049 China; 4 Department of General Surgery, The First People’s Hospital of Yunnan Province, Kunming, 650101 China; 5 Department of Gastroenterology, The second Affiliated Hospital of Kunming Medical University, Kunming, 650101 China; 6 Center for Cancer Research, National Cancer Institute, National Institutes of Health, Bethesda, MD 20892 USA; 7 Sichuan University-Oxford University Huaxi Gastrointestinal Cancer Center, West China Hospital, Sichuan University, Chengdu, 610041 China

**Keywords:** Liver fibrosis, Microbiome

## Abstract

*Parabacteroides distasonis* (*P. distasonis*) plays an important role in human health, including diabetes, colorectal cancer and inflammatory bowel disease. Here, we show that *P. distasonis* is decreased in patients with hepatic fibrosis, and that administration of *P. distasonis* to male mice improves thioacetamide (TAA)- and methionine and choline-deficient (MCD) diet-induced hepatic fibrosis. Administration of *P. distasonis* also leads to increased bile salt hydrolase (BSH) activity, inhibition of intestinal farnesoid X receptor (FXR) signaling and decreased taurochenodeoxycholic acid (TCDCA) levels in liver. TCDCA produces toxicity in mouse primary hepatic cells (HSCs) and induces mitochondrial permeability transition (MPT) and Caspase-11 pyroptosis in mice. The decrease of TCDCA by *P. distasonis* improves activation of HSCs through decreasing MPT-Caspase-11 pyroptosis in hepatocytes. Celastrol, a compound reported to increase *P. distasonis* abundance in mice, promotes the growth of *P. distasonis* with concomitant enhancement of bile acid excretion and improvement of hepatic fibrosis in male mice. These data suggest that supplementation of *P. distasonis* may be a promising means to ameliorate hepatic fibrosis.

## Abstract (web summary)

*Parabacteroides distasonis* (*P. distasonis*), part of the gut microbiome, was reported to play a role in diabetes, colorectal cancer and inflammatory bowel disease. Here the authors report that *P. distasonis* ameliorates liver fibrosis in studies with male mice, potentially via altered bile acid metabolism and hepatocyte pyroptosis.

## Introduction

Hepatic fibrosis is a dynamic process characterized by excessive accumulation of extracellular matrix resulting from chronic liver injury. If left untreated, hepatic fibrosis ultimately leads to cirrhosis, liver failure, or hepatocellular carcinoma. The incidence of hepatic fibrosis is increasing worldwide, and there are no drugs available to protect against or decrease hepatic fibrosis^1^. The gut microbiome, the secondary largest genome of the host, plays an important role in chronic hepatic injury. Various probiotics, such as *Lactobacillus rhamnosus* GG, *Saccharomyces boulardii*, and the selenium-enriched probiotics, *Akkermansia muciniphila*, *Bifidobacterium* and *Bacteroides acidifaciens,* can improve hepatic fibrosis, alcoholic and nonalcoholic fatty liver in mice and rats^2–6^. Notably, it was demonstrated that *Parabacteroides distasonis* (*P. distasonis*) improved obesity, inflammatory bowel disease, colorectal cancer, and testicular dysfunction, and prevented post-calorie restriction weight gain through producing or stimulating host production of various active metabolites such as 3-oxo lithocholic acid (3-oxoLCA), 3-oxo-Δ^4^-LCA, succinate, spermine, agmatine, indolelactic acid, melatonin, uracil and urocanic acid^7–12^. Thus, *P. distasonis* may play an important role in human health.

*Bacteroides* was found to have bile salt hydrolase (BSH) activity that deconjugates primary bile acid derivatives produced in the liver^13^. This transformation of conjugated bile acids to unconjugated derivatives that can then be subjected to further bacterial modifications, is essential for bile acid homeostasis. BSH is negatively correlated with nonalcoholic fatty liver disease (NAFLD) and liver cirrhosis clinically^14^. BSH showed various activity in the host such as lipid-lowering, improving *Clostridioides difficile* infection, and enhancing adhesion ability in the gastrointestinal tract^15–17^. Uncovering the mechanism by which BSH influences hepatic fibrosis would be beneficial for the developing BSH-based therapeutics.

Ileal farnesoid X receptor (FXR) controls bile acid transport across the intestinal epithelial cells into the blood where systemic bile acid metabolites influence inflammation, glucose homeostasis, obesity, insulin resistance, and NAFLD. In the gut, certain bile acid metabolites have antibacterial activity involved in the pathogenesis of inflammatory bowel disease and mucosal injury^18^. Intestinal FXR antagonists such as ursodeoxycholic acid (UDCA), glycine-β-muricholic acid (GβMCA), and glycoursodeoxycholic acid (GUDCA) were suggested in mouse models to be of potential value for the treatment of primary biliary cirrhosis (PBC), NAFLD, obesity and atherosclerosis through regulating the FXR/SMPD3 axis and reducing the biosynthesis of intestine-derived ceramides^18–22^. GUDCA and a betulinic acid derivative, two intestinal FXR antagonists, improved methionine and choline-deficient (MCD) diet-induced nonalcoholic steatohepatitis and hepatic fibrosis^23^. In addition, the probiotic *Lactobacillus rhamnosus* GG, reduced hepatic fibrosis by inhibiting intestinal FXR signaling and decreasing FGF15 expression, and promoting the hepatic synthesis and excretion of bile acids, thus indicating that inhibition of intestinal FXR signaling may be of value as a strategy to alleviate hepatic fibrosis^2^.

The purpose of the current study was to examine the protective role of *P. distasonis* in hepatic fibrosis. *P. distasonis* decreased taurochenodesoxycholic acid (TCDCA) levels by increasing BSH activity and inhibiting ileal FXR. The decrease of TCDCA improved activation of hepatic stellate cells (HSCs) through decreasing the mitochondrial permeability transition (MPT)-Caspase-11 pyroptosis pathway. Celastrol derived from the root of *Tripterygium wilfordii* plant promotes the growth of *P. distasonis* and prevents thioacetamide (TAA)- and MCD diet-induced hepatic fibrosis through increasing BSH activity and inhibiting ileal FXR signaling. Administration of *P. distasonis* leads to increased BSH activity, inhibition of intestinal farnesoid X receptor (FXR) signaling, and decreased TCDCA levels in liver. These findings provide a therapeutic strategy for hepatic fibrosis through supplementation with *P. distasonis*.

## Results

### Levels of *P. distasonis* are decreased in hepatic fibrosis patients

**Fig. 1:**
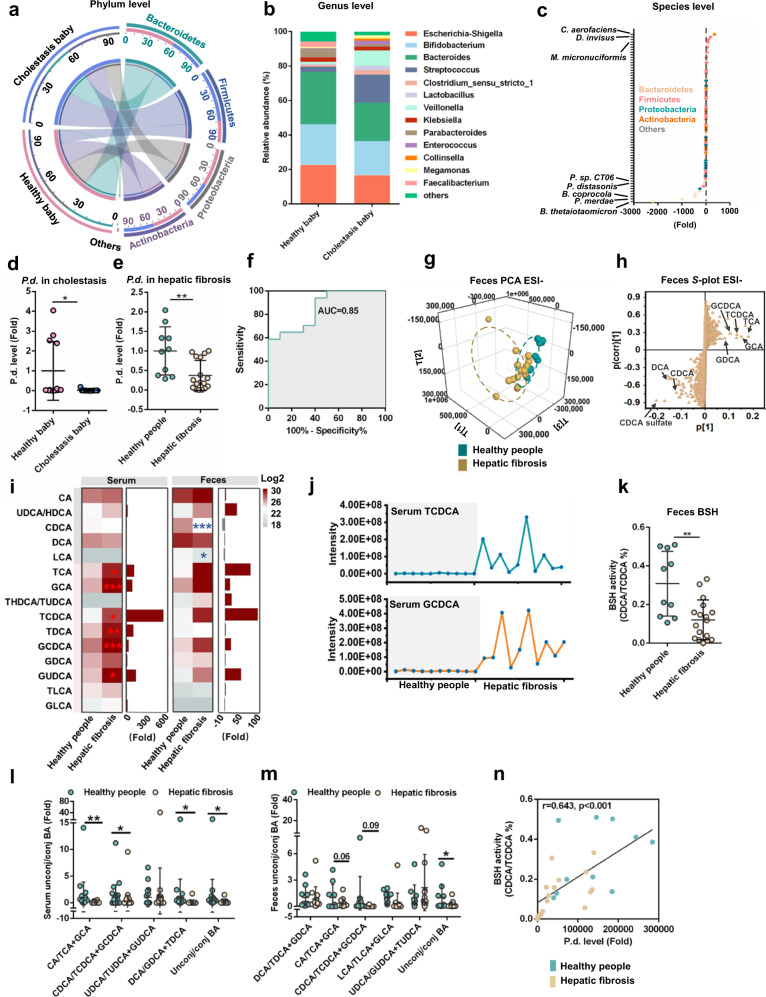
Cholestasis and hepatic fibrosis patients decrease *P. distasonis* levels and BSH activity. **a**–**d** Relative abundance of phylum (**a**), genus (**b**), species (**c**) and *P. distasonis* (**d**) in the feces of healthy babies (*n* = 12) and cholestatic babies (*n* = 13). **c** Different phylum was showed with different color, and negative number showed a decreased fold in cholestatic babies and a positive number showed the increased fold. P.d., *P. distasonis*. **e** *P. distasonis* levels in the feces of healthy subjects (*n* = 10) and hepatic fibrosis patients (*n* = 17). **f** ROC curve analysis of *P. distasonis* levels in healthy people (*n* = 10) and hepatic fibrosis patients (*n* = 17). ROC curves are used to determine diagnostic efficiency using the area under curve (AUC). The range of AUC is between 0.5 and 1. An AUC of 1.0 would indicate perfect prediction and 0.5 would indicate poor prediction. **g** Principal component analysis (PCA) score plot for the feces metabolome of healthy people (*n* = 10) and hepatic fibrosis patients (*n* = 17) detected in ESI-. Each point represented a sample. **h** Bile acids were labeled in the loading plot for feces metabolome in hepatic fibrosis patients. Conjugated bile acids (e.g., taurochenodeoxycholic acid (TCDCA) and glycochendeoxycholic acid (GCDCA)) were increased, and the unconjugated bile acid chenodeoxycholic acid (CDCA) was decreased in feces of hepatic fibrosis patients. **i** Serum and feces bile acid target analysis was shown by heatmap in hepatic fibrosis patients. Red color shows higher bile acid levels and gray color shows lower bile acid levels. Heatmap plots were generated by log2 transformation of data. The fold changes are also shown with bar graphs. For serum samples, healthy people *n* = 25, hepatic fibrosis *n* = 62; for feces samples, healthy people *n* = 10, hepatic fibrosis *n* = 17. **j** Trending plot of TCDCA and GCDCA in serum of healthy people (*n* = 10) and hepatic fibrosis patients (*n* = 10). **k** Bile salt hydrolase (BSH) activity in healthy people (*n* = 10) and hepatic fibrosis patients (*n* = 17). **l** Unconjugated/(glycine conjugated+taurine conjugated) bile acid ratio in the serum of healthy people (*n* = 25) and hepatic fibrosis patients (*n* = 62). BA, bile acid. **m** Unconjugated/(glycine conjugated+taurine conjugated) bile acid ratios in the feces of healthy people (*n* = 10) and hepatic fibrosis patients (*n* = 17). BA, bile acid. **n** Correlation analysis between *P. distasonis* levels and BSH activity in healthy people (*n* = 10) and hepatic fibrosis patients (*n* = 17). Correlation analysis was performed using Spearman’s rank tests. Data are presented as the mean ± SD. *P < 0.05, **P < 0.01, ***P < 0.001. Source data are provided as a Source Data file.

Data mining from the BIG Data Center (CRA001920) found that clinical cholestasis was associated with decreased *Bacteroidetes* (phylum) and *Parabacteroides* (genus) levels (Fig. 1a, b)^24^. *P. distasonis* was decreased in clinical cholestasis patients (Fig. 1c, d), clinical hepatic fibrosis patients (Fig. 1e), and TAA-induced hepatic fibrosis in mice (Supplementary Fig. 1a). Receiver operating characteristic (ROC) analysis revealed that *P. distasonis* had high discriminatory power to clinically identify hepatic fibrosis (Fig. 1f). *P. distasonis* was negatively correlated with the hepatic fibrosis indexes including alkaline phosphatase (ALP), total bile acid (TBA), *Col1a1*, tissue inhibitor of metalloproteinase-1 (*Timp1*), smooth muscle actin 2 (*Acta2*), transforming growth factor β (*Tgfb*) mRNA expression and severity degree (Child and MELD scores) in clinical samples and in mice (Supplementary Fig. 1b, c, Supplementary Fig. 2d). These results showed that *P. distasonis* may play an important role in the development of hepatic fibrosis.

Metabolomics analysis revealed that conjugated bile acids in serum and feces (e.g., TCDCA and glycochendeoxycholic acid (GCDCA)) were increased and unconjugated bile acids in feces (e.g., chenodeoxycholic acid (CDCA)) were decreased in hepatic fibrosis patients, thus indicating lower BSH activity (Fig. 1h, i, Supplementary Fig. 1d). Feces BSH levels were decreased, and unconjugated bile acid/conjugated bile acid ratios in serum and feces were decreased in clinical hepatic fibrosis patients (Fig. 1k–m). *Lactobacillus* (Genus), *Bifidobacterium* (Genus), *Clostridium* (Genus), *Bacteroides* (Genus), *Parabacteroides* (Genus) potential express BSH^13^. Among all these bacteria, only *P. distasonis* levels were decreased in clinical cholestasis patients and in the TAA-induced hepatic fibrosis mouse model, implying that *P. distasonis* may have BSH activity (Supplementary Fig. 2e, f). *P. distasonis* levels were positively correlated with BSH activity in clinical feces samples (Fig. 1n).

### TAA-induced hepatic fibrosis in mice was improved by *P. distasonis*

In order to determine the role of gut microbiota on hepatic fibrosis, bacteria in the intestinal tract were depleted by administration of an antibiotic cocktail (ampicillin, 0.25 mg/mL; neomycin, 0.25 mg/mL; metronidazole, 0.25 mg/mL; vancomycin, 0.125 mg/mL) and then the gut microbiota was reconstructed by fecal microbial transplantation (FMT) from healthy mice (Supplementary Fig. 3). Loss of gut microbiota increased TAA-induced hepatic fibrosis as revealed by histology, increased bile acids in serum and liver, decreased body weights, increased aspartate aminotransferase (AST) and alanine aminotransferase (ALT) activities, and increased expression of proinflammatory factor and hepatic fibrosis gene mRNAs (interleukin-1 (*Il1*), *Il6*, tumor necrosis factor α (*Tnfa*) and *Timp1*) (Supplementary Fig. 3a–f). These pathological features including histology, AST activity, proinflammatory factor, and hepatic fibrosis gene expression (*Il1*, *Il6*, and *Tgfb*) could be improved following FMT from healthy mice to antibiotic cocktail-treated diseased mice (Supplementary Fig. 3g–k). These results show that gut microbiota play an important role in TAA-induced hepatic fibrosis.

**Fig. 2:**
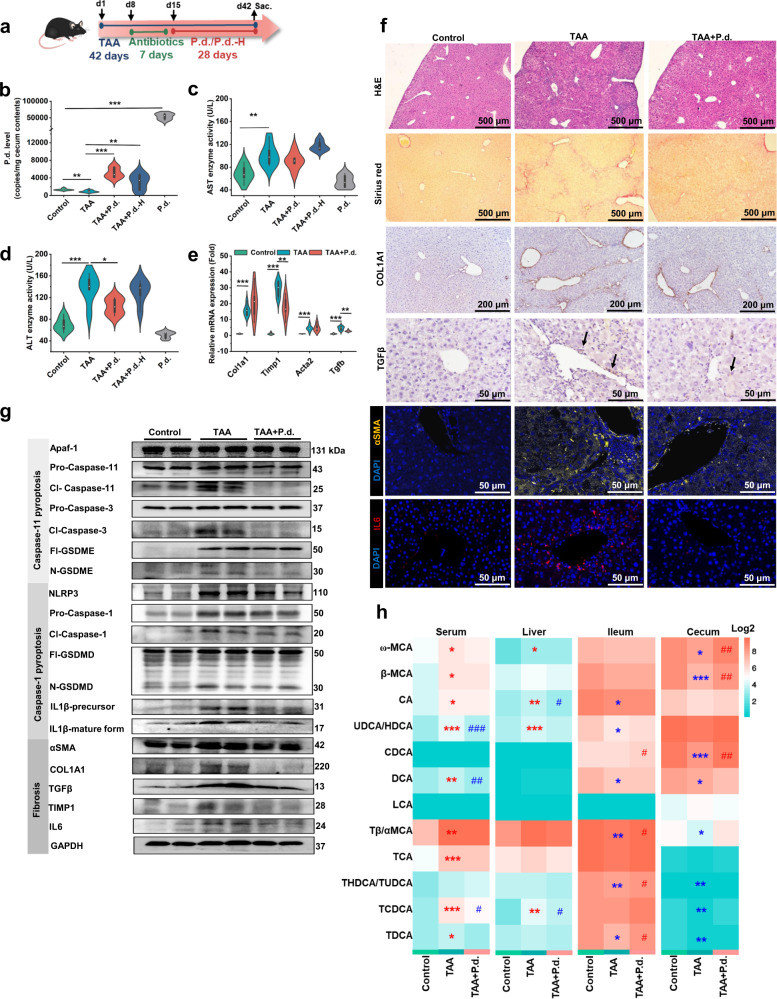
*P. distasonis* improves thioacetamde (TAA)-induced hepatic fibrosis in mice. **a** Experimental scheme: mice were treated with 200 mg/kg TAA for 6 weeks. After TAA treatment for 1 week, the mice were treated with antibiotics (ampicillin, neomycin, metronidazole, and vancomycin) for 1 week. After antibiotics treatment, 2 × 10^8^ CFU *P. distasonis* (P.d.) and heat-killed *P. distasonis* (P.d.-H) were given by oral transplantation once a day for 4 weeks. *n* = 6 per group. **b** Copies of *P. distasonis* in mouse cecum content. **c**, **d** Serum AST (**c**) and ALT (**d**) enzyme activities. **e** Hepatic fibrosis gene expression in mouse liver. *P < 0.05, **P < 0.01, ***P < 0.001. **f** Hepatic H&E, Sirius red, immunohistochemistry (COL1A1 and TGFβ) and immunofluorescence (αSMA and IL6) stainings. H&E staining showed inflammatory infiltration, Sirius red showed increased hepatic fibrosis, immunohistochemistry, and immunofluorescence showed increased COL1A1, TGFβ, αSMA, and IL6 protein expressions in TAA-induced hepatic fibrosis. **g** Caspase-11 pyroptosis pathway (Apaf-1-Caspase-11-Caspase-3-GSDME), Caspase-1 pyroptosis pathway (NLRP3-Caspase-1-GSDMD/IL1β) and hepatic fibrosis (αSMA, COL1A1, TGFβ and TIMP1) protein expression in mouse liver. Cl-Caspase-1/3/11, N-GSDME/GSDMD, and IL1β-mature form were the active forms of the proteins. **h** *P. distasonis* improved bile acid levels in the enterohepatic circulation (serum, liver, ileum, and cecum content) in the Control, TAA, and TAA + P.d. groups. Red color shows higher bile acid levels and green color shows lower bile acid levels. Heatmap plots were generated by log2 transformation of data. *P < 0.05, **P < 0.01, ***P < 0.001 verse Control group; ^#^*P* < 0.05, ^##^*P* < 0.01, ^###^*P* < 0.001 verse TAA group. For the violin plot (**b**–**e**), boxplots represent median with the interquartile range, whiskers indicate adjacent values, violin represents kernel density estimation. Source data are provided as a Source Data file.

To evaluate the effect of *P. distasonis* in TAA-induced hepatic fibrosis, *P. distasonis* transplantation was carried out (Fig. 2a). *P. distasonis* (species) and *Bacteroidetes* (phylum) in cecum lumen were significantly increased after transplantation (Fig. 2b, Supplementary Fig. 4c). This resulted in improved histology and decreased ALT, proinflammatory factor, and hepatic fibrosis gene mRNAs (*Il1*, *Il6*, *Timp1* and *Tgfb*) and proteins expression (IL1β, IL6, α-smooth muscle actin (αSMA), COL1A1, TGFβ and TIMP1) revealing that *P. distasonis* could improve TAA-induced hepatic fibrosis (Fig. 2c–g, Supplementary Fig. 4d). Non-targeted serum metabolomics further revealed that levels of 31 metabolites were altered by *P. distasonis*, including 3 bile acids, 7 acylcarnitines, and 5 lipids (Supplementary Fig. 4e, f). Targeted analysis of bile acids in serum, liver, duodenum, jejunum, ileum, and cecum content was then conducted. *P. distasonis* decreased bile acids in serum and liver and increased bile acids in ileum and cecum content, indicating that *P. distasonis* promoted the excretion of bile acids in enterohepatic circulation (Fig. 2h and Supplementary Fig. 4g). Both bile acids and acylcarnitines were positively correlated with liver injury indexes (e.g., ALT, *Timp1* and *Tgfb* mRNAs) (Supplementary Fig. 5b). *P. distasonis* decreased bile acids in serum and liver in healthy mice (Supplementary Fig. 4g) but did not influence acylcarnitine levels (Supplementary Fig. 5c, d). Hepatic bile acid synthesis and transporter gene mRNAs (*Fxr*, organic anion transporting polypeptide 4 (*Oatp4*), and bile salt export pump (*Bsep*)) that were decreased by TAA, were reversed by *P. distasonis* treatment (Supplementary Fig. 4h). These results showed that *P. distasonis* promoted the excretion of bile acids and improved hepatic fibrosis.

### *P. distasonis* increases BSH activity and inhibits ileal FXR signaling in TAA-induced hepatic fibrosis

**Fig. 3:**
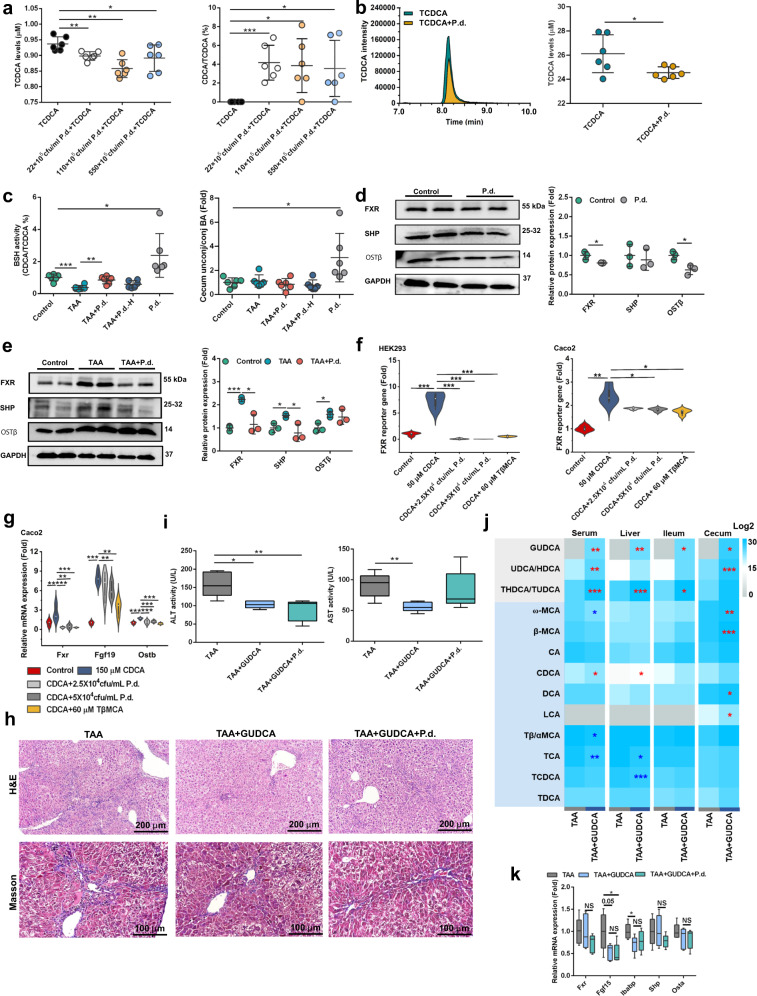
*P. distasonis* increases BSH activity and inhibits ileal FXR signaling. **a** LC-MS analysis revealed that *P. distasonis* promoted the conversion of from taurochenodeoxycholic acid (TCDCA) to chenodeoxycholic acid (CDCA) in culture medium. 22–550 × 10^5^ CFU/mL *P. distasonis* was co-cultured with 25 μM TCDCA in brain-heart infusion fluid medium under anaerobic conditions for 24 h (*n* = 6 biologically independent samples). **b** BSH activity in *P. distasonis* protein (*P. distasonis* protein was extracted from living *P. distasonis* culture medium using sonication. TCDCA was co-incubated with the protein in 3 mM sodium acetate buffer and then CDCA was generated from TCDCA through the BSH activity. TCDCA and CDCA levels were measured by LC-MS (*n* = 6 biologically independent samples)). **c** *P. distasonis* transplantation increased BSH activity and increased cecum content unconjugated/conjugated bile acids in mice. Mice were treated as in Fig. 2a. BA, bile acid; P.d., *P. distasonis*; P.d.-H, heat-killed *P. distasonis*. *n* = 6 per group. **d**, **e** *P. distasonis* decreased ileal farnesoid X receptor (FXR) and its downstream (SHP and OSTβ) protein expression in healthy mice (**d**) and in TAA-induced hepatic fibrosis mice (**e**). Mice were treated as in Fig. 2a (*n* = 3 for dot plot). **f** Luciferase assays of the inhibition of FXR in HEK293 and Caco-2 cells using *P. distasonis* and the FXR inhibitor TβMCA with FXR agonist CDCA co-treatments. HEK293 and Caco-2 cells were treated with 50 μM CDCA, 2.5–5 × 10^4^ CFU/mL *P. distasonis,* and 60 μM TβMCA for 24 h (*n* = 4 biologically independent cells). **g** *P. distasonis* decreased *Fxr* and its target gene (*Fgf19* and *Ostβ*) mRNAs in Caco-2 cells. Caco-2 cells were treated with 150 μM CDCA, 2.5–5 × 10^4^ CFU/mL *P. distasonis,* and 60 μM TβMCA for 24 h (*n* = 6 biologically independent cells). For violin plots **f**, **g**, boxplots represent median with the interquartile range, whiskers indicate adjacent values, violin represents kernel density estimation. **h** Hepatic H&E staining (top) and Masson trichrome staining (bottom) after GUDCA treatment in mice. Mice were treated with TAA (200 mg/kg) for 6 weeks, GUDCA (50 mg/kg), and *P. distasonis* (2 × 10^8^ CFU) for 5 weeks (*n* = 5). H&E staining showed increased inflammatory infiltration, and Masson trichrome staining showed increased hepatic fibrosis in TAA-induced hepatic fibrosis. **i** Serum AST and ALT enzyme activities after GUDCA treatment. Mice were treated as in **h** (*n* = 5). **j** Serum, liver, ileum, and cecum content bile acid levels after GUDCA treatment. Blue color showed higher bile acid levels and gray color showed lower bile acid levels. Heatmap plots were generated by log2 transformation of data. Mice were treated as in **h**. **k** Ileal FXR target gene expression. Mice were treated as in **h** (*n* = 5). In box plot (**i**, **k**), the center line indicates the median, the edges of the box represent the first and third quartiles, and the whiskers extend to span a 1.5 interquartile range from the edges. Data are presented as the mean ± SD. *P < 0.05, **P < 0.01, ***P < 0.001. Source data are provided as a Source Data file.

Since *P. distasonis* was positively correlated with BSH activity in hepatic fibrosis patients (Fig. 1n), we predicted that *P. disasonis* possessed BSH activity. As expected, *P. distasonis* decreased TCDCA levels and increased CDCA/TCDCA ratios by transforming TCDCA to CDCA in culture medium under anaerobic conditions (Fig. 3a). TCDCA was not decreased further with the highest *P. distasonis* employed in Fig. 3a, likely due to growth inhibition by high levels of *P. distasonis*. *P. distasonis* protein, from living *P. distasonis* in the culture medium, showed BSH activity and decreased TCDCA levels, as revealed by in vitro incubations (Fig. 3b). Protein extraction and incubations were carried out based on a previous study^25^. Cecum content BSH activity and unconjugated/conjugated bile acid level were increased after *P. distasonis* treatment in mice, indicating that *P. distasonis* increased BSH activity (Fig. 3c).

Ileal FXR participated in the reabsorption of bile acids. *P. distasonis* inhibited ileal FXR target gene expression (*Fxr*, ileal bile acid binding protein (*Ibabp*) and organic solute transporter α (*Osta*)), and proteins expression (FXR and OSTβ) (Fig. 3d, e, Supplementary Fig. 4k, l). Hepatic *Fxr* mRNA and FXR target gene mRNAs (*Shp*, and *Bsep*) were not decreased by *P. distasonis* (Supplementary Fig. 5a). Dual-luciferase reporter gene assays were performed with HEK293 and Caco-2 cells co-transfected with an FXR expression vector and tk-palinodromic ecdysteroid response element (EcRE)-luciferase expression plasmid, demonstrating that the FXR agonist CDCA significantly increased FXR signaling and *P. distasonis* and the FXR antagonist tauro-β-muricholic acid (TβMCA) significantly decreased FXR signaling (Fig. 3f). *P. distasonis* decreased *Fxr* mRNA and the FXR target gene mRNAs *Fgf19* and *Ostb*, in Caco-2 cells after a 24 h exposure (Fig. 3g). These results showed that *P. distasonis* inhibited ileal FXR directly. GUDCA, a natural antagonist of ileal FXR, could decrease TAA-induced hepatic fibrosis and decrease the protective effects of *P. distasonis* as revealed by H&E and Masson trichrome staining, and AST and ALT activities (Fig. 3h, i). GUDCA treatment decreased serum and hepatic bile acids and promoted the excretion of bile acid in ileum and cecum contents, consistent with the effect of *P. distasonis* (Fig. 3j). These results showed that ileal FXR participated in the protective effects of *P. distasonis*, and inhibition of ileal FXR signaling may promote the excretion of bile acids and improve hepatic fibrosis. Other *P. distasonis* activities such as the production of active metabolites, including indolelactic acid, trimethylamine, cadaverine, lactic acid, and 2-hydeoxybutyric acid were also observed by culture medium metabolomics analysis (Supplementary Fig. 6).

### *P. distasonis* decreases hepatic TCDCA levels and improves activation of HSC through MPT-Caspase-11 pyroptosis

Serum and hepatic TCDCA is important in hepatic fibrosis. In serum of hepatic fibrosis patients, TCDCA and GCDCA were increased 494-fold and 24-fold, respectively (Fig. 1i, j). However, in the TAA-induced fibrosis mouse model, TCDCA was the only bile acid that after increasing by TAA, was decreased by treatment with *P. distasonis* and GUDCA and potentiated by antibiotics in serum and liver (Fig. 2h, 3j, Supplementary Fig. 3b). Serum TCDCA was positively correlated with hepatic fibrosis indexes in patients and in mice (Supplementary Fig.1b, c). Decreased TCDCA in serum and liver after *P. distasonis* treatment resulted from increased *P. distasonis* BSH activity that converts TCDCA to CDCA. Additionally, inhibition of  ileal FXR signaling would decrease the absorption of TCDCA from the intestine (Fig. 3j).

**Fig. 4:**
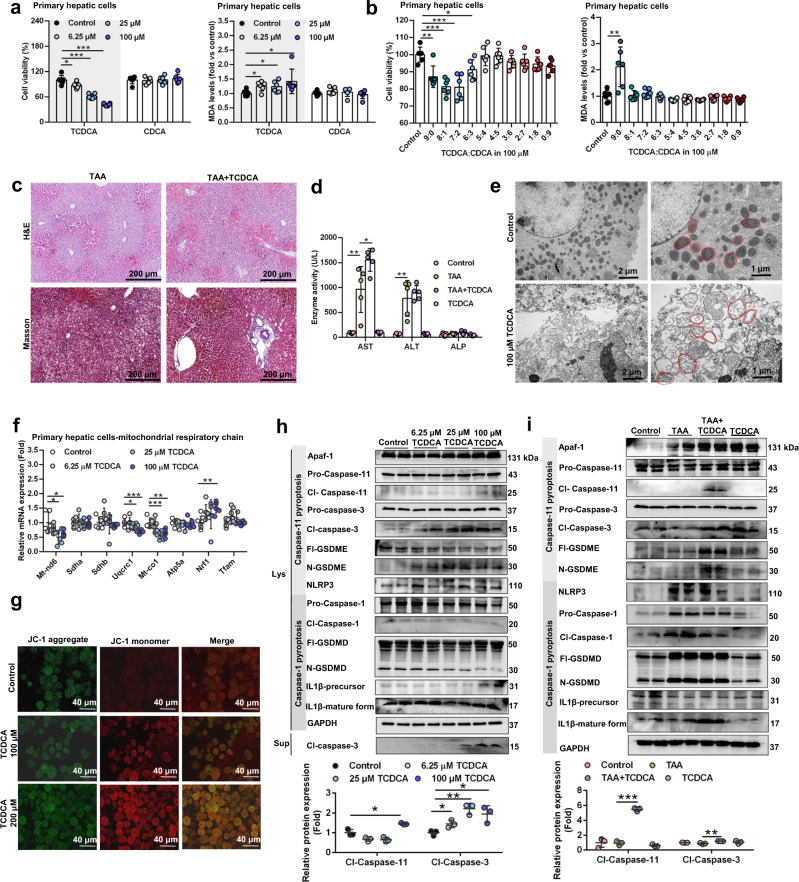
TCDCA potentiates hepatic fibrosis through MPT-Caspase-11 pyroptosis. **a** Cell viability and MDA levels after 6.25–100 μM TCDCA and 6.25–100 μM CDCA treatments in mouse primary hepatic cell for 24 h (*n* = 6 biologically independent cells). BSH activity transformes TCDCA to CDCA, and therefore the toxicity of TCDCA and CDCA was evaluated. TCDCA taurochenodeoxycholic acid, CDCA chenodeoxycholic acid. **b** TCDCA had higher toxicity than CDCA in primary hepatic cells. 100 μM TCDCA and 100 μM CDCA were mixed in the proportion of 9:0, 8:1, 7:2, 6:3, 5:4, 4:5, 3:6, 2:7, 1:8, 0:9 to simulate the gradually increased BSH activity. Then primary hepatic cells were treated with the mixture for 24 h (*n* = 6 biologically independent cells). **c** Hepatic H&E staining (top) and Masson trichrome staining (bottom) after TCDCA (200 mg/kg) treatment for 5 days and TAA (300 mg/kg) treatment for 1 day (*n* = 5). H&E staining showed increased inflammatory infitration, and Masson trichrome staining showed increased hepatic fibrosis in the TAA + TCDCA group. **d** Serum AST, ALT, and ALP enzyme activities after TAA and TCDCA co-treatments. Mice were treated as in **c** (*n* = 5). **e** Representative images of electron microscopy analysis of pyroptosis morphology changes in primary hepatic cells. Cells were stimulated with 100 μM TCDCA for 24 h. Mitochondrial morphologies were marked with red lines. **f** Mitochondrial respiratory chain gene expression in primary hepatic cell. Cells were stimulated with 6.25–100 μM TCDCA for 24 h (*n* = 6 biologically independent cells). **g** JC-1 staining of primary hepatic cells after 100–200 μM TCDCA treatment for 24 h. Confocal microscopy was used, and green fluorescence represented JC-1 aggregates in healthy mitochondria, while red fluorescence represented mitochondrial membrane potential collapse. TCDCA increased red fluorescence and induced mitochondrial damage. **h** Caspase-11 apoptosis pathway (Apaf-1-Caspase-11-Caspase-3-GSDME) and Caspase-1 pyroptosis pathway (NLRP3-Caspase-1-GSDMD/IL1β) protein expression after 6.25–100 μM TCDCA treatment for 24 h in lysates (Lys) and supernatants (Sup) of mouse primary hepatic cells. Cl-Caspase-1/3/11, N-GSDME/GSDMD, and IL1β-mature form were the active forms of the proteins (*n* = 3 for dot plot). **i** Hepatic Caspase-11 and Caspase-1 pyroptosis pathway protein expression in mice. Mice were treated as in **c** (*n* = 3 for dot plot). Data are presented as the mean ± SD. *P < 0.05, **P < 0.01, ***P < 0.001. Source data are provided as a Source Data file.

TCDCA induced toxicity in mouse primary hepatic cells while the deconjugated metabolite CDCA had no effect on these cells (Fig. 4a, b). TCDCA potentiated TAA-induced liver injury in mice as shown by histological analysis, AST levels, proinflammatory factors and hepatic fibrosis mRNA (*Il1*, *Il6*, and *Timp1*) levels, and serum metabolomics (Fig. 4c, d, Supplementary Fig. 7b–d). Furthermore, TCDCA potentiated the toxicity of TAA and deoxycholic acid (DCA) in mouse primary hepatic cells (Supplementary Fig. 8). These results showed that TCDCA contributed to hepatic fibrosis.

Bile acids could induce MPT-Caspase-11 pyroptosis under disease conditions^26^, and therefore we predicted TCDCA-induced hepatic fibrosis through MPT-Caspase-11 pyroptosis. The loss of mitochondrial cristae was revealed by transmission electron microscopy (TEM), altered mitochondrial respiratory chain gene mRNA expression, and the dissipation of mitochondrial transmembrane potential (Δψm) revealing that TCDCA induced MPT in mouse primary hepatic cells (Fig. 4e–g). TCDCA induced the Caspase-11 pyroptosis pathway (apoptosis protease activating factor 1 (Apaf-1)-Caspase-11-Caspase-3-gasdermin E (GSDME)) in mouse primary hepatic cells and in TAA-induced liver injury (Fig. 4h, i, Supplementary Fig. 8k, l). However, the Caspase-1 pyroptosis pathway remained unchanged (NOD-like receptor family pyrin domain containing 3 (NLRP3)-Caspase-1-gasdermin D (GSDMD)/IL1β) (Fig. 4h, i). *P. distasonis* and FMT improved Caspase-11 pyroptosis in TAA-induced hepatic fibrosis mice (Fig. 2g, Supplementary Fig. 3l). These results showed TCDCA-induced MPT and Caspase-11 pyroptosis. GCDCA, another bile acid increased in clinical serum and feces samples, could also induce MPT and Caspase-11 pyroptosis (Fig. 1i, Supplementary Fig. 9a–e). Apoptosis and ferroptosis played unimportant roles in the toxicity of TCDCA and GCDCA (Supplementary Figs. 9f, 10).

**Fig. 5:**
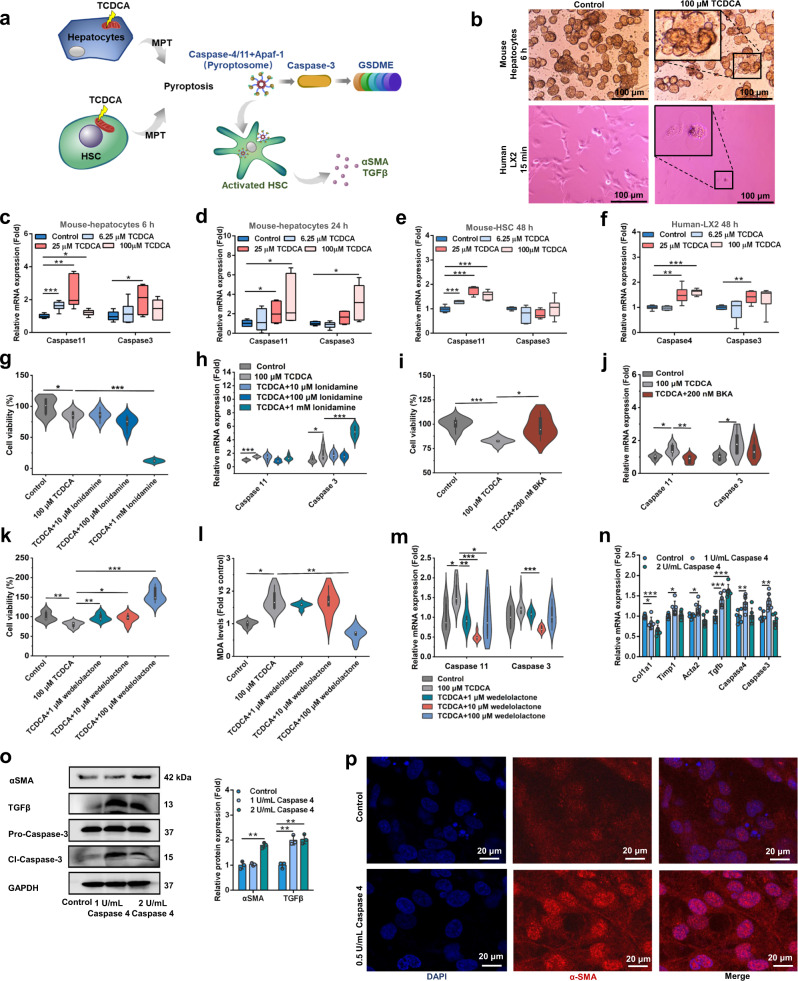
TCDCA activates HSC through increasing the MPT-Caspase-11 pyroptosis pathway in hepatocytes and HSCs. **a** Experimental scheme: MPT-Caspase-11 pyroptosis pathway (Apaf-1-Caspase-11 in mice (Caspase-4 in human)-Caspase-3-GSDME) was activated by TCDCA in hepatocytes and HSC; The increased Caspase-4 protein activated human HSC, released hepatic fibrosis protein αSMA and TGFβ, and finally induced hepatic fibrosis. HSC, hepatic stellate cells. **b** Images of microscope analysis of pyroptotic morphology changes in mouse hepatocyte and human HSC (LX2). Mouse hepatocytes were treated with 100 μM TCDCA for 6 h, and human LX2 were treated with 100 μM TCDCA for 15 min. **c**, **d** Caspase-11 pyroptosis was induced after 6 h (**c**) and 24 h (**d**) TCDCA (6.25–100 μM) treatment in primary hepatocytes (*n* = 6 biologically independent cells). **e**, **f** Caspase-11 pyroptosis was induced by 48 h TCDCA (6.25–100 μM) treatment in mice (**e**) and human (**f**) HSCs (*n* = 6 biologically independent cells). In the box plot (**c**–**f**), the center line indicates the median, the edges of the box represent the first and third quartiles, and the whiskers extend to span a 1.5 interquartile range from the edges. **g**, **h** Cell viability (**g**) and Caspase-11 pyroptosis gene expression (**h**) after MPT agonist lonidamine (10 μM, 100 μM and 1 mM) and TCDCA (100 μM) co-treatments for 24 h in mouse primary hepatocytes (*n* = 6 biologically independent cells). **i**, **j** Cell viability (**i**) and Caspase-11 pyroptosis gene expression (**j**) after MPT antagonist BKA (200 nM) and TCDCA (100 μM) co-treatments for 24 h in mouse primary hepatocytes (n = 6 biologically independent cells). **k**–**m** Cell viability (**k**), MDA level (**l**), and Caspase-11 pyroptosis gene mRNA expression (**m**) after wedelolactone (Caspase-11 inhibitor, 1, 10, and 100 μM) and TCDCA (100 μM) treatments for 24 h in mouse primary hepatocytes (*n* = 6 biologically independent cells). For violin plot (**g**–**m**), boxplots represent median with the interquartile range, whiskers indicate adjacent values, violin represents kernel density estimation. **n**, **o** Hepatic fibrosis and Caspase-4 pyroptosis mRNA (**n** *n* = 6 biologically independent cells) and protein (**o** *n* = 3 for bar graph) expression after 1–2 U/mL Caspase-4 protein treatment for 24 h. **p** αSMA immunofluorescence showed that 0.5 U/mL Caspase-4 protein treatment for 24 h induced the expression of αSMA in LX2 cells. Data are presented as the mean ± SD. *P < 0.05, **P < 0.01, ***P < 0.001. Source data are provided as a Source Data file.

Hepatocytes, HSCs, and Kupffer cells were treated with TCDCA (6.25–100 μM) to evaluate the role of Caspase-11 pyroptosis in different cells. TCDCA-induced Caspase-11 pyroptosis in mouse primary hepatocytes after 6 h and 24 h TCDCA treatment, as shown by increased *Caspase-11* and *Caspase-3* mRNA expression, induced balloon-like bubbles as indicated by microscopic examination and multiple pores formed in the membranes as revealed by TEM (Figs. 4e, 5b–d)^27,28^. Furthermore, TCDCA could induce Caspase-11 pyroptosis in mouse primary HSC and human HSC (LX2 cell) 48 h after TCDCA treatment as revealed by increased *Caspase-11* (*Caspase-4* in humans) and *Caspase-3* mRNA expression and balloon-like bubbles (Fig. 5b, e, f). Caspase-11 pyroptosis was not increased by 6.25–100 μM TCDCA in mice and human primary Kupffer cells 24 h after TCDCA treatment (Supplementary Fig. 11a). These results showed that Caspase-11 pyroptosis was induced by TCDCA in hepatocytes and HSCs rather than in Kupffer cells.

To evaluate the effect of MPT and Caspase-11 pyroptosis in TCDCA-induced liver injury, the MPT agonist lonidamine and antagonist bongkrekic acid (BKA), and Caspase-11 inhibitor wedelolactone were tested in mouse primary hepatocytes (Fig. 5g–m). Lonidamine potentiated TCDCA-induced Caspase-11 pyroptosis, while BKA improved TCDCA-induced Caspase-11 pyroptosis in mouse primary hepatocytes (Fig. 5g–j). The increased cell viability, the decreased malondialdehyde (MDA), and *Caspase-11* and *Caspase-3* mRNA expression showed that the Caspase-11 inhibitor wedelolactone improved TCDCA-induced Caspase-11 pyroptosis in mouse primary hepatocytes (Fig. 5k–m). These results showed that MPT and Caspase-11 pyroptosis participated in TCDCA-induced liver injury.

To evaluate Caspase-11 pyroptosis-activated HSC, LX2 cells were treated with Caspase-4 protein. Caspase-4 protein was internalized by LX2 cells, and increased hepatic fibrosis and pyroptosis gene mRNA and protein expression (Fig. 5n–p). A previous study found that Caspase-1 protein is internalized by LX2 cells^29^. Therefore, TCDCA-induced MPT-Caspase-11 pyroptosis in hepatocytes and HSC, and Caspase-11 pyroptosis activated HSCs (Fig. 5a). On the other hand, TCDCA activated HSC by stimulating the secretion of IL1β, TNFα and TGFβ protein by Kupffer cells, as shown by increased *Timp1* and *Acta2* mRNA expression in HSC after the Kupffer cell supernatant treatment (Supplementary Fig. 11b–e)^30^.

### Celastrol promotes the growth of *P. distasonis*

**Fig. 6:**
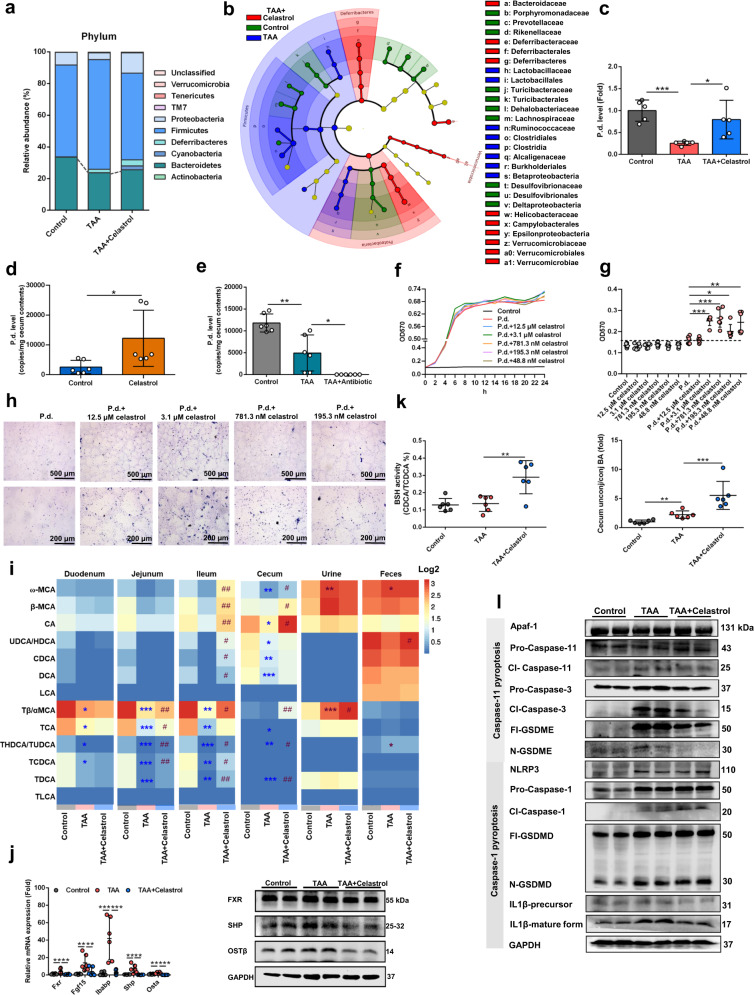
Celastrol protects against TAA-induced hepatic fibrosis through increasing *P. distasonis* level. **a**–**c** Celastrol increased *Bacteroidetes* (**a** phylum), *Bacteroidaceae* (**b** family), and *P. distasonis* (**c** species) in cecum content of mice (*n* = 5 biologically independent animals). Mice were treated with 200 mg/kg TAA for 6 weeks and 10 mg/kg celastrol for 5 weeks. **b** Red, green, blue showed the increased bacteria in the TAA + Celastrol group, control group, and TAA group, respectively. **d** *P. distasonis* was increased in cecum content after 10 mg/kg celastrol treatment for 2 weeks in healthy mice (*n* = 6 biologically independent animals). **e** Antibiotics decreased *P. distasonis* levels in TAA-induced hepatic fibrosis. Mice were treated with 200 mg/kg TAA for 6 weeks and antibiotics (ampicillin, neomycin, metronidazole, and vancomycin) for 5 weeks (*n* = 6 biologically independent animals). **f** Growth curve of *P. distasonis* after treatment with 48.8 nM–12.5 μM celastrol for 24 h in anaerobic incubator (*n* = 3). The original concentration of *P. distasonis* was 22 × 10^5^ CFU/mL. **g**, **h** Biofilm formation of *P. distasonis* was shown by OD value (**g**) and microscopy (**h**) stained with crystal violet (*n* = 6 biologically-independent samples). Cells were treated as in **f**. *P < 0.05, **P < 0.01, ***P < 0.001. **i** Bile acid levels in enterohepatic circulation (duodenum, jejunum, ileum, cecum content, urine and feces). Red color shows higher bile acid levels and blue color shows lower bile acid levels. Heatmap plots were generated by log2 transformation of data. Mice were treated as in **a**–**c**. *n* = 5 per group. *P < 0.05, **P < 0.01, ***P < 0.001 verse Control group; ^#^*P* < 0.05, ^##^*P* < 0.01 verse TAA group. **j** Celastrol decreased ileal *Fxr* mRNA (*n* = 6 biologically independent animals) and FXR protein expression after 10 mg/kg celastrol treatment for 5 weeks. **k** 10 mg/kg celastrol (5 weeks) increased BSH activity and increased cecum content unconjugated/conjugated bile acids in mice (*n* = 6 biologically independent animals). **l** Hepatic Caspase-11 pyroptosis pathway (Apaf-1-Caspase-11-Caspase-3-GSDME) and Caspase-1 pyroptosis pathway (NLRP3-Caspase-1-GSDMD/IL1β) protein expression after TAA and celastrol treatments. Cl-Caspase-1/3/11, N-GSDME/GSDMD, and IL1β-mature form were the active form of the proteins. Data are presented as the mean ± SD. *P < 0.05, **P < 0.01, ***P < 0.001. Source data are provided as a Source Data file.

A previous study observed that celastrol from the roots of *Tripterygium Wilfordii* plant increased *P. distasonis* in high-fat diet-induced obese mice^31^, suggesting that celastrol may promote the growth of *P. distasonis*. In vivo analysis found that celastrol increased *Bacteroidetes* (phylum), *Bacteroidaceae* (family), and *P. distasonis* (species) in the TAA-induced hepatic fibrosis model (Fig. 6a–c, Supplementary Fig. 12a–e). Celastrol could also increase the level of *P. distasonis* in healthy mice (Fig. 6d). In vitro analysis found that celastrol from 48.8 nM to 12.5 μM effectively promoted the growth of *P. distasonis* (Fig. 6f). Quantitative and qualitative experiments revealed that celastrol promoted biofilm formation (Fig. 6g, h). Culture medium metabolomics profiling found that various gut microbiota metabolites were changed in *P. distasonis* following celastrol treatment (Supplementary Fig. 12f, g), and the altered pathways included phenylalanine, tyrosine, and tryptophan biosynthesis, phenylalanine metabolism, and tryptophan metabolism (Supplementary Fig. 12h). These results showed that celastrol could promote the growth of *P. distasonis*.

### Celastrol improves hepatic fibrosis through regulating intestinal bile acid metabolism and attenuating hepatocyte pyroptosis

Celastrol could protect against TAA-induced hepatic fibrosis, including histology, decrease AST and ALT activities, proinflammatory factor expression and hepatic fibrosis gene and protein expression, and serum and liver metabolites (Supplementary Figs. 13a–f, 14e). Celastrol decreased bile acid levels in serum and liver, especially TCDCA, and increased bile acid levels in duodenum, jejunum, ileum, cecum content, urine, and feces, indicating that celastrol promoted the excretion of bile acids (Fig. 6i, Supplementary Figs. 13j, 14e, 15). Consistent with the effect of *P. distasonis*, celastrol increased BSH activity, inhibited ileal FXR, and attenuated Caspase-11 pyroptosis in mice (Fig. 6j–l, Supplementary Figs. 14g, 15i). The data also demonstrated that ileal FXR plays an important role in the protective effects of celastrol on hepatic fibrosis as revealed using *Fxr*-null mice and the intestinal FXR inhibitor GUDCA (Supplementary Fig. 16). After pretreatment with antibiotics, the protective effects of celastrol were decreased (Supplementary Fig. 3m–o), and FMT from 14-day celastrol treated mice could improve TAA-induced hepatic fibrosis (Supplementary Fig. 3g–l) which showed that the gut microbiota has an important role in the protective effects of celastrol. These results revealed that celastrol regulates intestinal bile acid metabolism and protects against hepatic fibrosis.

### *P. distasonis* improves MCD diet-induced hepatic fibrosis

**Fig. 7:**
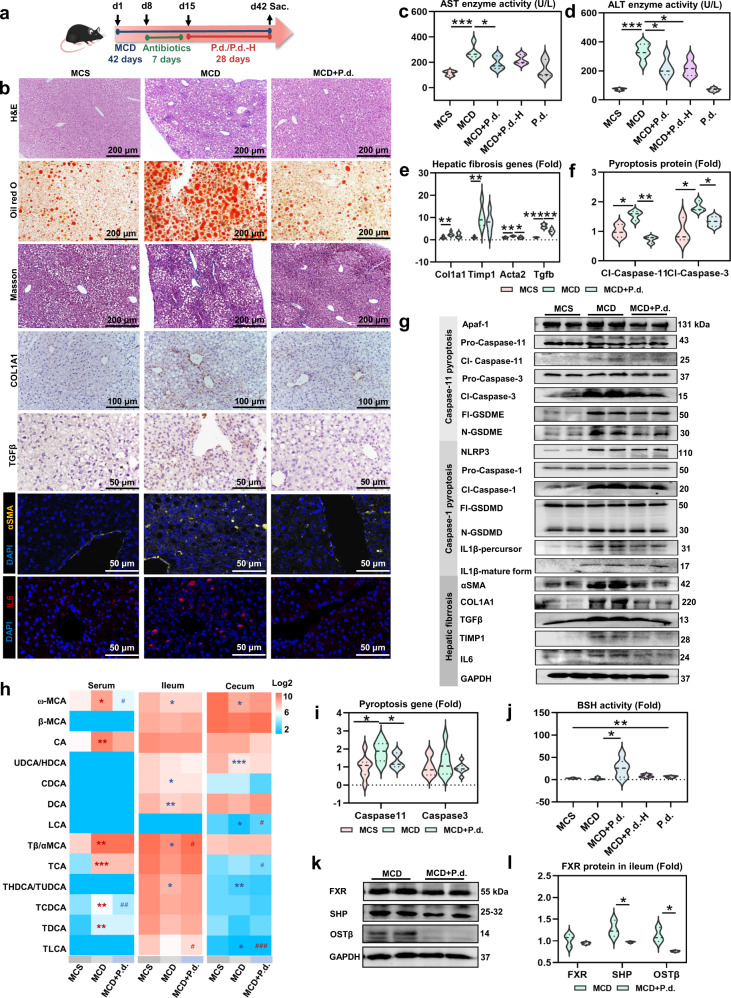
*P. distasonis* improves MCD diet-induced hepatic fibrosis through increasing BSH activity and decreasing ileal FXR. **a** Experimental scheme: mice were treated with MCD diet for 6 weeks. After MCD diet treatment for 1 week, mice were treated with antibiotics (ampicillin, neomycin, metronidazole, and vancomycin) for 1 week. After antibiotics treatment, 2 × 10^8^ CFU *P. distasonis* (P.d.) and heat-killed *P. distasonis* (P.d.-H) were given by oral transplantation once a day for 4 weeks (*n* = 6). **b** Hepatic H&E, Oil red O, Masson trichrome, immunohistochemistry (COL1A1 and TGFβ) and immunofluorescence (αSMA and IL6) stainings. H&E and IL6 immunofluorescence stainings showed increased inflammatory infiltration, Oil red O staining showed increased lipopexia, and Masson trichrome, immunohistochemistry (COL1A1 and TGFβ), and immunofluorescence (αSMA) stainings revealed increased hepatic fibrosis in MCD diet-induced hepatic fibrosis. **c**, **d** Serum AST (**c**) and ALT (**d**) enzyme activity. **e** Hepatic fibrosis gene expression. **f**, **g** Hepatic Caspase-11 pyroptosis pathway (Apaf-1-Caspase-11-Caspase-3-GSDME), Caspase-1 pyroptosis pathway (NLRP3-Caspase-1-GSDMD/IL1β), and hepatic fibrosis protein expression in mice. Cl-Caspase-1/3/11, N-GSDME/GSDMD, and IL1β-mature form were active form of the protein. *P < 0.05, **P < 0.01, ***P < 0.001. **h** Bile acid levels in enterohepatic circulation (serum, ileum, and cecum content). Red color shows higher bile acid levels and blue color shows lower bile acid levels. Heatmap plots were generated by log2 transformation of data. *P < 0.05, **P < 0.01, ***P < 0.001 verse MCS group; ^#^*P* < 0.05, ^##^*P* < 0.01 verses MCD group. **i** Hepatic Caspase-11 pyroptosis gene expression. **j** BSH activity in cecum content. **k**, **l** Ileal FXR protein expression. For violin plots, dotted line represent median with the interquartile range, violin represents kernel density estimation. *P < 0.05, **P < 0.01, ***P < 0.001. Source data are provided as a Source Data file.

*P. distasonis* treatment increased *P. distasonis* levels in MCD diet-induced steatohepatitis and hepatic fibrosis (Supplementary Fig. 17a). H&E, Oil red O, Masson trichrome, immunohistochemistry and immunofluorescence stainings showed that *P. distasonis* improved MCD diet-induced hepatic fibrosis, and decreased lipid accumulation (Fig. 7b). Furthermore, *P. distasonis* improved AST and ALT levels, and hepatic fibrosis gene mRNA and protein expression (Fig. 7c–e, g). Targeted metabolomics analysis revealed that bile acids were decreased in serum and increased in ileum and cecum (Fig. 7h, Supplementary Fig. 17b). *P. distasonis* increased BSH activity, inhibited ileal FXR signaling, and improved Caspase-11 pyroptosis in the MCD diet-induced hepatic fibrosis model (Fig. 7f, g, i–l). Furthermore, celastrol improved MCD diet-induced steatohepatitis and hepatic fibrosis, which increased BSH activity decreased ileal FXR pathway, and alleviated bile acids level in the liver (Supplementary Figs. 18, 19). The protective effects of celastrol were decreased in *Fxr*-null mice, indicating that ileal FXR may play an important role in the development of hepatic fibrosis (Supplementary Fig. 19i, j).

## Discussion

**Fig. 8:**
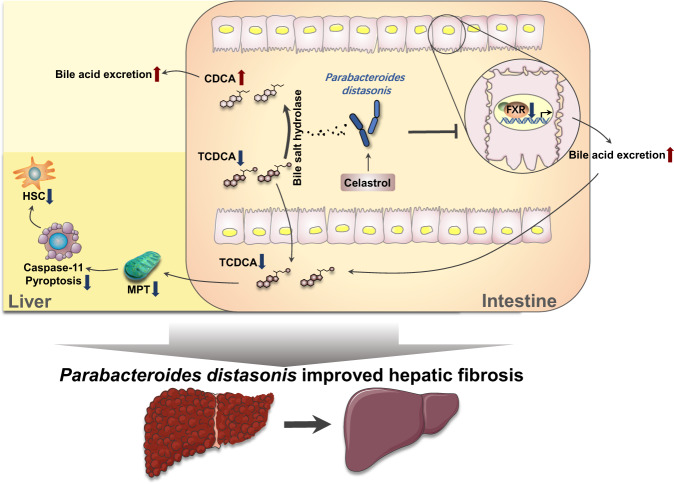
*P. distasonis* improves hepatic fibrosis through increasing BSH activity and inhibiting ileal FXR. (1) Increased BSH activity by *P. distasonis* transformed TCDCA to CDCA. (2) Inhibition of ileal FXR by *P. distasonis* decreased the reabsorption of bile acids in intestine and finally decreased bile acids (e.g., TCDCA) in serum and liver. The increased BSH activity and the inhibited ileal FXR signaling by *P. distasonis* decreased TCDCA in serum and liver. TCDCA activated HSC through MPT-Caspase-11 pyroptosis pathway, and finally induced hepatic fibrosis.

The present study provides evidence that *P. distasonis* contributes to improving hepatic fibrosis through regulating bile acid homeostasis as graphically shown in Fig. 8. The biological function of *P. distasonis* in regulating the generation of bile acids and the targets of the host were demonstrated. The current study determined the role of gut microbiota, bile acid metabolism, and related host targets in hepatic fibrosis. The most common mouse models to study hepatic fibrosis include the chemical-based models (TAA, CCl_4_, ethanol), diet-based models (MCD diet, HFD), a surgery-based model, and a genetically modified model^32^. Since a previous study found that celastrol could protect against CCl_4_- and HFD-induced chronic liver injury^33^, the hepatic fibrosis models induced by TAA and MCD were used in the current study.

Bile acids, diets, intestinal pH, and gastrointestinal motility likely influence the decrease of *P. distasonis* in hepatic fibrosis patients. Bile acids can exert both direct effects and indirect effects on the gut microbiota, including bacterial membrane damage, changes in membrane lipid compositions, and targeting nuclear receptors^34^. Furthermore, diets such as a high-fat western diet and alcohol consumption may play a key role in modulation of the gut microbiota composition through influencing intestinal pH that can promote the development of proinflammatory gut microbiota and increase lipopolysaccharides^35^.

*P. distasonis* is the anaerobic strain belonging to the genus *Parabacteroides*, which commonly colonize the gastrointestinal tract of numerous species. It was found that *P. distasonis* has beneficial and pathogenic effects on human health and was associated with decreased colorectal cancer incidence, obesity, and inflammatory bowel disease and prevent post-calorie restriction weight gain, although it may induce depressive-like behavior in a mouse model of Crohn’s disease^7,12,36^. A previous study found that *P. distasonis* improved triptolide-induced testicular dysfunction through increasing microbial-derived polyamines which could upregulate heat shock protein 70^8^. In a previous study, *P. distasonis* increased unconjugated bile acids, such as UDCA and LCA indicating increased BSH activity in cecum content^12^. Clinical studies reported that NAFLD and neonatal cholestasis disease decreased *P. distasonis* levels^24,37^. In the present study, *P. distasonis* levels were decreased in hepatic fibrosis patients and supplementation of *P. distasonis* could improve TAA- and MCD diet-induced liver fibrosis in mice through regulating bile acid metabolism, indicating that *P. distasonis* plays an important role in hepatic fibrosis. Since research on the protective effects of *P. distasonis* on liver fibrosis is limited, the present study provides prospects to employ *P. distasonis* to improve hepatic fibrosis.

Intestinal FXR and BSH play an important role in human health. VSH#3 probiotics (*L. paracasei*, *L. plantarum*, *L. acidophilus*, *L. delbrueckii* subspecies *L. bulgaricus*, *B. longum*, *B. breve*, *B. infantis*, *Streptococcus thermophilus*), colesevelam and resveratrol promoted the excretion of bile acids and had a beneficial role in metabolic diseases through increasing BSH and inhibiting ileal FXR^38–40^. Another study found decreased BSH activity inhibited ileal FXR through increasing intestinal TβMCA levels^25^. In the present study, *P. distasonis* and celastrol improved hepatic fibrosis by increasing BSH activity and inhibiting ileal FXR. Apart from direct inhibition of *P. distasonis* on ileal FXR, several bile acids such as UDCA, ωMCA, and βMCA were increased in cecum after *P. distasonis* and celastrol treatments and were reported to inhibit intestinal FXR signaling in mice^10,12^, which may contribute to the inhibition of intestinal FXR signaling.

It should be noted that the causal role of bacterial BSH, intestinal FXR signaling, and altered bile acid levels in liver fibrosis after *P. distasonis* administration was not validated in vivo in the current study. However, the role of BSH, conjugated bile acids, and ileal FXR signaling in bile acid homeostasis have been firmly document in earlier studies. Bacterial BSH deconjugates conjugated bile acids secreted from the liver and the BSH inhibitor caffeic acid phenethyl ester increases conjugated bile acids and decreases deconjugated bile acid in the intestine^41,42^. Deconjugated bile acids that result from BSH activity are less soluble and their reabsorption by intestinal epithelial cells is decreased thus they are preferentially eliminated in the feces^43^. Previous studies found that inhibition of ileal FXR or ileal FXR knockout mice increase bile acids in the ileum and decrease bile acids in liver and serum, indicating that inhibition of ileal FXR promotes the fecal elimination of bile acids^20,21,44^. Notably, by inhibition of ileal FXR signaling, the conjugated bile acids TUDCA and GUDCA are markedly increased in healthy mice^20^ and the ileal conjugated bile acids TαMCA and TβMCA are increased and hepatic TCA and TDCA levels and serum TDCA and THDCA levels are decreased in HFD-induced mice^21^. In ileal FXR knockout mice, serum TβMCA, TωMCA, TCA, CDCA and hepatic TαMCA, TβMCA, TωMCA, TCDCA, and TUDCA are decreased^44^. After GUDCA treatment in the present study, bile acid levels in ileum and cecum were increased, and bile acid levels in serum and liver were decreased, thus indicating that inhibition of ileal FXR led to increased bile acid excretion. Finally, administration of *P. distasonis* leads to increased BSH activity, inhibition of intestinal FXR signaling, and decreased TCDCA levels in liver.

Inhibiting the reabsorption and promoting the excretion of bile acid have become a hotspot in drug discovery. Some drugs were developed for treating liver disease such as UDCA, TUDCA, odevixibat, maralixibat, colestyramine, and colesevelam. Notably, UDCA and TUDCA were used for the treatment of PBC through inhibiting intestinal FXR signaling and promoting the excretion of bile acids^45^. In 2021, odevixibat and maralixibat were approved by the FDA for the treatment of pruritus induced by cholestasis. Odevixibat and maralixibat decrease the reabsorption of bile acid through inhibiting ileal apical sodium-dependent bile acid transporter (ASBT), encoded by a FXR target gene, and promote the excretion of bile acids. The bile acid sequestrants colestyramine and colesevelam promote bile acid excretion through complexing with bile acids in the intestinal lumen, and improving pruritus induced by cholestasis clinically and hepatic fibrosis in mice^39^. Therefore, inhibition of FXR and other bile acid transporters in ileum may promote the elimination of bile acids and improve hepatic fibrosis.

The mitochondria is a central hub sensing various stimuli and thereby controlling cell fates, including cell proliferation, differentiation, death, and immunological response^46^. MPT is induced by the persistent opening of the MPT pore, which is usually activated by Ca^2+^ and reactive oxygen species (ROS)^47^. Pyroptosis is mediated by inflammatory caspases which is featured with pore formation on plasma membrane, cell lysis, and swelling^26^. Both Caspase-1-induced inflammasome and Caspase-11-induced pyroptosome were found to play key roles in pyroptosis^26^. The Caspase-11 pryoptosis pathway, including Apaf-1-Caspase-11-Caspase-3-GSDME, is a general sensor of MPT^26^. A previous study found DCA and CDCA activated MPT-Caspase-11 pyroptosis^26^. The present study found TCDCA and GCDCA could activate MPT-Caspase-11 pyroptosis. Hepatic fibrosis is accompanied by activation of Caspase-1 and Caspase-11 pyroptosis. Earlier work found that hepatocytes induce Caspase-1 pyroptosis and release NLRP3 inflammasome and IL1β to the supernatant^29,45^. Hepatocytes activate HSC through internalizing the NLRP3 inflammasome and IL1β^29,45^. The present study found that Caspase-11 pyroptosis occurs in primary hepatocytes and HSC, which activate HSC through releasing the Caspase-11 pyroptosome. These studies suggested that TCDCA may activate HSC through inducing MPT-Caspase-11 pyroptosis.

In the present study, TCDCA levels were increased in serum and feces of hepatic fibrosis patients. It was also reported that TCDCA and GCDCA were increased in NAFLD liver tissue^48^. TCDCA activates HSC through releasing TNFα and TGFβ by Kupffer cells^30^. Therefore, TCDCA may play an important role in the development of hepatic fibrosis. TCDCA induces liver injury in rats and cell toxicity in vitro^49^. Previous studies found that TCDCA initially binds to the mitochondrial outer membrane and subsequently the structure of the inner boundary membrane disintegrates and MPT is induced^50^. Furthermore, TCDCA contributes to apoptosis through the activation of the caspase cascade, PKC/JNK and nuclear factor-κB (NF-κB) pathways^51,52^. The present study demonstrated that TCDCA induces MPT-Caspase-11 pyroptosis, and subsequently Caspase-11 pyroptosis activates HSC. Clinical serum TCDCA levels in chronic liver diseases were 0.7-15.1 μM^53^. The present study found that TCDCA levels in serum were 0.0004-146 μM in the patients with hepatic fibrosis and 246-559 μM in mice with hepatic fibrosis. Notably these levels are higher than the concentration (6.25–100 μM) of TCDCA-induced pyroptosis in hepatic cells in vitro, suggesting that the increased TCDCA could induce pyroptosis during hepatic fibrosis.

Celastrol could protect acute liver injury or cholestasis induced by acetaminophen, α-naphthyl isothiocyanate, CCl_4_ and TAA^54,55^. Celastrol protects against CCl_4_-induced hepatic fibrosis through activating AMPK/SIRT3 signaling^56^. Celastrol also protects against renal fibrosis through upregulating the cannabinoid receptor 2, improving cardiac fibrosis through inhibiting microRNA-21/ERK1/2, and improving pulmonary fibrosis through activating autophagy^57,58^. Here, celastrol was found to protect against TAA- and MCD diet-induced hepatic fibrosis through increasing *P. distasonis* in mice. An earlier study found that celastrol reduced intestinal lipid absorption to antagonize obesity by resetting the gut microbiota under HFD feeding^59^. Antibiotics treatment blocked the celastrol-induced lipid-lowing effect and FMT from celastrol-treated obese mice reduced the body weight gain of HFD-fed mice suggesting a critical role for the gut microbiota in mediating the anti-obesity effects of celastrol under HFD^59^. Increased *P. distasonis* was observed after celastrol and HFD co-treatments^31,59^. The present work demonstrated that celastrol increased *P. distasonis* levels directly in vivo and in vitro and loss of gut microbiota decreased the protective effects of celastrol, indicating that the protective effect of celastrol on liver fibrosis is closely associated with its promotion of *P. distsonis* growth. Similarly, celastrol could inhibit intestinal FXR and increase the excretion of bile acids, which decreases the levels of hepatic TCDCA and prevents TAA- and MCD diet-induced hepatic fibrosis. The function of *P. distasonis* may also be influenced by celastrol. Celastrol promots the production of indoleacrylic acid, phenylacetylglutamine, and tryptophan in *P. distasonis* in vitro, which can be explored by further studies on the prokaryotic transcriptome (Supplementary Fig. 6b). A previous study found that polysaccharide increased the levels of short-chain fatty acids of *Lactobacillus faecis* by regulating the expression of *idh*, *metE*, *adh2*, mvK in *L. faecis* as revealed by study of the *Lactobacillus faecis* transcriptome and proteome^60^. Thus celastrol may also influence related genes in *P. distasonis*.

In summary, *P. distasonis* decreased hepatic TCDCA levels, increased BSH activity, inhibited ileal FXR and improved hepatic fibrosis. The decrease of hepatic TCDCA improved the activation of HSC by decreasing MPT-Caspase-11 pyroptosis in hepatocytes. Taken together, these findings suggest that supplementation with *P. distasonis* may be a promising means to ameliorate hepatic fibrosis, however, whether patients with hepatic fibrosis might benefit from supplementation with *P. distasonis* needs to be investigated in further studies.

## Methods

### Patients

Sample 1: Sixty-two patients with hepatic fibrosis and 25 healthy volunteers were recruited to collect serum samples. Sample 2: Seventeen patients with hepatic fibrosis and 10 healthy volunteers were recruited to collect fecal samples. Sample 3: Ten patients with hepatic fibrosis and 10 healthy volunteers were recruited to collect the serum and fecal samples. The patient information is summarized in Supplementary Tables 1–3. All volunteers were evaluated by clinicians during the study ensuring that they remained in good health. Written informed consents were obtained from each participant. The patients and healthy volunteers had no participant compensation. The study protocol conforms to the ethical guidelines of the 1975 Declaration of Helsinki. The study protocol was approved by the Conjoint Health Research Ethics Board of the Secondary Affiliated Hospital of Kunming Medical University (Registration number: PJ-2017-25) and the Conjoint Health Research Ethics Board of West China Hospital, Sichuan University (Registration number: ChiCTR2200067222).

### Animals

All animal experiment protocols were approved by the Animal Care and Use Committee of West China Hospital, Sichuan University (20220217001). Male 6-week-old *Fxr*-null mice (C57BL/6 J background) were previously described^61^. Male 6-week-old C57BL/6 J mice were purchased from GemPharmatech Co., Ltd. (Jiangsu, China) and maintained under a standard 12-h light/12-h dark cycle environment with free access to water and rodent chow (Double lion experimental animal feed technology Co., LTD.). Mice were maintained in a temperature 22 ± 1 °C and humidity-controlled room (40–65%).

To investigate the relationship between *P. distasonis* and the severity of hepatic fibrosis, mice were randomly assigned into six groups (*n* = 6): (1) Con-6 week; (2) TAA-6 week; (3) Con-8 week; (4) TAA-8 week; (5) Con-10 week; (6) TAA-10 week. TAA groups were treated with TAA for 6, 8, and 10 weeks (i.p., 200 mg/kg dissolved in physiologic saline solution, three times per week). To determine the role of gut microbiota in TAA-induced hepatic fibrosis, mice were randomly assigned to 3 groups (*n* = 6): (1) Control; (2) TAA; (3) TAA + Antibiotic. TAA and TAA + Antibiotic groups were treated with TAA for 6 weeks (200 mg/kg)^58^. The mixture of antibiotics including ampicillin (0.25 mg/mL), neomycin (0.25 mg/mL), metronidazole (0.25 mg/mL), and vancomycin (0.125 mg/mL) were dissolved in water and supplied ad libitum to eliminate the gut microbiota^8^. After TAA treatment for 1 week, the TAA + Antibiotic group was treated with antibiotics for 5 weeks.

*P. distasonis*, obtained from American Type Culture Collection (ATCC8503, USA), was cultured in brain-heart infusion fluid medium (Huankai, China) and tryptic soy agar/broth solid medium (with 5% sheep blood, Solarbio, China). To determine the protective effect of *P. distasonis* on TAA-induced hepatic fibrosis, the mice were randomly assigned to 5 groups (*n* = 6): (1) Control; (2) TAA; (3) TAA + *P. distasonis* (TAA + P.d.); (4) TAA + heat-killed *P. distasonis* (TAA + P.d.-H); (5) *P. distasonis* (P.d.). TAA, TAA + P.d., and TAA + P.d.-H groups were treated with TAA for 6 weeks (200 mg/kg). After TAA treatment for 1 week, all groups were treated with antibiotics for 1 week. After antibiotics treatment, the TAA + P.d. and P.d. groups were given *P. distasonis* by oral transplantation for 4 weeks (i.g., 2×10^8^ CFU dissolved in PBS, once a day)^8^; heat-killed *P. distasonis* was parallelly treated.

To investigate the protective effects of celastrol on TAA-induced hepatic fibrosis, wild-type, and *Fxr*-null mice were randomly assigned to 3 groups (*n* = 6): (1) Control; (2) TAA; (3) TAA + Celastrol. TAA and TAA + Celastrol groups were treated with TAA for 6 weeks (200 mg/kg). After TAA treatment for 1 week, TAA + Celastrol group was orally treated with celastrol for 5 weeks (10 mg/kg dissolved in 1% DMSO + 2% Tween 80 + 97% water, three times per week)^54^. Furthermore, to investigate the effect of celastrol on bile acid metabolism in healthy mice, mice were orally treated with celastrol for 1 day (*n* = 5) and 2 weeks (*n* = 6), respectively (10 mg/kg, three times per week).

To validate the function of gut microbiota on the protective effect of celastrol, the mice were randomly assigned to 5 groups (*n* = 6): (1) Control; (2) TAA; (3) TAA + Celastrol; (4) TAA + Antibiotic; (5) TAA + Antibiotic+Celastrol. TAA, TAA + Celastrol, TAA + Antibiotic and TAA + Antibiotic+ Celastrol groups were treated with TAA for 6 weeks (200 mg/kg). After TAA treatment for 1 week, the TAA + Antibiotic+Celastrol group was co-treated with celastrol (10 mg/kg) and antibiotics for 5 weeks.

To determine the function of gut microbiota on the protective effect of celastrol, mice were randomly assigned to 4 groups: (1) Control; (2) TAA; (3) TAA + microbial transplantation from control group (TAA + Con-FMT); (4) TAA + microbial transplantation from celastrol 10 mg/kg group (TAA + Cela-FMT). Fresh gut microbiota from the donor mice was prepared by suspending 50 mg cecum content in 1 mL PBS followed by centrifugation at 500 g. The volume of microbial suspension was 200 μL/mouse/day^8^. The donor mice in the TAA + Con-FMT group were healthy mice. The donor mice in the TAA + Cela-FMT group were treated with celastrol mice for 2 weeks. The TAA, TAA + Con-FMT, and TAA + Cela-FMT groups were treated with TAA for 6 weeks (200 mg/kg). After TAA treatment for 1 week, all groups were treated with antibiotics for 1 week. After antibiotics treatment, FMT was conducted for 4 weeks starting from the 15th day in the TAA + Con-FMT and TAA + Cela-FMT groups^59^.

To investigate the toxic effect of TCDCA on liver injury, the mice were randomly assigned into four groups (*n* = 6): (1) Control; (2) TAA; (3) TAA + TCDCA; (4) TCDCA. TAA + TCDCA and TCDCA groups were orally treated with TCDCA (200 mg/kg) for 5 consecutive days^20,62^. After TCDCA treatment for 4 days, TAA and TAA + TCDCA groups were given a single intraperitoneal dose of TAA (300 mg/kg dissolved in physiologic saline solution)^54^.

To investigate the effect of intestinal FXR on hepatic fibrosis, mice were randomly assigned into 4 groups (*n* = 5): (1) TAA; (2) TAA + GUDCA; (3) TAA + GUDCA + Cela; (4)TAA + GUDCA + P.d. All groups were treated with TAA for 6 weeks (200 mg/kg). After TAA treatment for 1 week, TAA + GUDCA group was orally treated with GUDCA for 5 weeks (50 mg/kg dissolved in 2% DMSO + 2% Tween 80 + 96% water, once a day), TAA + GUDCA + Cela group was co-treated with GUDCA (50 mg/kg) and celastrol (10 mg/kg) for 5 weeks, and TAA + GUDCA + P.d. group was co-treated with GUDCA (50 mg/kg) and *P. distasonis* (2 × 10^8^ CFU) for 5 weeks^20^.

To determine the protective effect of *P. distasonis* on MCD diet-induced hepatic fibrosis, the mice were randomly assigned to 5 groups (*n* = 6): (1) MCS; (2) MCD; (3) MCD + *P. distasonis* (MCD + P.d.); (4) MCD + heat-killed *P. distasonis* (MCD + P.d.-H); (5) *P. distasonis* (P.d.). MCD, MCD + P.d., and MCD + P.d.-H groups were fed a MCD diet for 6 weeks (TP-3001, Trophic Animal Feed High-Tech Co., Ltd, China)^63^. The control MCS group was treated with MCS diet for 6 weeks. After MCD diet treatment for 1 week, all groups were administered antibiotics for 1 week. After antibiotics treatment, the MCD + P.d. and P.d. groups were given *P. distasonis* by oral transplantation for 4 weeks (i.g., 2 × 10^8^ CFU dissolved in PBS, once a day)^8^; heat-killed *P. distasonis* was parallelly treated. To investigate the protective effect of celastrol on MCD diet-induced hepatic fibrosis, the wild-type mice (*n* = 8) and *Fxr*-null mice (*n* = 6) were randomly assigned to three groups: (1) MCS; (2) MCD; (3) MCD + Celastrol. MCD and MCD + Celastrol groups were treated with MCD diet for 6 weeks. After MCD diet treatment for 1 week, the MCD + Celastrol group was orally treated with celastrol for 5 weeks (10 mg/kg, three times per week). MCD diet (TP3005G) and MCS diet (TP3005GS) were purchased from Trophic Animal Feed High-Tech Co., Ltd, China.

### 16 S rRNA analysis

Microbial genomic DNA in cecum content was extracted using the Stool Genomic DNA kit (CWBIO, China). High-throughput sequencing and analysis were performed by the Beijing Genomics Institute (Shenzhen, China). All the qualified DNA was used to construct a library. Paired-end reads were generated with the Illumine HiSeq/MiSeq platform, and the reads with sequencing adapters, N base, poly base, and low quality were filtered. Species composition and abundance analysis was then carried out.

### Growth curve, biofilm assay, and culture medium metabolomics

*P. distasonis* was cultured in brain-heart infusion fluid medium under anaerobic conditions at 37 °C, and the value of OD570 reached 0.6. To determine the effects of celastrol on the growth of *P. distasonis*, 2% (v/v) fluid medium containing *P. distasonis* was then added to fluid medium containing different concentrations of celastrol (48.8 nM–12.5 μM based on the maximum solubility) for 24 h. Absorbance values were measured at 570 nm^60^. Crystal violet was used to quantify biofilm formation^60^. Culture medium samples for metabolomics was prepared as follows: 500 μL culture medium were minced with 500 μL 50% aqueous acetonitrile. After centrifugation at 16,000 × *g* for 20 min, 100 μL supernatant were minced with 300 μL acetonitrile. A 5 μL aliquot was injected into the LC-MS system after centrifugation at 16,000 × *g* for 20 min. 5 μM chlorpropamide was used as the internal standard.

### UHPLC-Q exactive plus MS analysis

The preparation of plasma, liver, duodenum, jejunum, ileum, cecum content, feces, and urine samples were carried out using the methods described previously^64,65^. The liquid chromatography system obtained a Vanquish autosampler, Vanquish detector, Vanquish pump (Thermo Fisher, USA) equipped with a ACQUITY UPLC HSS T3 column (2.1 × 100 mm, 1.8 μM, Waters, USA). The Vanquish UHPLC-Q Exactive plus MS (Thermo Fisher, USA) was collected in both positive and negative modes, which was operated in full-scan mode at m/z 60 to 900. The mobile phase comprised 0.1 % aqueous formic acid and acetonitrile containing 0.1% formic acid. The flow rate was 0.3 mL/min with a gradient ranging from 2% to 98% acetonitrile in a 17 min run. Principal component analysis (PCA) and orthogonal projection to latent structures-discriminant analysis (OPLS-DA) were carried out using SIMCAP 13.0 (Umetrics, Kinnelon, NJ). Targeted analysis was carried with a Thermo Xcalibur Qual Browser. The chemical structures of the altered metabolites were identified by authentic standard and MS/MS fragmentation patterns in Supplementary Tables 4–9.

### QPCR, western blot, immunohistochemistry, and immunofluorescence analyses

Primer sets for RNA analysis are shown in Supplementary Table 10. The following antibodies were used in western blot, immunohistochemistry and immunofluorescence: Apaf-1 (sc-65891, Santa Cruz Biotechnology, USA; WB 1:200 dilution), Caspase-11 (ab180673, Abcam, UK; WB 1:1000 dilution), Caspase-3 (9662 s, Cell Signaling Technology, USA; WB 1:1000 dilution), GSDME (ab215191, Abcam, UK; WB 1:1000 dilution), αSMA (D4K9N, 19245 s, Cell Signaling Technology, USA; WB 1:1000 dilution; IF 1:100 dilution; IHC 1:800 dilution), COL1A1 (E8F4L, 72026 s, Cell Signaling Technology, USA; WB 1:1000 dilution; IHC 1:100 dilution), TIMP1 (ab179580, Abcam, UK; WB 1:1000 dilution), TGFβ (ab215715, Abcam, UK; WB 1:1000 dilution; IHC 1:200 dilution), IL6 (D5W4V, 12912 s, Cell Signaling Technology, USA; WB 1:1000 dilution; IF 1:200 dilution), IL1β (D3H1Z, 12507 s, Cell Signaling Technology, USA; WB 1:1000 dilution), FXR (sc-25309, Santa Cruz Biotechnology, USA; WB 1:100 dilution), SHP (ab232841, Abcam, UK; WB 1:500 dilution), OSTβ (bs-2128R, Bioss, China; WB 1:1000 dilution), NLRP3 (D4D8T, 15101 s, Cell Signaling Technology, USA; WB 1:1000 dilution), Caspase-1 (E2G2I, 89332 s, Cell Signaling Technology, USA; WB 1:1000 dilution), GSDMD (sc-393656, Santa Cruz Biotechnology, USA; WB 1:200 dilution), CK19 (ab254186, Abcam, UK; IF 1:100 dilution), CD31 (ab7388, Abcam, UK; IF 1:100 dilution), GPX4 (sc-166570, Santa Cruz Biotechnology, USA; WB 1:200 dilution), COX2 (sc-376861, Santa Cruz Biotechnology, USA; WB 1:200 dilution) and GAPDH (14C10, 2119 s, Cell Signaling Technology, USA; WB 1:1000 dilution).

### Measurement of BSH activity

To evaluate the BSH activity of *P. distasonis* in vivo, BSH activity in the feces and cecum content was measured by LC-MS based on the generation of CDCA from TCDCA^25^. The unconjugated/conjugated bile acid ratios in serum, cecum content, and feces were used to show BSH activity in vivo^40^. To evaluate the BSH activity of *P. distasonis* in vitro, *P. distasonis* was co-cultured with 25 μM TCDCA in brain-heart infusion fluid medium under anaerobic conditions at 37 °C for 24 h, and the generation of CDCA from TCDCA in the medium was measured by LC-MS.

### Biochemical assay and histological examination

AST, ALT, ALP, catalase (CAT), MDA, total cholesterol (TC), total triglycerides (TG), Fe^2+^/Fe^3+^ (Nanjing Jiancheng Bioengineering Institute, China), lactate dehydrogenase (LDH, Cayman, USA), mitochondrial membrane potential (Beytime, Shanghai, China) were measured following the manufacturer’s instructions. H&E, Oil red O, Sirius red, and Masson trichrome staining were carried out.

### Isolation of hepatocytes, HSCs, and Kupffer cells

Primary hepatic cells, including hepatocytes, HSCs, and Kupffer cells were obtained from mouse liver by collagenase perfusion and percoll centrifugation^66–68^. Mouse primary HSC and Kupffer cells were identified by Oil red O staining and F4/80 antibody, respectively. Human LX2 cell and human primary Kupffer cells were obtained from Procell Life Science&Technology Co., Ltd. (CL-0560) and Qingqi biotech company (BFN608007039, China), respectively.

Mouse primary hepatic cells were cultured in William E medium containing 10% fetal bovine serum. Cells were harvested 24 h after incubation with TCDCA (6.25, 25, and 100 μM)^55^, GCDCA (6.25–600 μM), CDCA (6.25, 25, and 100 μM), TAA (model drug, 16 mM)^69^ and DCA (model drug, 200 μM)^55^. Mouse primary hepatocytes were cultured in William E medium containing 10% fetal bovine serum, and the drug concentration was selected as follows: 6.25, 25, and 100 μM TCDCA^55^, 1, 10, and 100 μM Caspase-11 inhibitor wedelolactone^70^, 10 μM, 100 μM and 1 mM MPT agonist lonidamine^26^, 200 nM MPT antagonist BKA^26^. Mouse primary HSC and human LX2 cell were cultured in DMEM medium containing 10% fetal bovine serum which were harvested after incubation with TCDCA (6.25, 25, and 100 μM)^55^, Caspase-4 protein (0.5, 1, and 2 U/mL, ab51994, Abcam, UK)^71^, Kupffer cell supernatant treated with 100 μM TCDCA (half of the medium was exchanged with the supernatant in 12-well plate)^66^. Mouse and human primary Kupffer cells were cultured in DMEM medium containing 10% fetal bovine serum which were harvested after incubation with TCDCA (6.25, 25, and 100 μM)^55^.

### Transmission electron microscopy (TEM)

Primary hepatic cells after 100 μM TCDCA treatment were collected and fixed at 4 °C in 0.1 M phosphate buffered 3% glutaraldehyde and postfixed in 1% osmium tetroxide in the same buffer. After rinsing in distilled water, the cells were dehydrated in graded acetone series and embedded in SPI-Pon812. Ultrathin sections were cut and attached to copper grids with Formvar film, then stained with 2% uranyl acetate and Reynolds lead citrate, and examined under a Hitachi HT7800 electron microscope.

### Luciferase reporter gene assays

For luciferase reporter gene assays, HEK293 and Caco-2 cells were transfected with FXR, tk-EcRE-luciferase and renilla-luciferase^72^. Lipofectamine 2000 was used in the transection procedure (Invitrogen, Grand Island, NY). Transfected cells were treated with FXR agonist CDCA (50 μM), *P. distasonis* (2.5 × 10^4^ and 5 × 10^4^ CFU/mL) and FXR inhibitor TβMCA (60 μM) for 24 h^2^. Furthermore, Caco-2 cells were treated with FXR agonist CDCA (150 μM), *P. distasonis* (2.5 × 10^4^ and 5 × 10^4^ CFU/mL) and FXR inhibitor TβMCA (60 μM) for 24 h^2^, and the gene expression of *Fxr*, *Fgf19,* and *Ostb* were evaluated. HEK293 (CRL-1573) and Caco-2 (HTB-37) were obtained from ATCC.

### Statistics

Data analyses such as ROC curves, correlation analysis, and Sankey plots were performed by GraphPad Prism 6.01 (GraphPad, USA) and OriginPro 9.0 (OriginLab, USA). Pathway enrichment was conducted with MetaboAnalyst 4.0. Differences between two groups were tested using the Student’s *t* test. Differences among multiple groups were tested using one-way ANOVA followed by Dunnett’s post hos comparisons. *P* value <0.05 was considered significant. Two times each experiment was repeated independently with similar results.

### Reporting summary

Further information on research design is available in the Nature Portfolio Reporting Summary linked to this article.

## Supplementary information

Supplementary Information

Reporting Summary

## Data Availability

The 16 S rRNA data generated in this study were deposited in the NCBI SRA database under accession code PRJNA911510. The mass spectrometry data generated in this study were deposited in the MetaboLights under accession code MTBLS6742, MTBLS6732, and MTBLS6728. The previously published publicly available 16 S rRNA data were obtained from the Genome Sequence Archive in BIG Data Center, Beijing Institute of Genomics, Chinese Academy of Sciences, under accession code CRA001920[https://bigd.big.ac.cn/gsa]^24^. All other data generated or analyzed during this study are included in this published article (and its supplementary information files). Source data are provided with this paper.

## Source data

Source Data
