# Supplementary material for: Parabacteroides distasonis ameliorates hepatic fibrosis potentially via modulating intestinal bile acid metabolism and hepatocyte pyroptosis in male mice
Source: Nat Commun. 2023 Apr 1;14:1829. doi: 10.1038/s41467-023-37459-z (PMC10067939; doi:10.1038/s41467-023-37459-z)
Supplement: Supplementary file 1 — Supplementary Information [file 41467_2023_37459_MOESM1_ESM.pdf]

Parabacteroides distasonis ameliorates hepatic fibrosis through modulating intestinal bile acid metabolism and hepatocyte pyroptosis in male mice

Qi Zhao[1], Man-Yun Dai[1,2,3], Ruo-Yue Huang[1], Jing-Yi Duan[1], Ting Zhang[1,2,3], Wei-Min Bao[4], Jing-Yi Zhang[5], Shao-Qiang Gui[5], Shu-Min Xia[5], Cong-Ting Dai[5], Ying-Mei Tang[5,*], Frank J. Gonzalez[6], Fei Li[1,7]

[1]Laboratory of Metabolomics and Drug-Induced Liver Injury, Department of Gastroenterology & Hepatology, Frontiers Science Center for Disease-related Molecular Network, West China Hospital, Sichuan University, Chengdu, 610041, China

[2]State Key Laboratory of Phytochemistry and Plant Resources in West China, Kunming Institute of Botany, Chinese Academy of Sciences, Kunming, 650201, China

[3]University of Chinese Academy of Sciences, Beijing,100049, China

[4]Department of Gastroenterology, The second Affiliated Hospital of Kunming Medical University, Kunming 650101, China

[5]Department of General Surgery, The First People's Hospital of Yunnan Province, Kunming 650101, China

[6]Center for Cancer Research, National Cancer Institute, National Institutes of Health, Bethesda, MD, 20892, USA

[7]Sichuan University-Oxford University Huaxi Gastrointestinal Cancer Center, West China Hospital, Sichuan University, Chengdu, 610041, China

Supplementary Tables

Supplementary Table 1. Sample 1: clinical characteristics of the heathy people and hepatic fibrosis patients who provided the serum samples.

	Healthy people (n=25)	Patient (n=62)
Gender (M/F)	11/14	34/28
Age (years)	49.1±17.3	55.7±12.6
ALP (U/L)	101±58.8	163±137
GGT (U/L)	31.0±27.0	120±125
TBA (μM)	12.5±8.2	84.4±107
TBil (μM)	5.5±5.4	71.2±86.0
Child score	——	8.9±2.1
MELD score	——	34.0±7.3
Etiology (HBV/other)	——	22/40

Notes: ALP, alkaline phosphatase; GGT, γ-glutamyl transpeptidase; TBA, total bile acid; TBil, total bilirubin; HBV, hepatitis B virus; M, male; F, female; Child and MELD scores were two standards to evaluate the severity degree of liver cirrhosis.

Supplementary Table 2. Sample 2: clinical characteristics of the healthy people and hepatic fibrosis patients who provided the feces samples.

	Healthy people (n=10)	Patient (n=17)
Gender (M/F)	5/5	7/10
Age (years)	52.1±9.4	53.7±6.9
ALP (U/L)	68.6±20.08	147±53.6
GGT (U/L)	19.3±6.9	111±141
TBA (μM)	12.9±6.0	110±137
TBil (μM)	5.7±5.9	98.7±119
Child score	——	7.3±1.8
MELD score	——	11.0±7.5
Etiology (HBV/other)	——	6/11

Notes: ALP, alkaline phosphatase; GGT, γ-glutamyl transpeptidase; TBA, total bile acid; TBil, total bilirubin; HBV, hepatitis B virus; M, male; F, female; Child and MELD scores were two standards to evaluate the severity degree of liver cirrhosis.

Supplementary Table 3. Sample 3: clinical characteristics of the healthy people and hepatic fibrosis patients who provided both the serum and the feces samples.

	Healthy people (n=10)	Patient (n=10)
Gender (M/F)	5/5	7/3
Age (years)	52.1±9.4	52.6±9.3
ALP (U/L)	68.6±20.1	165±35.2
GGT (U/L)	19.3±6.9	112±150
TBA (μM)	12.9±6.0	115±6.0
TBil (μM)	5.7±5.9	74.0±9.8
Child score	——	8.3±2.5
MELD score	——	14.7±10.7
Etiology (HBV/other)	——	6/4

Notes: ALP, alkaline phosphatase; GGT, γ-glutamyl transpeptidase; TBA, total bile acid; TBil, total bilirubin; HBV, hepatitis B virus; M, male; F, female; Child and MELD scores were two standards to evaluate the severity degree of liver cirrhosis.

Supplementary Table 4. *P. distasonis* improves serum metabolites in TAA-induced hepatic fibrosis.

	Metabolites	Fomula	Mass	RT (min)	Mode	Main fragments	Error (ppm)	Up/down (TAA vs control)
1	Indole-3-acetylglycine	$C_{12}H_{12}N_2O_3$	232.0848	5.38	ESI-	74;116;187	3.2	down
2	Maleic acid	$C_4H_4O_4$	116.0110	0.98	ESI-	71	1.5	down
3	Capryloylglycine	$C_{10}H_{19}NO_3$	201.1365	6.95	ESI-	74;156	1.3	down
4	Malic acid*	$C_4H_6O_5$	134.0215	0.99	ESI-	71;73;115	7.3	down
5	Glutamylphenylalanine	$C_{14}H_{18}N_2O_5$	294.1216	4.11	ESI-	147;164	5.4	down
6	Ursodeoxycholic acid*	$C_{24}H_{40}O_4$	392.5720	8.85	ESI-	355;373	9.0	up
7	Hyodeoxycholic acid*	$C_{24}H_{40}O_4$	392.5720	8.85	ESI-	346;372	0.5	up
8	Taurochenodesoxycholic acid*	$C_{26}H_{45}NO_6S$	499.7046	8.09	ESI-	80;124	0.6	up
9	Deoxycholic acid*	$C_{24}H_{40}O_4$	392.5720	10.21	ESI-	327;345;355;373	5.3	up
10	L-Tryptophan*	$C_{11}H_{12}N_2O_2$	204.0899	3.63	ESI+	146;188	8.3	down
11	2-Hydroxycinnamic acid	$C_9H_8O_3$	164.0473	1.04	ESI+	91;103;123;147	8.6	down
12	L-Tyrosine*	$C_9H_{11}NO_3$	181.0739	1.04	ESI+	91;119;123;136	-5.3	down
13	N2-γ-glutamylglutamine	$C_{10}H_{17}N_3O_6$	275.1117	0.91	ESI+	84;130;147	4.4	down
14	LPC22:6	$C_{30}H_{50}NO_7P$	567.3325	10.49	ESI+	104;184;258;550	6.2	down
15	LPC15:0	$C_{23}H_{48}NO_7P$	481.3168	10.36	ESI+	104;184;464	1.1	down
16	LPC17:0	$C_{25}H_{52}NO_7P$	509.3481	11.69	ESI+	104;184;258;492	-1.7	down
17	LPC20:4	$C_{28}H_{50}NO_7P$	543.3325	10.36	ESI+	104;184;258;526	6.4	down
18	LPC16:0*	$C_{24}H_{50}NO_7P$	495.3325	10.99	ESI+	104;184;258;478	8.1	down
19	1-Naphthylamine	$C_{10}H_9N$	143.0735	3.63	ESI+	115;116;117;128	-2.4	down
20	Indole-3-carboxaldehyde	C_9H_7NO	145.0528	3.63	ESI+	118	-2.8	down
21	Sphinganine 1-phosphate	$C_{18}H_{40}NO_5P$	381.2644	9.99	ESI+	266;284	9.3	up
22	Bilirubin	$C_{33}H_{36}N_4O_6$	584.2635	7.42	ESI+	299	-5.7	up
23	L-Arginine*	$C_6H_{14}N_4O_2$	174.1117	0.82	ESI+	60;70;116;130;159	2.1	down
24	Hydroxybutyrate	$C_4H_8O_3$	104.0473	1.04	ESI-	59	-0.7	up
25	Indoxysulfuric acid	$C_8H_7NO_4S$	213.0096	4.80	ESI-	80;132	-0.9	up
26	C14:1-carnitine	$C_{21}H_{39}NO_4$	369.2878	8.86	ESI+	60;85;311	3.2	up
27	C16:1-carnitine	$C_{23}H_{43}NO_4$	397.3191	9.68	ESI+	85;339	2.5	up
28	C6-carnitine	$C_{13}H_{25}NO_4$	259.1783	5.01	ESI+	60;144;201	-2.6	up
29	C8-carnitine	$C_{15}H_{29}NO_4$	287.2096	6.32	ESI+	60;85;127;144;229	6.4	up
30	C10-carnitine	$C_{17}H_{33}NO_4$	315.2409	7.41	ESI+	60;85;155;257	4.3	up
31	C12-carnitine*	$C_{19}H_{37}NO_4$	343.2722	8.43	ESI+	60;85;127;144;229	0.6	up
32	C14-carnitine*	$C_{21}H_{41}NO_4$	371.3035	9.38	ESI+	85;211;313	-1.4	up

*Confirmed by authentic standards. Relative metabolites were showed in Supplementary Fig. 4f.

Supplementary Table 5. Celastrol improves metabolites in mouse serum and liver in TAA-induced hepatic fibrosis.

	Metabolites	Fomula	Mass	RT (min)	Mode	Main fragments	Error (ppm)	TAA plasma	TAA liver
1	L-Phenylalanine*	$C_9H_{11}NO_2$	165.0790	1.04	ESI+	103;120;131;149	5.2	up	down
2	L-Arginine*	$C_6H_{14}N_4O_2$	174.1117	0.82	ESI+	60;70;116;130;159	-7.6	down	--
3	L-Glutamic acid*	$C_5H_9NO_4$	147.0532	0.84	ESI+	55;74;84;103;131	6.3	up	up
4	L-Tyrosine*	$C_9H_{11}NO_3$	181.0739	0.97	ESI+	119;123;136;147;165	6.1	up	--
5	L-Threonine*	$C_4H_9NO_3$	119.0582	0.84	ESI+	56;74;84;102	0.7	--	down
6	L-Serine*	$C_3H_7NO_3$	105.0426	0.84	ESI+	60;70	5.6	--	down
7	ω-Muricholic acid*	$C_{24}H_{40}O_5$	408.2876	7.68	ESI-	389;371	-5.4	up	up
8	β-Muricholic acid*	$C_{24}H_{40}O_5$	408.2876	7.95	ESI-	389;371	-0.6	up	up
9	Cholic acid*	$C_{24}H_{40}O_5$	408.2876	8.60	ESI-	389;343;325;289;251	1.2	up	up
11	Ursodeoxycholic acid/Hyodeoxycholic acid*	$C_{24}H_{40}O_4$	392.2927	8.85	ESI-	373;355	4.3	up	--
12	Deoxycholic acid*	$C_{24}H_{40}O_4$	392.2927	10.21	ESI-	373;355;345;327	4.4	up	up
13	Tauro-β/α-muricholic acid*	$C_{26}H_{45}NO_7S$	515.2916	6.34	ESI-	124;80	5.3	up	up
14	Taurocholic acid*	$C_{26}H_{45}NO_7S$	515.2916	7.20	ESI-	124;80	3.2	up	up
16	Taurochenodesoxycholic acid*	$C_{26}H_{45}NO_6S$	499.2968	8.09	ESI-	124;80	2.7	up	--
17	Taurodeoxycholic acid*	$C_{26}H_{45}NO_6S$	499.2968	8.39	ESI-	124;80	5.2	up	--
18	Acetylcarnitine*	$C_9H_{18}NO_4$	204.1236	1.03	ESI+	60;85;144;145	5.3	up	--
19	C6-carnitine	$C_{13}H_{25}NO_4$	259.1783	5.01	ESI+	60;144;201	5.2	up	--
20	C8-carnitine	$C_{15}H_{29}NO_4$	287.2096	6.32	ESI+	60;85;127;144;229	1.6	up	--
21	C10-carnitine	$C_{17}H_{33}NO_4$	315.2409	7.41	ESI+	60;85;155;257	0.4	up	up
22	C12-carnitine*	$C_{19}H_{37}NO_4$	343.2722	8.43	ESI+	60;85;127;144;229	0.5	up	up
23	C13-carnitine	$C_{20}H_{39}NO_4$	357.2878	8.76	ESI+	85;144	1.5	up	up
24	C14-carnitine*	$C_{21}H_{41}NO_4$	371.3035	9.38	ESI+	85;211;313	1.9	up	up
25	C15-carnitine	$C_{22}H_{43}NO_4$	385.3191	9.73	ESI+	144;225;328	5.4	--	up
26	C16-carnitine*	$C_{23}H_{45}NO_4$	399.3348	11.34	ESI+	85;144;239;341	5.5	--	up
27	C17-carnitine	$C_{24}H_{47}NO_4$	413.3504	10.81	ESI+	60;85;355	9.1	--	up
28	C18-carnitine*	$C_{25}H_{49}NO_4$	427.3661	11.28	ESI+	85;369	-8.3	--	up
29	C19-carnitine	$C_{26}H_{51}NO_4$	441.3817	11.64	ESI+	85;281	-5.8	--	up
30	C10-OH-carnitine	$C_{17}H_{33}NO_5$	331.2358	6.34	ESI+	85;272	2.3	up	--
31	C12-OH-carnitine	$C_{19}H_{37}NO_5$	359.2672	7.39	ESI+	60;144;301	1.5	up	up
32	C14-OH-carnitine	$C_{21}H_{41}NO_5$	387.2984	8.38	ESI+	60;85	2.5	up	up
33	C16-OH-carnitine	$C_{23}H_{45}NO_5$	415.3297	9.33	ESI+	255;357	2.9	up	up
34	C18-OH-carnitine	$C_{25}H_{49}NO_5$	443.3610	10.26	ESI+	85;385	5.3	--	up
35	C12:1-carnitine	$C_{19}H_{35}NO_4$	341.2565	7.89	ESI+	85;144;181;283	-0.3	up	up
36	C14:1-carnitine	$C_{21}H_{39}NO_4$	369.2878	8.86	ESI+	60;85;311	7.3	up	up
37	C16:1-carnitine	$C_{23}H_{43}NO_4$	397.3191	9.68	ESI+	85;339	7.5	up	up
38	C17:1-carnitine	$C_{24}H_{45}NO_4$	411.3348	10.15	ESI+	85;353	2.5	up	up
40	C19:1-carnitine	$C_{26}H_{49}NO_4$	439.3661	11.44	ESI+	86;279;382	5.7	--	up
41	C12:1-OH-carnitine	$C_{19}H_{35}NO_5$	357.2515	6.96	ESI+	60;85	4.7	up	--
42	C14:1-OH-carnitine	$C_{21}H_{39}NO_5$	385.2828	7.98	ESI+	225;327	4.8	up	up
43	C16:1-OH-carnitine	$C_{23}H_{43}NO_5$	413.3141	8.74	ESI+	85;144	4.5	up	up
44	C18:1-OH-carnitine	$C_{25}H_{47}NO_5$	441.3454	9.64	ESI+	60;85;383	5.3	up	--
45	C16:2-carnitine	$C_{23}H_{41}NO_4$	395.3035	9.16	ESI+	60;144;337	-5.2	up	--
46	LPC14:0*	$C_{22}H_{46}NO_7P$	467.3012	9.65	ESI+	86;104;184	5.3	--	up
47	LPC18:1*	$C_{26}H_{52}NO_7P$	521.3481	11.26	ESI+	86;104;184	-0.3	--	up
49	LPC20:2	$C_{28}H_{54}NO_7P$	547.3638	11.65	ESI+	86;104;184	2.4	--	up
50	LPC22:5	$C_{30}H_{52}NO_7P$	569.3481	10.71	ESI+	86;104;184	0.4	--	up
52	Pimelic acid*	$C_7H_{12}O_4$	160.0736	4.38	ESI-	114;141	5.2	--	down
53	Azelaic acid*	$C_9H_{16}O_4$	188.1049	5.82	ESI-	125;169	1.4	--	down
54	Tetradecanedioic acid*	$C_{14}H_{26}O_4$	258.1831	9.14	ESI-	195;239	-3.5	up	up
56	Hydroxybutyrate	$C_4H_8O_3$	104.0473	1.04	ESI-	59	-4.3	up	--
57	Hippurate*	$C_9H_9NO_3$	179.0582	4.85	ESI+/ESI-	77;105;134/44;77;102;134	3.5	up	--
58	Indolelactate	$C_{11}H_{11}NO_3$	205.0739	5.90	ESI+/ESI-	130;188/160	-2.2	up	--
59	Hydroxyindole	C_8H_7NO	133.0528	4.82	ESI+	117	2.5	up	--
60	Indoxylsulfuric acid	$C_8H_7NO_4S$	213.0096	4.80	ESI-	80;132	5.4	up	--
61	Phenylacetlyglycine	$C_{10}H_{11}NO_3$	193.0739	5.23	ESI+/ESI-	65;91;149/44;147	2.7	up	--
62	Uric acid	$C_5H_4N_4O_3$	168.0283	1.03	ESI-	42;96;124	2.4	up	--
63	Ethylbenzylsulfate	$C_8H_{10}O_4S$	202.0300	6.34	ESI-	80;95	7.1	up	--
64	Phenol sulphate	$C_6H_6O_4S$	173.9987	4.38	ESI-	80;93;95	8.3	up	--
65	Malic acid*	$C_4H_6O_5$	134.0215	1.02	ESI-	71;73;89;115	-0.3	up	--

66	Citric acid*	$C_6H_8O_7$	192.0270	1.03	ESI+/ESI-	111;146;174;193/57;85;87;111;129;131	4.3	up	--

*Confirmed by authentic standards. Relative metabolites were showed in Supplementray Figs. 13j and 14e.

Supplementary Table 6. Celastrol improved metabolites in culture medium.

	Metabolites	Fomula	Mass	RT (min)	Mode	Main fragments	Error (ppm)	Up/down (P.d. vs control)	Up/down (celastrol+P.d. vs P.d.)
1	Nicotinic acid	$C_6H_5NO_2$	123.0320	1.00	ESI+	53;80	1.4	up	up
2	L-Leucine*	$C_6H_{13}NO_2$	131.0946	1.01	ESI+	44;69;86	2.2	--	up
3	Arginine-Threonine	$C_{10}H_{21}N_5O_4$	275.1593	3.35	ESI+	60;70;75;120;175	1.8	up	up
4	L-Tryptophan*	$C_{11}H_{12}N_2O_2$	204.0899	1.02	ESI+	118;146;159;170;188	0.3	down	up
5	Phenylacetylglutamine	$C_{13}H_{16}N_2O_4$	264.1110	4.69	ESI+	123;152	-0.5	up	up
6	Cinnamic acid	$C_9H_8O_2$	148.0524	2.65	ESI+	77;103;131	0.5	--	up
7	Hypoxanthine	$C_5H_4N_4O$	136.0385	1.00	ESI+	55;82;94;110;119	-6.5	up	up
8	L-Methionine*	$C_5H_{11}NO_2S$	149.0510	0.99	ESI+	56;61;74	0.8	--	up
9	L-Phenylalanine*	$C_9H_{11}NO_2$	165.0790	1.02	ESI+	77;103;120	1.2	--	up
10	Indoleacrylic acid	$C_{11}H_9NO_2$	187.0633	3.60	ESI+	91;115;143	-1.3	--	up
11	L-Tyrosine*	$C_9H_{11}NO_3$	181.0739	1.00	ESI+	91;119;136	2.2	--	up
12	L-Proline*	$C_5H_9NO_2$	115.0633	0.90	ESI+	42;70	-0.6	down	up
13	Pyridoxamine 5'-phosphate	$C_8H_{13}N_2O_5P$	248.0562	3.60	ESI+	121;129;134;151;232	-2.3	up	down
14	Guanidinosuccinic acid	$C_5H_9N_3O_4$	175.0593	5.41	ESI-	86;130	-0.3	up	down

*Confirmed by authentic standards. Relative metabolites were showed in Supplementary Fig. 12g.

Supplementary Table 7. *P. distasonis* changed metabolites in culture medium.

	Metabolites	Fomula	Mass	RT (min)	Mode	Main fragments	Error (ppm)	Up/down (P.d. vs control)
1	L-Valine*	$C_5H_{11}NO_2$	117.0790	0.95	ESI+	55;72	2.1	down
2	Adenosine	$C_{10}H_{13}N_5O_4$	267.0967	0.93	ESI+	136	1.3	down
3	Leucine-Leucine	$C_{12}H_{24}N_2O_3$	244.1787	4.39	ESI+	86;132	1.5	down
4	Leucine-Valine	$C_{11}H_{22}N_2O_3$	230.1630	1.00	ESI+	86;118	-0.4	down
5	Phenylalanine-Phenylalanine	$C_{18}H_{20}N_2O_3$	312.1474	4.97	ESI+	120;166	1.5	down
6	Leucine-Glycine	$C_8H_{16}N_2O_3$	188.1161	1.01	ESI+	76;86;132	-1.5	down
7	Threonine-Leucine	$C_{10}H_{20}N_2O_4$	232.1423	1.00	ESI+	74;120;132	-5.4	down
8	Tyrosine-Leucine	$C_{15}H_{22}N_2O_4$	294.1579	4.05	ESI+	136	-3.2	down
9	Phenylalanine-Alanine	$C_{12}H_{16}N_2O_3$	236.1161	3.53	ESI+	90;120;166	1.4	down
10	L-Glutamic acid*	$C_5H_9NO_4$	147.0532	0.89	ESI+	84;102;113;130	-5.6	down
11	L-Glutamine*	$C_5H_{10}N_2O_3$	146.0691	0.97	ESI+	56;84;102;130	-3.5	down
12	Valine-Arginine	$C_{11}H_{23}N_5O_3$	273.1801	0.88	ESI+	72;118;175	1.6	down
13	Histidine-Proline	$C_{11}H_{16}N_4O_3$	252.1222	3.27	ESI+	110;156	7.5	down
14	L-Alanine*	$C_3H_7NO_2$	89.0477	0.89	ESI+	44;63	3.5	down
15	Leucine-Methionine	$C_{11}H_{22}N_2O_3S$	262.1351	1.01	ESI+	86;132;150	6.4	down
16	Leucine-Tryptophan	$C_{17}H_{23}N_3O_3$	317.1739	4.73	ESI+	86;132;205	5.4	down
17	Leucine-Serine	$C_9H_{18}N_2O_4$	218.1267	2.43	ESI+	86;132	2.3	down
18	L-Leucine*	$C_6H_{13}NO_2$	131.0946	1.01	ESI+	44;69;86	6.5	down
19	Leucine-Phenylalanine	$C_{15}H_{22}N_2O_3$	278.1630	4.77	ESI+	86;120;132;166	-2.4	down
20	L-Arginine*	$C_6H_{14}N_4O_2$	174.1117	0.98	ESI+	60;70;98;116;130	1.5	down
21	Adipoylcarnitine	$C_{13}H_{23}NO_6$	289.1525	1.01	ESI+	85;129;231	9.4	down
22	L-Proline*	$C_5H_9NO_2$	115.0633	0.90	ESI+	70	5.7	down
23	Proline-Alanine	$C_8H_{14}N_2O_3$	186.1004	0.93	ESI+	70;44	2.6	down
24	LPC16:0*	$C_{24}H_{50}NO_7P$	495.3325	11.02	ESI+	104;184;257;478	8.9	down
25	Leucine-Proline	$C_{11}H_{20}N_2O_3$	228.1474	1.01	ESI+	70;86;116;132	5.7	down
26	Tyramine	$C_8H_{11}NO$	137.0841	1.00	ESI+	77;93;103;121	3.5	down
27	Glycine-Phenylalanine	$C_{11}H_{14}N_2O_3$	222.1004	3.46	ESI-	164;74	4.3	down
28	Leucine-Alanine	$C_9H_{18}N_2O_3$	202.1317	1.02	ESI-	87;131	-3.1	down
29	Adenosine 2',3'-cyclic phosphate	$C_{10}H_{12}N_5O_6P$	329.0525	1.02	ESI-	107;133;192	-0.6	down
30	Cytidine 2',3'-cyclic phosphate	$C_9H_{12}N_3O_7P$	305.0413	0.93	ESI-	110	-2.6	down
31	L-Galactose	$C_6H_{12}O_6$	180.0634	0.88	ESI-	143;161	-2.3	down
32	L-Serine*	$C_3H_7NO_3$	105.0426	0.87	ESI-	74	1.0	down
33	Guanosine 2',3'-cyclic phosphate	$C_{10}H_{12}N_5O_7P$	345.0474	1.02	ESI-	133;150	4.2	down
34	Ribonic acid	$C_5H_{10}O_6$	166.0477	0.88	ESI-	43;121	-3.2	down
35	Glyceric acid	$C_3H_6O_4$	106.0266	0.91	ESI-	45;59	2.5	down
36	L-Phenylalanine*	$C_9H_{11}NO_2$	165.079	1.04	ESI-	72;92;103;147	2.3	down
37	N-Undecylbenzenesulfonic acid	$C_{17}H_{28}O_3S$	312.1759	12.54	ESI-	80;231	1.3	down
38	Citrulline	$C_6H_{13}N_3O_3$	175.0957	0.88	ESI-	131	2.4	down
39	LPE20:4	$C_{25}H_{44}NO_7P$	501.2855	10.86	ESI+	362;441	3.4	up
40	LPE18:1	$C_{23}H_{46}NO_7P$	479.3012	10.86	ESI+	339;419	2.2	up
41	Propylthiouracil	$C_7H_{10}N_2OS$	170.0514	4.81	ESI+	142;154	-0.3	up
42	Trimethylamine	C_3H_9N	59.0735	0.83	ESI+	44	-0.4	up
43	N-acetylleucine	$C_8H_{15}NO_3$	173.1052	4.97	ESI+	114;128;132;157	0.5	up
44	LPE16:0*	$C_{21}H_{44}NO_7P$	453.2855	10.78	ESI+	313;393	-0.6	up
45	LPE16:1	$C_{21}H_{42}NO_7P$	451.2699	9.72	ESI+	311;434	1.5	up
46	Guanine	$C_5H_5N_5O$	151.0494	1.00	ESI+	109;110;135	2.4	up
47	Nicotinic acid	$C_6H_5NO_2$	123.0320	1.00	ESI+	52;53;78	2.1	up
48	Cadaverine	$C_5H_{14}N_2$	102.1157	0.78	ESI+	69;86	0.5	up
49	L-Lactic acid*	$C_3H_6O_3$	90.0317	0.81	ESI-	43;59;71	2.2	up
50	Lactoylleucine	$C_9H_{17}NO_4$	203.1158	5.13	ESI-	116;158	-2.3	up
51	Xanthosine	$C_{10}H_{12}N_4O_6$	284.0757	1.02	ESI-	108;151	-5.6	up
52	N-Acetyl-L-methionine	$C_7H_{13}NO_3S$	191.0616	3.95	ESI-	98;142	-0.5	up
53	N-Acetyl-L-phenylalanine	$C_{11}H_{13}NO_3$	207.0895	5.28	ESI-	91;147;165	1.3	up
54	2-Hydroxybutyric acid	$C_4H_8O_3$	104.0473	1.03	ESI-	46;58	1.5	up
55	Xanthine	$C_5H_4N_4O_2$	152.0334	1.01	ESI-	108	5.3	up
56	Uric acid	$C_5H_4N_4O_3$	168.0283	1.01	ESI-	83;124	2.1	up
57	Guanidinosuccinic acid	$C_5H_9N_3O_4$	175.0593	5.41	ESI-	86;130	3.6	up
58	Hypoxanthine	$C_5H_4N_4O$	136.0385	1.01	ESI-	65;92	3.8	up
59	Succinic acid*	$C_4H_6O_4$	118.0266	1.52	ESI-	56;73	-2.3	up
60	Uracil	$C_4H_4N_2O_2$	112.0273	1.01	ESI-	42;67	-2.3	up
61	2-Hydroxy-4-(methylthio)butanoic acid	$C_5H_{10}O_3S$	150.0351	3.64	ESI-	105	-2.0	up
62	Indolelactic acid*	$C_{11}H_{11}NO_3$	205.0739	5.50	ESI-	160	1.0	up
63	Methionine-Glutamic acid	$C_{10}H_{18}N_2O_5S$	278.0936	1.02	ESI-	148;151	1.7	up

*Confirmed by authentic standards. Relative metabolites were showed in Supplementary Fig. 6b.

Supplementary Table 8. *P. distasonis* improved serum metabolites in mice in MCD diet-induced hepatic fibrosis.

	Metabolites	Fomula	Mass	RT (min)	Mode	Main fragments	Error (ppm)	Up/down (MCD vs MCS)
1	L-Serine*	$C_3H_7NO_3$	105.0426	0.91	ESI+	60;70	-0.2	up
2	Indoleacrylic acid*	$C_{11}H_9NO_2$	187.0633	3.47	ESI+	115;118;144;170	2.5	up
3	4-Trimethylammoniobutanoic acid	$C_7H_{15}NO_2$	145.1103	1.03	ESI+	60;87	-2.9	up
4	12-Ketodeoxycholic acid	$C_{24}H_{38}O_4$	390.2770	8.05	ESI+	355;373	1.3	up
5	L-Threonine*	$C_4H_9NO_3$	119.0582	0.89	ESI+	56;74;84	5.3	up
6	L-Glutamine*	$C_5H_{10}N_2O_3$	146.0691	0.89	ESI+	56;84	-1.0	up
7	Pyridoxamine 5'-phosphate	$C_8H_{13}N_2O_5P$	248.0562	3.60	ESI+	121;129;134;151;232	0.2	up
8	Hexanoylglycine	$C_8H_{15}NO_3$	173.1052	5.29	ESI-	74	3.7	down
9	Taurochenodesoxycholic acid*	$C_{26}H_{45}NO_6S$	499.7046	8.09	ESI-	80;124	-0.6	up
10	ω-Muricholic acid*	$C_{24}H_{40}O_5$	408.2876	7.68	ESI-	371;389	7.1	up

*Confirmed by authentic standards. Relative metabolites were showed in Supplementray Fig. 17b.

Supplementary Table 9. Celastrol improved metabolites in mice serum and liver in MCD diet-induced hepatic fibrosis.

	Metabolites	Fomula	Mass	RT (min)	Mode	Main fragments	Error (ppm)	MCD plasma	MCD liver
1	L-Glutamic acid*	$C_5H_9NO_4$	147.0532	0.84	ESI+	55;74;84;103;131	6.3	--	up
2	L-Threonine*	$C_4H_9NO_3$	119.0582	0.84	ESI+	56;74;84;102	0.7	--	up
3	L-Serine*	$C_3H_7NO_3$	105.0426	0.84	ESI+	60;70	5.6	--	up
4	ω-Muricholic acid*	$C_{24}H_{40}O_5$	408.2876	7.68	ESI-	389;371	-5.4	up	up
5	β-Muricholic acid*	$C_{24}H_{40}O_5$	408.2876	7.95	ESI-	389;371	-0.6		up
6	Cholic acid*	$C_{24}H_{40}O_5$	408.2876	8.60	ESI-	389;343;325;289;251	1.2	up	
7	Lithocholic acid*	$C_{24}H_{40}O_3$	376.2977	12.03	ESI-	339;357	1.4	--	up
8	Tauro-β/α-muricholic acid*	$C_{26}H_{45}NO_7S$	515.2916	6.34	ESI-	124;80	5.3	up	up
9	Taurocholic acid*	$C_{26}H_{45}NO_7S$	515.2916	7.20	ESI-	124;80	3.2	up	up
10	Taurochenodesoxycholic acid*	$C_{26}H_{45}NO_6S$	499.2968	8.09	ESI-	124;80	2.7	up	--
11	Taurodeoxycholic acid*	$C_{26}H_{45}NO_6S$	499.2968	8.39	ESI-	124;80	5.2	up	--
12	C16-OH-carnitine	$C_{23}H_{45}NO_5$	415.3297	9.33	ESI+	255;357	2.9	up	--
13	C18:1-carnitine	$C_{25}H_{47}NO_4$	425.3504	10.594	ESI+	85;265;367	6.1	--	up
14	LPC20:1	$C_{28}H_{56}NO_7P$	549.3794	12.716	ESI+	86;105;184	7.3	down	--
15	LPE18:0*	$C_{23}H_{48}NO_7P$	481.3168	12.149	ESI+	341;421	8.4	down	--
16	Tetradecanedioic acid*	$C_{14}H_{26}O_4$	258.1831	9.14	ESI-	195;239	-3.5	up	--
17	Prpionate*	$C_3H_6O_2$	74.0368	1.059	ESI-	58	8.1	down	--
18	Indolelactate	$C_{11}H_{11}NO_3$	205.0739	5.90	ESI+/ESI-	130;188/160	-2.2	down	--
19	Uric acid	$C_5H_4N_4O_3$	168.0283	1.03	ESI-	42;96;124	2.4	down	--
20	Phenol sulphate	$C_6H_6O_4S$	173.9987	4.38	ESI-	80;93;95	8.3	down	--

*Confirmed by authentic standards. Relative metabolites were showed in Supplementray Fig. 18h.

Supplementary Table 10. Primer sequences for QPCR.

**Mouse primers**	**Sequence**	**Human primers**	**Sequence**
Collagen1a1	CATGTTCAGCTTTGTGGACCT	H-Caspase-4	CAAGAGAAGCAACGTATGGCA
	GCAGCTGACTTCAGGGATGT		AGGCAGATGGTCAAACTCTGTA
Timp1	GCAAAGAGCTTTCTCAAAGACC	H-Caspase-3	CATGGAAGCGAATCAATGGACT
	AGGGATAGATAAACAGGGAAACACT		CTGTACCAGACCGAGATGTCA
αSMA	GTCCCAGACATCAGGGAGTAA	H-Collagen1a1	GAGGGCCAAGACGAAGACATC
	TCGGATACTTCAGCGTCAGGA		CAGATCACGTCATCGCACAAC
Tgfb	GGAGAGCCCTGGATACCAAC	H-Timp1	CTTCTGCAATTCCGACCTCGT
	CAACCCAGGTCCTTCCTAAA		ACGCTGGTATAAGGTGGTCTG
Il1b	CCCTGCAGCTGGAGAGTGTGGA	H-αSMA	CGTGGCTATTCCTTCGTTAC
	TGTGCTCTGCTTGTGAGGTGCTG		TGCCAGCAGACTCCATCC
Il6	CGGAGAGGAGACTTCACAGAGGA	H-Tgfb	CGCAAGGACCTCGGCTGGAAGTG
	TTTCCACGATTTCCCAGAGAACA		GCGCCCGGGTTATGCTGGTTGTA
Tnfa	CCACCACGCTCTTCTGTCTAC	H-Il1b	ATGATGGCTTATTACAGTGGCAA
	AGGGTCTGGGCCATAGAACT		GTCGGAGATTCGTAGCTGGA
Caspase11	ACAAACACCCTGACAAACCAC	H-Il6	ACTCACCTCTTCAGAACGAATTG
	CACTGCGTTCAGCATTGTTAAA		CCATCTTTGGAAGGTTCAGGTTG
Caspase3	AACGGACCTGTGGACCTGAA	H-Tnfa	GGTTTAGAAGATTTTTTTCGGAATC
	TCAATACCGCAGTCCAGCTC		TAAACCCTACACCTTCTATCTCGAT
Fxr	TGGGCTCCGAATCCTCTTAGA	H-Fxr	AACCATACTCGCAATACAGCAA
	TGGTCCTCAAATAAGATCCTTGG		ACAGCTCATCCCCTTTGATCC
Shp	TCTGCAGGTCGTCCGACTATTC	H-Fgf19	CGGAGGAAGACTGTGCTTTCG
	AGGCAGTGGCTGTGAGATGC		CTCGGATCGGTACACATTGTAG
Cyp7a1	GGGAATGCCATTTACTTGGA	H-Ostb	TCCAGGCAAGCAGAAAAGAAA
	GTCCGGATATTCAAGGATGC		ACTGACAGCACATCTCTCTCT
Cyp8b1	TCCTCAGGGTGGTACAGGAG	H-Gapdh	GCACCGTCAAGGCTGAGAAC
	GATAGGGGAAGAGAGCCACC		TGGTGAAGACGCCAGTGGA
Ntcp	AGGGGGACATGAACCTCAG		
	TCCGTCGTAGATTCCTTTGC	**Bacterial Primers**	**Sequence**
Oatp1	ACTCCCATAATGCCCTTGG	*Bacteroidetes*	GAGAGGAAGGTCCCCCAC
	TAATCGGGCCAACAATCTTC		CGCTACTTGGCTGGTTCAG
Oatp4	ACCAAACTCAGCATCCAAGC	*Firmicutes*	GGAGYATGTGGTTTAATTCGAAGCA
	TAGCTGAATGAGAGGGCTGC		AGCTGACGACAACCATGCAC
Ostb	GTATTTTCGTGCAGAAGATGCG	*Proteobacteria*	TCGTCAGCTCGTGTYGTGA
	TTTCTGTTTGCCAGGATGCTC		CGTAAGGGCCATGATG
Mrp3	CTGGGTCCCCTGCATCTAC	*P. distasonis*	GGACACGTCCCGCACTTTAT
	GCCGTCTTGAGCCTGGATAAC		TTCTGAGAGGAAGGTCCCCC
Mrp4	AGCTTCAACGGTACTGGGATA	*Eubacteria*	ACTCCTACGGGAGGCAGCAG
	TCGTCGGGGTCATACTTCTC		ATTACCGCGGCTGCTGG
Bsep	CCAGAACATGACAAACGGAA		
	AAGGACAGCCACACCAACTC		
Mrp2	TCCAGGACCAAGAGATTTGC		
	TCTGTGAGTGCAAGAGACAGGT		
Fgf15	ATGGCGAGAAAGTGGAACGG		
	CTGACACAGACTGGGATTGCT		
Ibabp	CCCCAACTATCACCAGACTTC		
	ACATCCCCGATGGTGGAGAT		
Osta	CACTGGCTCAGTTGCCATTT		
	GCATACGGCATAAAACGAGGT		
Mt-nd6	CACAACTATATATTGCCGCTACC		
	GCTACTGAGGAATATCCAGAGAC		
Sdha	TGCTGGAGAAGAATCGGTTATG		
	ACAGCATCAGATTCTGCAGCTCC		
Sdhb	AATTTGCCATTTACCGATGGGA		
	AGCATCCAACACCATAGGTCC		
Uqcrc1	AGACCCAGGTCAGCATCTTG		
	GCCGATTCTTTGTTCCCTTGA		
Mt-co1	GTCTGATCCGTACTTATTACAG		
	GCTCATACTATTCCTATATAGCCG		
Atp5a	GAGACTGGGCGTGTGTTAAGCA		
	CATTACCGAGGGCGTCAACCAC		
Nrf1	CAGACGACGCAAGCATCAGAG		
	GCTCCGACGGCTGCTGCGGTTTC		
Tfam	ATTCCGAAGTGTTTTTCCAGCA		
	TCTGAAAGTTTTGCATCTGGGT		
18S	ATTACCGCGGCTGCTGGC		
	CGGCTACCACATCCAAGGAA		
Gpx4	ACGATGCCCACCCACT		
	CCACGCAGCCGTTCTT		
Cox2	TGACCCCCAAGGCTCAAATAT		
	TGAACCCAGGTCCTCGCTTA		

Supplementary Figures

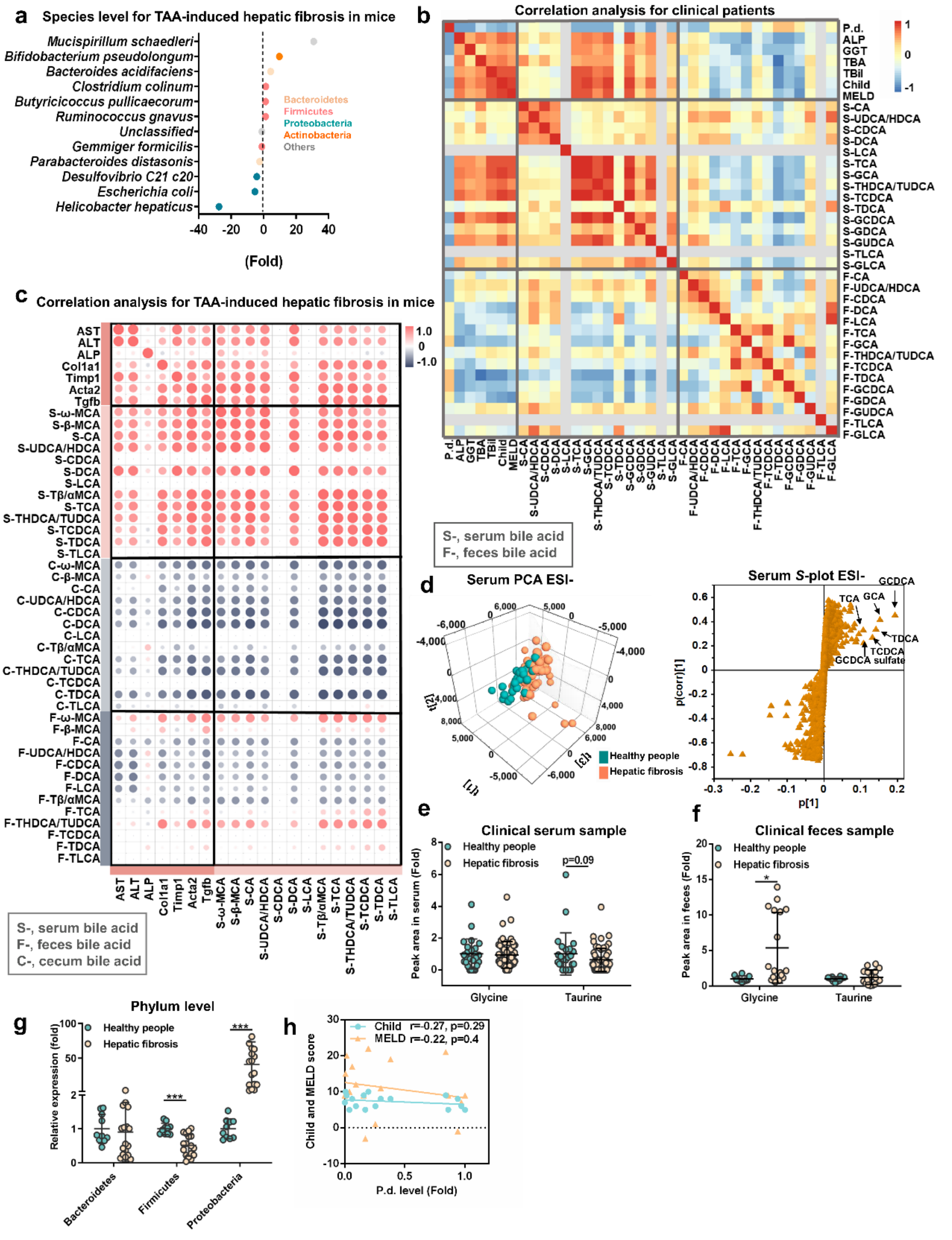

Supplementary Fig. 1 Hepatic fibrosis decreased *P. distasonis* level and BSH activity. a Fold change for species level in TAA-induced hepatic fibrosis in mice (n=5). Different phylum was showed with different color, negative number showed the decreased fold in TAA group

and positive number showed the increased fold. Mice were treated with 200 mg/kg TAA for 6 weeks. **b** Correlation analysis between serum bile acid (marked by "S-"), feces bile acid (marked by "F-"), hepatic fibrosis indexes (ALP, GGT, TBA, TBil, Child and MELD scores), and *P. distasonis* level in healthy people (n=10) and hepatic fibrosis patients (n=10). Red color showed the positive correlation and blue color showed the negative correlation. Serum bile acids were negatively correlated with feces bile acids indicating the inhibited excretion of bile acids in the intestinal tract. *P. distasonis* levels were negatively correlated with hepatic fibrosis indexes (e.g., Child and MELD scores). P.d., *P. distasonis*; GGT, gamma-glutamyl transpeptidase; TBA, total bile acid; TBil, total bilirubin. **c** Correlation analysis between serum bile acid (marked by "S-"), cecum content bile acid (marked by "C-"), feces bile acid (marked by "F-"), and hepatic fibrosis indexes (AST, ALT, ALP, *Col1a1*, *Timp1*, *Acta2* and *Tgfb* genes) in TAA-induced hepatic fibrosis in mice (n=6). Mice were treated as in **a**. Red color shows the positive correlation and blue color shows the negative correlation. Serum bile acids were negatively correlated with cecum content and feces bile acids implying the inhibited excretion of bile acids in intestinal tract. **d** Principal component analysis (PCA) score plot (left) and *S*-plot (right) for the serum metabolome detected in ESI-. Serum samples were collected from healthy people (n=25) and hepatic fibrosis patients (n=62). Conjugated bile acids (e.g., TCDCA and GCDCA) were increased in serum indicating the decreased BSH activity. **e** Serum glycine and taurine levels in healthy people (n=25) and hepatic fibrosis patients (n=62). Taurine was decreased in serum implying the decreased BSH activity. **f** Feces glycine and taurine levels in healthy people (n=10) and hepatic fibrosis patients (n=17). **g** *Bacteroidetes* (phylum), *Firmicutes* (phylum), and *Proteobacteria* (phylum) level in healthy people (n=10) and hepatic fibrosis patients (n=17). *P<0.05, ***P<0.001. **h** Correlation analysis between *P. distasonis* level and Child and MELD scores in hepatic fibrosis patients (n=17). Data are presented as the mean ± SD.

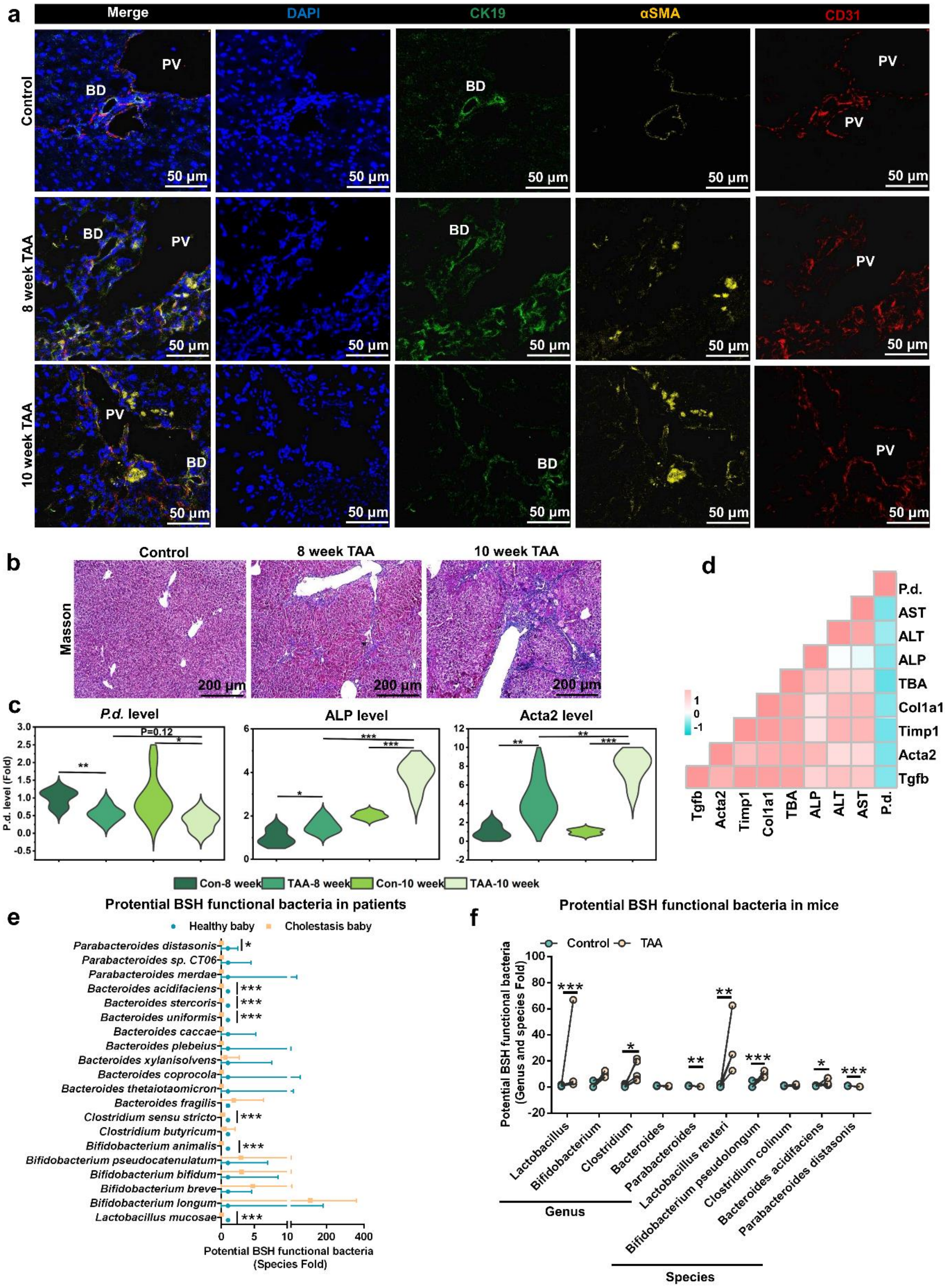

Supplementary Fig. 2 ***P. distasonis*** **levels were negatively correlated with hepatic fibrosis indexes in mice, and BSH functional bacteria are summarized in patients and mice. a** Hepatic immunofluorescent staining for DAPI (marked cell nucleus), CK19 (marked bile duct), CK31 (marked blood vessel) and αSMA (marked hepatic fibrosis). Mice were treated with 200

mg/kg TAA for 8 and 10 weeks. PV, portal vein; BD, bile duct. **b** Hepatic Masson trichrome staining in 8 and 10 week TAA-induced hepatic fibrosis. **c** *P. distasonis* levels, ALP levels, and *Acta2* mRNA expression were increased with the increased TAA administration time in mice (n=6). Mice were treated as in **a**. P.d., *P. distasonis*. For violin plot, violin represents kernel density estimation. **d** *P. distasonis* levels were negatively correlated with all the hepatic fibrosis indexes (AST, ALT, ALP, TBA, *Col1a1*, *Timp1*, *Acta2* and *Tgfb* mRNAs). Red color shows the positive correlation and green color shows the negative correlation. TBA, total bile acid. Mice were treated as in **a**. n=6 per group. **e** Potential BSH functional bacteria including *Bacteroidetes* (phylum), *Lactobacillus* (genus), *Bifidobacterium* (genus) and *Clostridium* (genus) were summarized in healthy infants (n=12) and cholestatic infants (n=13). *P. distasonis* levels were dramatically decreased in clinical liver injury patients. **f** Potential BSH functional bacteria including *Lactobacillus* (genus), *Bifidobacterium* (genus), *Clostridium* (genus), *Bacteroides* (genus), and *Parabacteroides* (genus) are summarized in 6 week 200 mg/kg TAA-induced hepatic fibrosis in mice (n=5). *P. distasonis* levels were decreased in hepatic fibrosis mice. Data are presented as the mean ± SD. **P*<0.05, ***P*<0.01, ****P*<0.001.

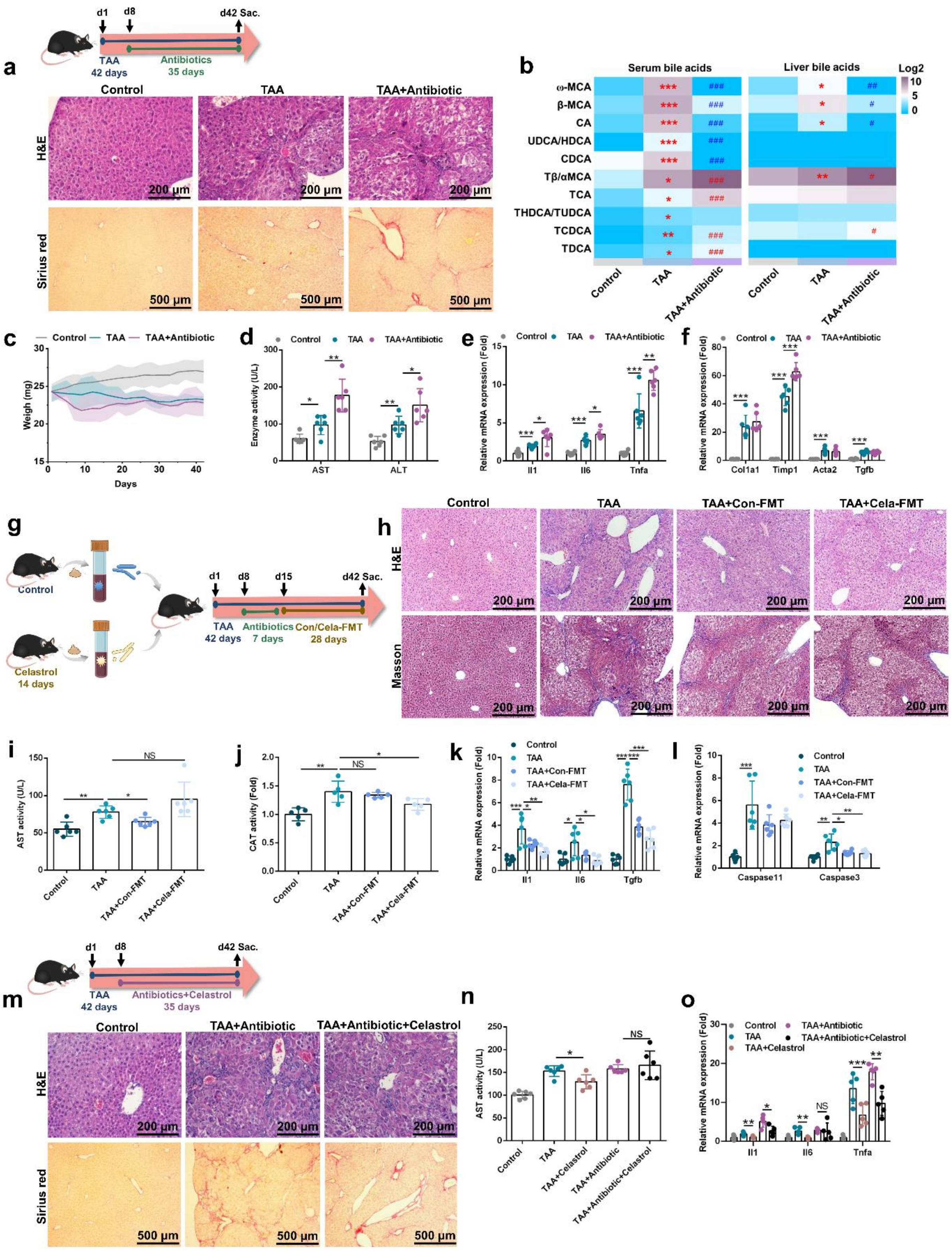

Supplementary Fig. 3 Gut microbiota participate in the development of hepatic fibrosis and the protective effects of celastrol in mice. a Experimental scheme and tissue section staining (H&E and Sirius red) after antibiotics treatment. Experimental scheme: mice were treated with 200 mg/kg TAA for 6 weeks; after TAA treatment for 1 week, mice were treated with antibiotics (ampicillin, neomycin, metronidazole, and vancomycin) for 5 weeks (n=6 biologically independent animals). **b** Serum and hepatic bile acid levels after TAA and

antibiotics treatments. Purple color shows the higher bile acid levels and blue color shows the lower bile acid levels. Heatmap plots were generated by log2 transformation of data. Mice were treated as in **a**. *P<0.05, **P<0.01, ***P<0.001 verse Control group; #P<0.05, ##P<0.01, ###P<0.001 verse TAA group. **c** Body weight changes after TAA and antibiotics treatment. Mice were treated as in **a**. **d** Serum AST and ALT enzyme activities after antibiotics treatment. Mice were treated as in **a** (n=6). **e-f** Proinflammatory factor (**e**) and hepatic fibrosis (**f**) mRNA expression in liver after TAA and antibiotic treatment. Mice were treated as in **a** (n=6). **g** Experimental scheme for fecal microbial transplantation (FMT): mice were treated with 200 mg/kg TAA for 6 weeks; after TAA treatment for 1 week, mice were treated with antibiotics (ampicillin, neomycin, metronidazole, vancomycin) for 1 week; after antibiotics treatment, fecal microbiota transplant (FMT) was conducted for 4 weeks (n=6). The donor mice in the TAA+Con-FMT group were healthy mice. The donor mice in the TAA+Cela-FMT group were 2 week 10 mg/kg celastrol-treated mice. **h** H&E and Masson trichrome staining for FMT. Mice were treated as in **g**. **i-j** Serum AST enzyme activity (**i**, n=6) and liver catalase (CAT) level (**j**, n=5) for FMT. Mice were treated as in **g**. **k** Proinflammatory factor (*Il1* and *Il6*) and hepatic fibrosis (*Tgfb*) mRNA expression in liver for FMT. Mice were treated as in **g** (n=6). **l** *Caspase-11* pyroptosis mRNA expression in liver for FMT. Mice were treated as in **g** (n=6). **m** Experimental scheme and tissue section staining (H&E and Sirius red) after TAA, antibiotics and celastrol co-treatments. Experimental scheme: mice were treated with 200 mg/kg TAA for 6 weeks; after TAA treatment for 1 week, mice were treated with antibiotics (ampicillin, neomycin, metronidazole, and vancomycin) and 10 mg/kg celastrol for 5 weeks (n=6). **n** Serum AST enzyme activity after TAA, antibiotics and celastrol co-treatments. Mice were treated as in **m**. **o** Proinflammatory factor gene expression in liver after TAA, antibiotics and celastrol co-treatments. Mice were treated as in **m**. Data are presented as the mean ± SD. *P<0.05, **P<0.01, ***P<0.001.

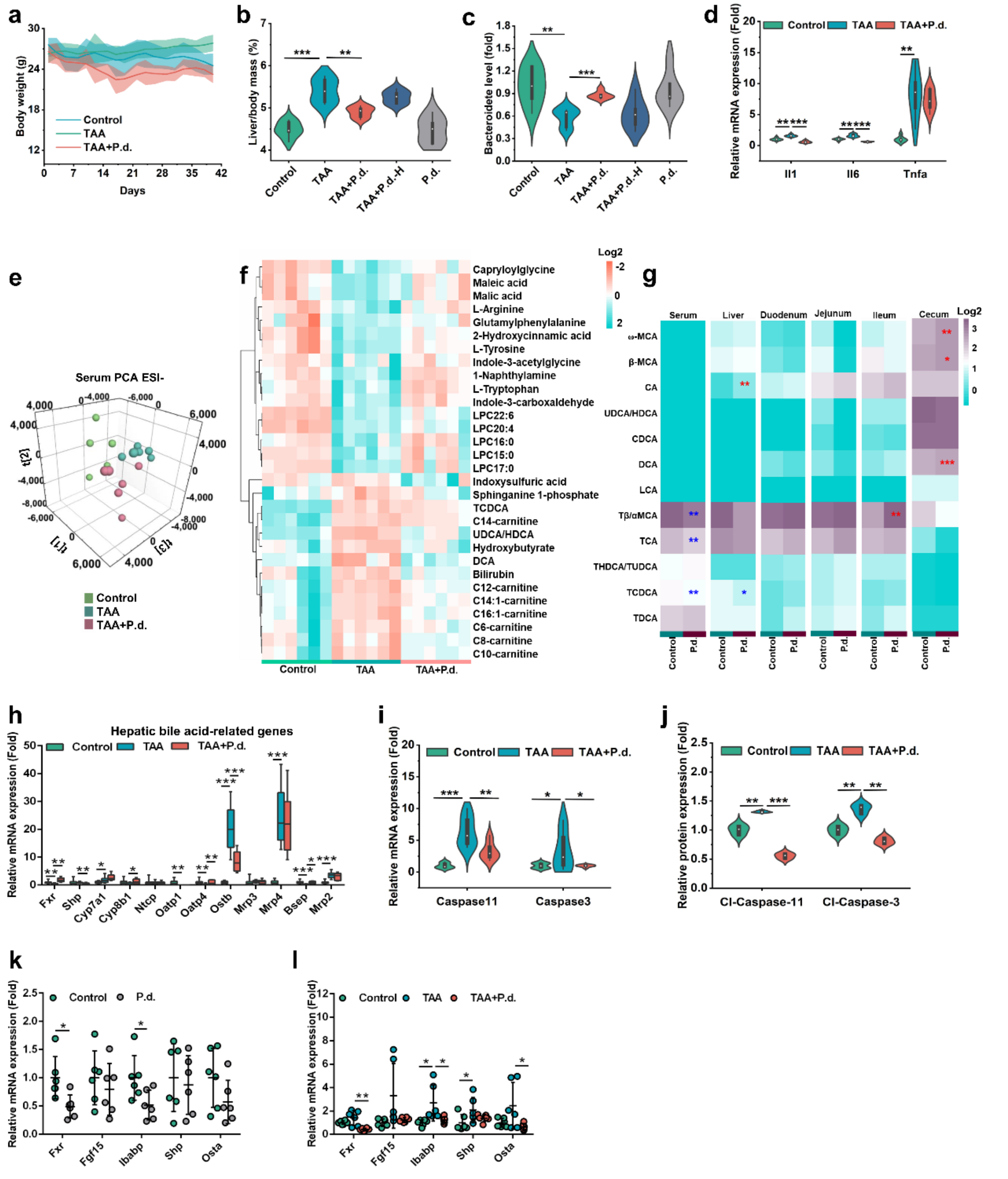

Supplementary Fig. 4 *P. distasonis* improves TAA-induced hepatic fibrosis in mice. Mice were treated with 200 mg/kg TAA for 6 weeks. After TAA treatment for 1 week, the mice were treated with antibiotics (ampicillin, neomycin, metronidazole and vancomycin) for 1 week. After antibiotics treatment, *P. distasonis* (P.d., 2×10^{8} CFU) and heat-killed *P. distasonis* (P.d.-H) were given by oral transplantation once a day for 4 weeks (n=6 biologically independent animals). **a** Body weight. **b** Liver/body mass. **c** *Bacteroidetes* (phylum) level in cecum content. **d** Proinflammatory factor gene expression in liver. **e** Principal component analysis (PCA) score plot for the serum metabolome detected in ESI-. Each point represented a sample. **f** Heatmap of 30 significantly changed endogenous metabolites after *P. distasonis* treatment in serum

using non-target metabolomics. Red color showes higher metabolite levels and green color shows lower metabolite levels. Heatmap plots were generated by log2 transformation of data. TCDCA, taurochenodeoxycholic acid; UDCA, ursodeoxycholic acid; HDCA, hyodeoxycholic acid; DCA, deoxycholic acid. **g** *P. distasonis* improved bile acid levels in enterohepatic circulation (serum, liver, duodenum, jejunum, ileum and cecum content) in healthy mice. Healthy mice were treated with *P. distasonis* (2×10^8 CFU) for 4 weeks. Purple color shows higher bile acid levels and green color shows lower bile acid levels. Heatmap plots were generated by log2 transformation of data. n=6 per group. **h** Hepatic mRNA expression of *Fxr*, *Shp,* bile acid syntheses (*Cyp7a1* and *Cyp8b1*), basolateral uptake transporters (*Ntcp*, *Oatp1* and *Oatp4*), basolateral efflux transporters (*Ostβ*, *Mrp3* and *Mrp4*) and canalicular transporters (*Bsep* and *Mrp2*). **i** Caspase-11 pyroptosis mRNA expression in liver. In box plot, the center line indicates the median, the edges of the box represent the first and third quartiles, and the whiskers extend to span a 1.5 interquartile range from the edges. **j** Quantitative analysis of Caspase-11 pyroptosis protein expression in liver. Cl-Caspase-11/3 are the active form of the protein. Western blot images are shown in Fig. 2g. **k** Ileal FXR target gene expression after 4 week 2×10^8 CFU *P. distasonis* treatment in healthy mice (n=6). **l** Ileal FXR target gene expression after *P. distasonis* treatment in TAA-induced hepatic fibrosis. Data are presented as the mean ± SD. **P*<0.05, ***P*<0.01, ****P*<0.001. For violin plot (**b**-**d**, **i**-**j**), boxplots represent median with the interquartile range, whiskers indicate adjacent values, violin represents kernel density estimation.

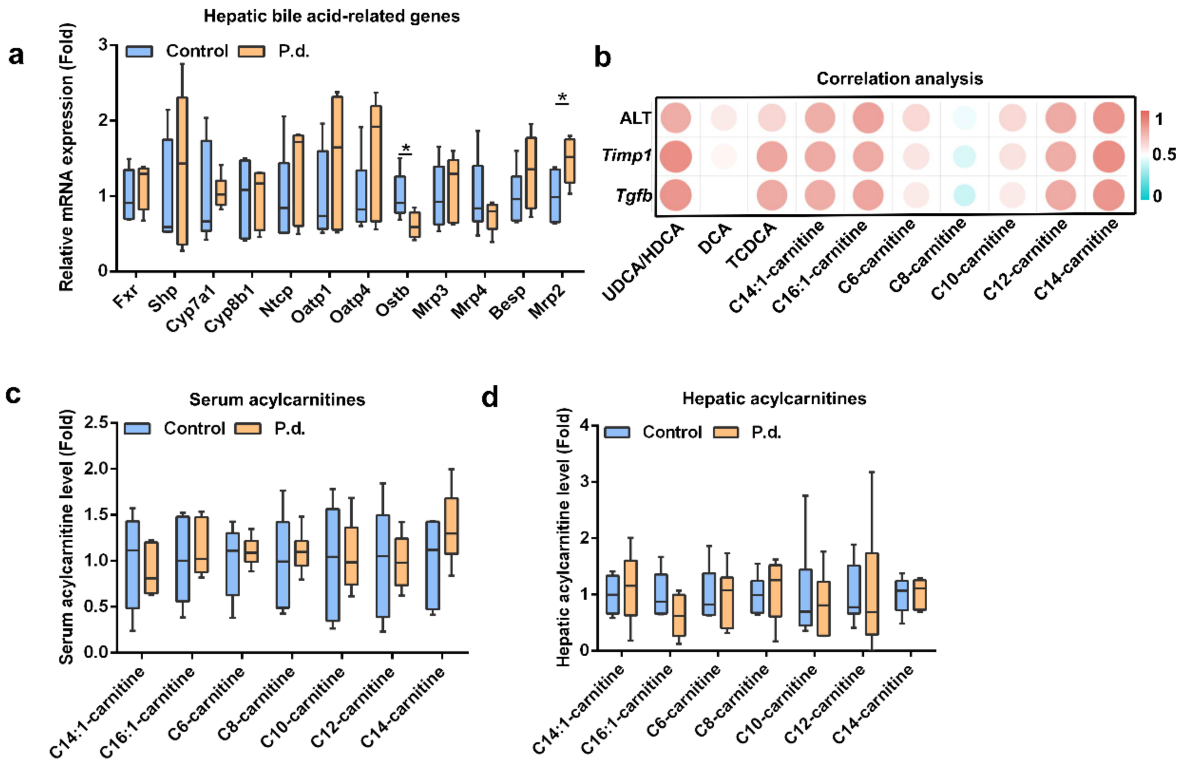

Supplementary Fig. 5 The acylcarnitine pathway is not influenced by *P. distasonis* in healthy mice. a Hepatic bile acid gene expression after 4 week 2×10^8 CFU *P. distasonis* treatment in healthy mice (n=6). **b** Serum bile acids and acylcarnitines were positively correlated with hepatic fibrosis indexes (ALT, *Timp1* and *Tgfb* mRNA) in TAA-induced hepatic fibrosis. Red color showed the positive correlation. Mice were treated as in Supplementary Fig. 4. n=6 per group. **c-d** Serum acylcarnitines (**c**) and hepatic acylcarnitines (**d**) were not influenced by 4 week 2×10^8 CFU *P. distasonis* treatment in healthy mice (n=6). **P*<0.05. In box plot (**a**, **c-d**), the center line indicates the median, the edges of the box represent the first and third quartiles, and the whiskers extend to span a 1.5 interquartile range from the edges.

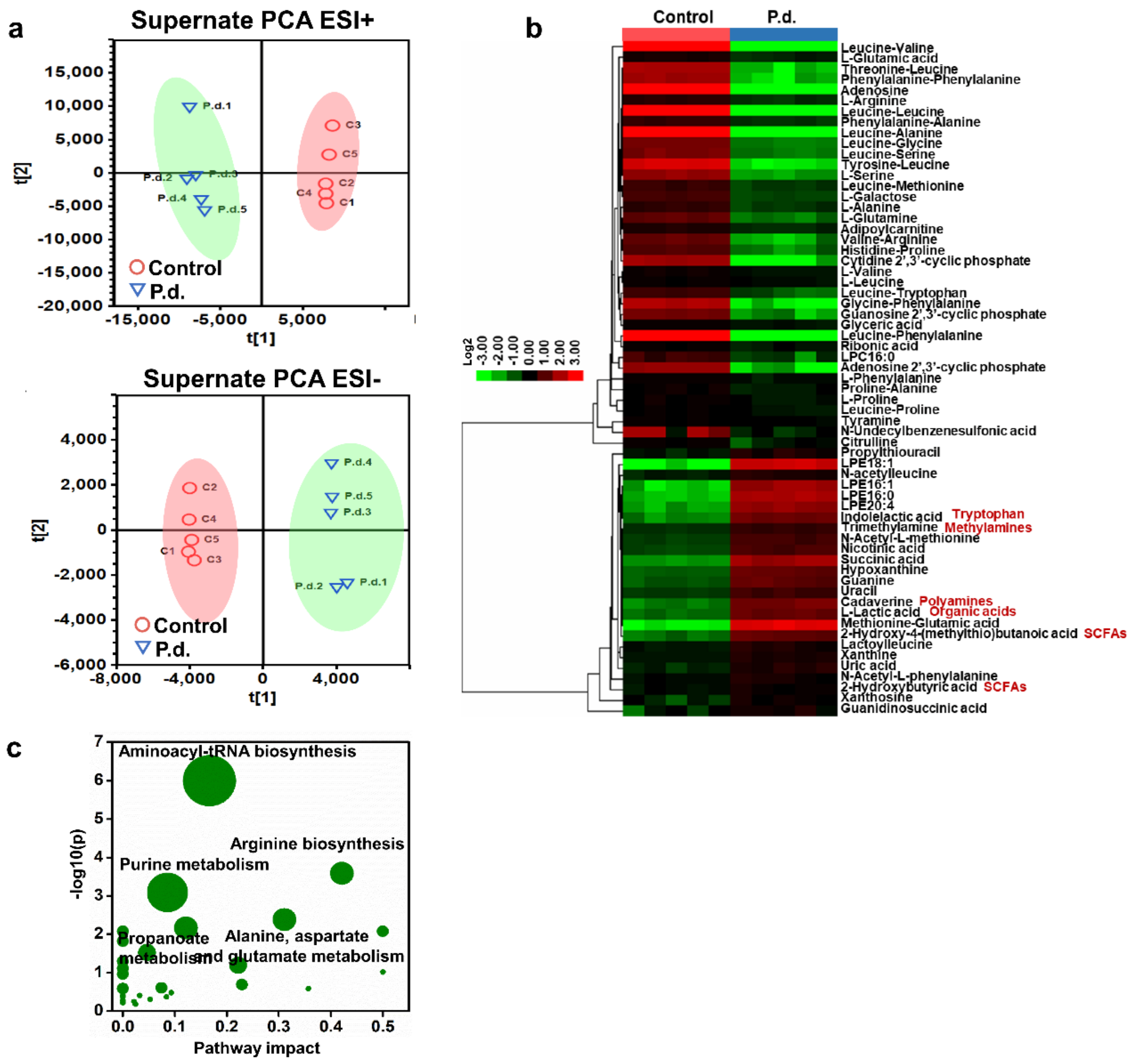

Supplementary Fig. 6 *P. distasonis* influences various metabolites in brain-heart infusion fluid medium. a Principal component analysis (PCA) score plot for the culture medium metabolome detected in ESI+ and ESI-. Each point represent a sample. The original concentration of *P. distasonis* was 22×10^5 CFU/mL. *P. distasonis* was cultivated for 24 h (n=5). **b** Heatmap of 63 significantly changed endogenous metabolites in culture medium after *P. distasonis* treatment. Common microbiota pathways such as polyamine, short chain fatty acid, tryptophan were labeled. Red color showed the higher metabolite level and green color showed the lower metabolite level. Heatmap plots were generated by log2 transformation of data (n=5). **c** Metabolic pathways influenced by *P. distasonis* in culture medium.

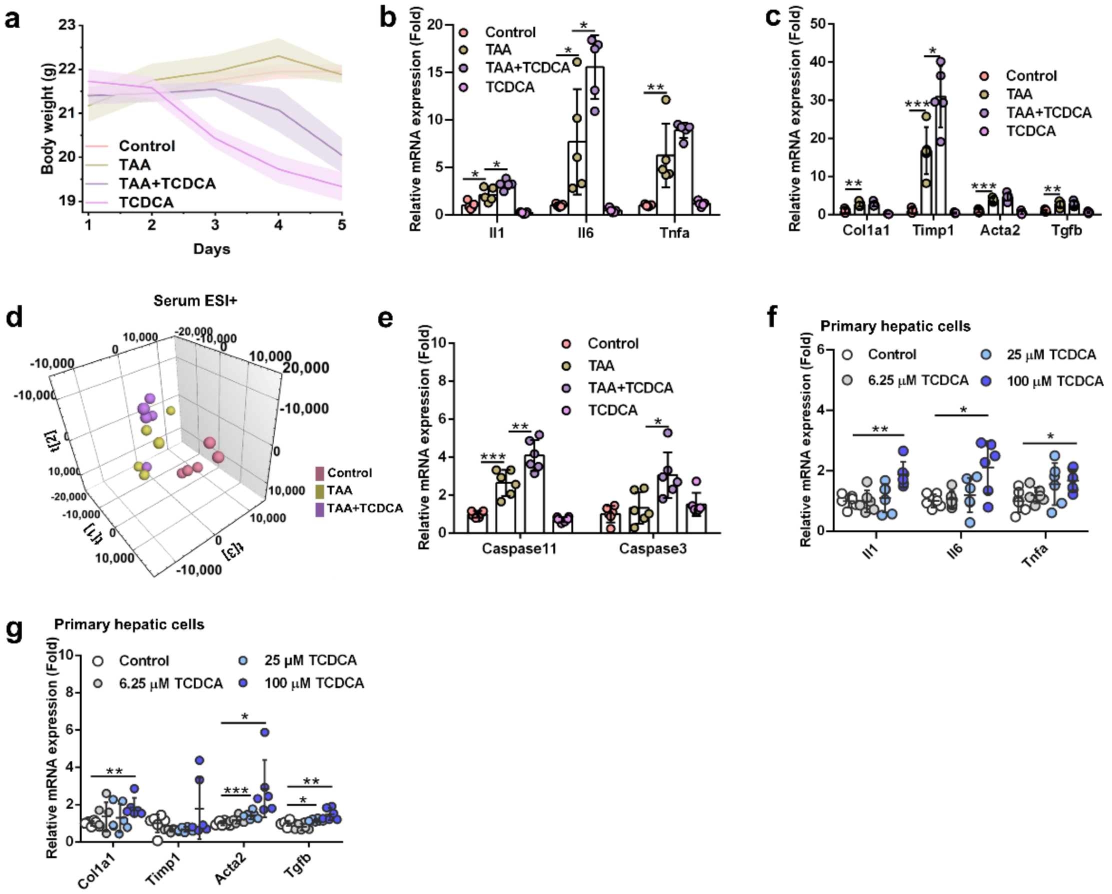

Supplementary Fig. 7 TCDCA potentiates TAA-induced liver injury *in vivo*. Mice were treated with 200 mg/kg TCDCA for 5 days. After TCDCA treatment for 4 days, the mice were treated with 300 mg/kg TCDCA for 1 day (n=6 biologically independent animals). **a** Body weights after TAA and TCDCA co-treatments. **b-c** Proinflammatory factors (**b**) and hepatic fibrosis (**c**) mRNA expression in mice after TAA and TCDCA co-treatments. **d** Principal component analysis (PCA) score plot for serum metabolome detected in ESI+ after TAA and TCDCA co-treatments. Each point represented a sample. **e** Caspase-11 pyroptosis gene expression in liver after TAA and TCDCA co-treatments. **f-g** Proinflammatory factor (**f**) and hepatic fibrosis (**g**) gene expression in mice primary hepatic cell. Primary hepatic cells were treated with 6.25-100 μM TCDCA for 24 h (n=6). Data are presented as the mean ± SD. **P*<0.05, ***P*<0.01, ****P*<0.001.

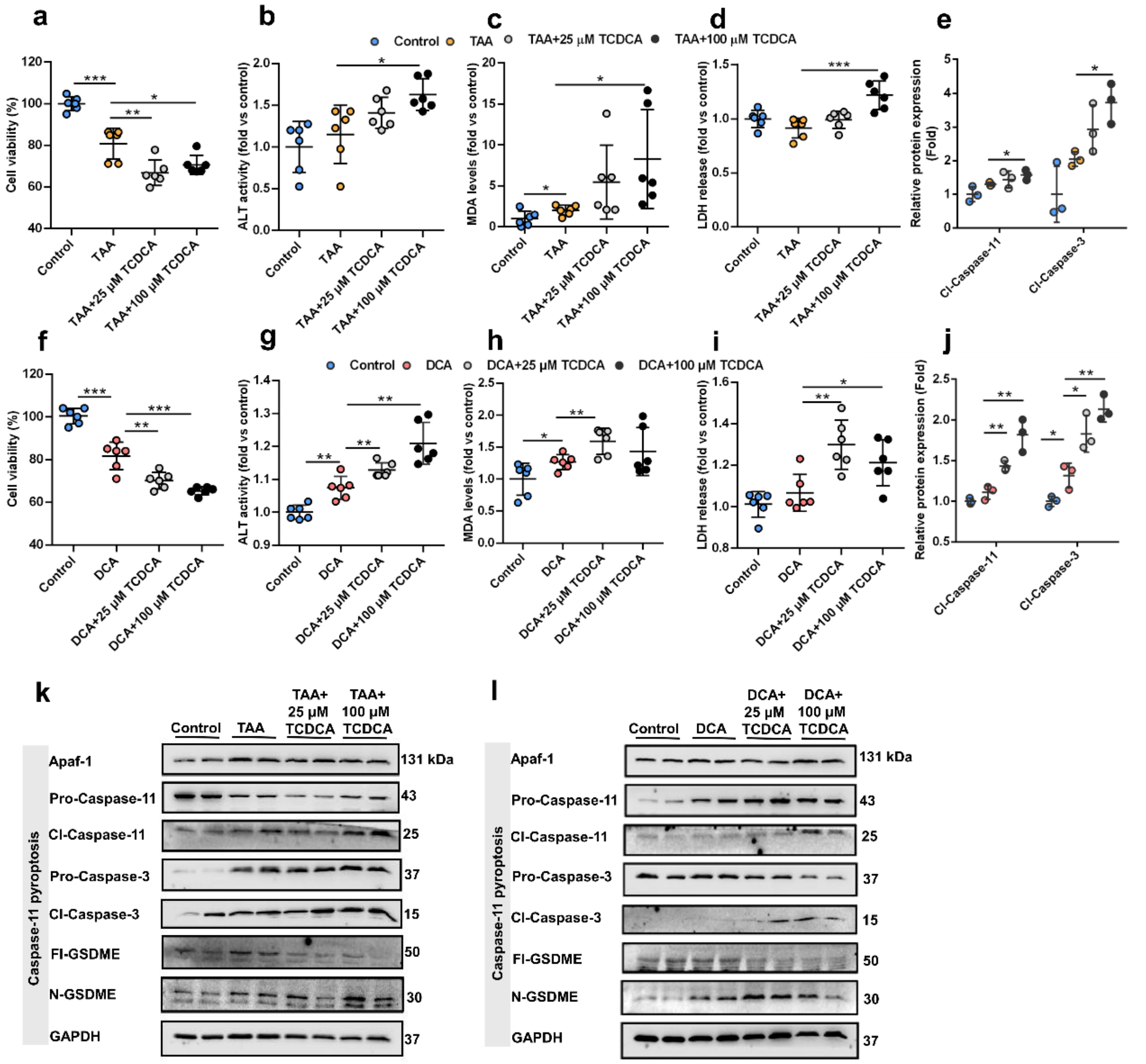

Supplementary Fig. 8 TCDCA potentiates TAA- and DCA-induced cell injury *in vitro*. a-e Cell viability (**a**, n=6 biologically independent cells), ALT (**b**, n=6 biologically independent cells), MDA (**c**, n=6 biologically independent cells), LDH (**d**, n=6 biologically independent cells) and quantitative analysis the Caspase-11 pyroptosis protein level (**e**, n=3 biologically independent cells) after 16 mM thioacetamide (TAA) and 25-100 μM TCDCA co-treatments for 24 h in primary hepatic cells. Western blot images were showed in **k**. **f-j** Cell viability (**f**, n=6 biologically independent cells), ALT (**g**, n=6 biologically independent cells), MDA (**h**, n=6 biologically independent cells), LDH (**i**, n=6 biologically independent cells) and quantitative analysis the Caspase-11 pyroptosis protein level (**j**, n=3 biologically independent cells) after 200 μM deoxycholic acid (DCA) and 25-100 μM TCDCA co-treatments for 24 h in primary hepatic cell. Western blot images were showed in **l**. **k-l** The protein levels of the Caspase-11 pyroptosis pathway (Apaf-1-Caspase-11-Caspase-3-GSDME) after 16 mM TAA (**k**) or 200 μM DCA (**l**) and 25-100 μM TCDCA co-treatments for 24 h in primary hepatic cell. Cl-Caspase-3/11 and N-GSDME were active form of the protein. Data are presented as the mean ± SD. **P*<0.05, ***P*<0.01, ****P*<0.001.

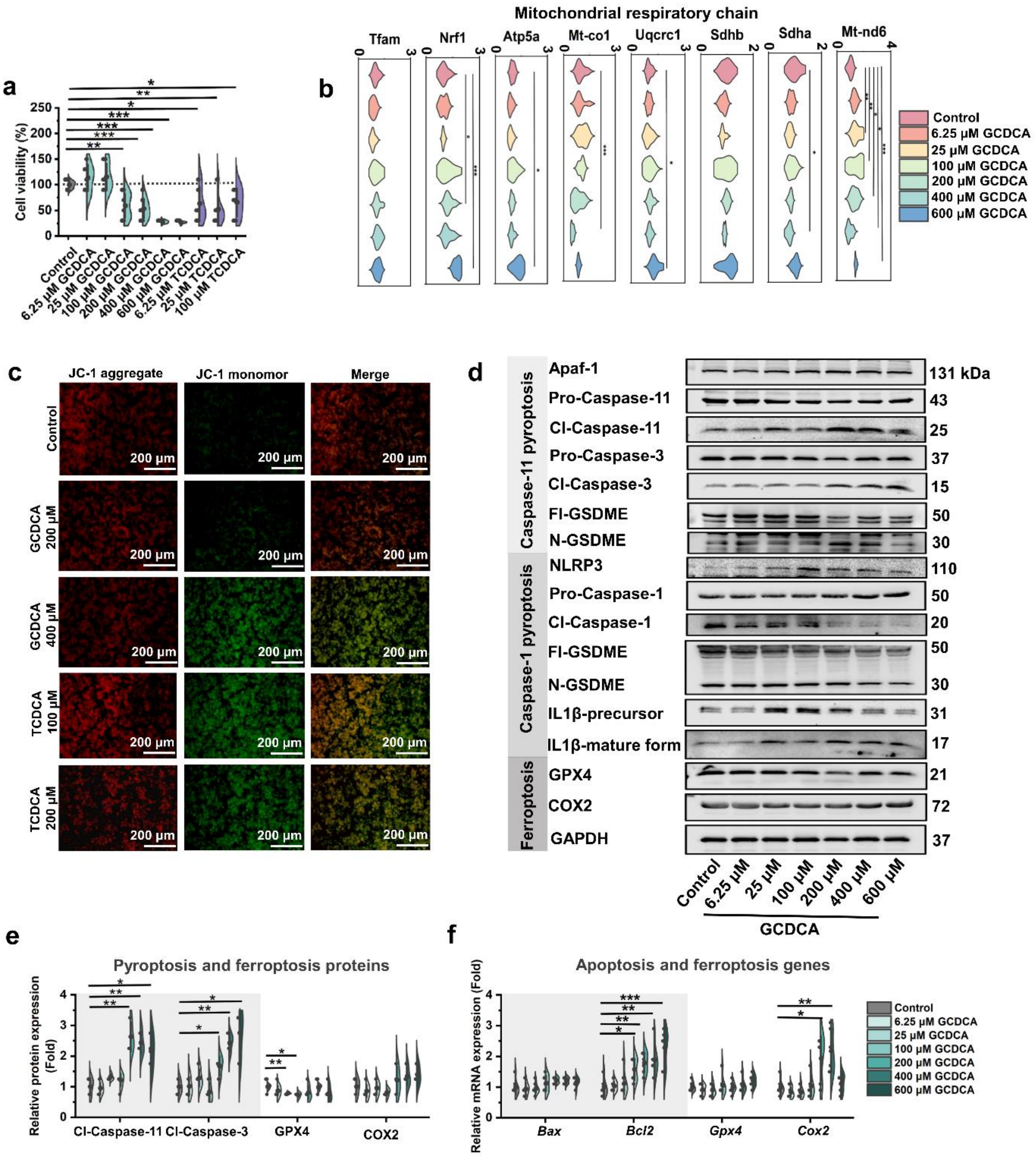

Supplementary Fig. 9 GCDCA induces MPT-Caspase-11 pyroptosis in primary hepatic cells. a Cell viability of primary hepatic cells after treatment with 6.25-600 μM GCDCA and 6.25-100 μM TCDCA for 24 h (n=6). The toxicity of TCDCA was higher than the toxicity of GCDCA in primary hepatic cell. **b** Mitochondrial respiratory chain gene expression was influenced by 6.25-600 μM GCDCA for 24 h (n=6). *Mt-co1* gene expression was decreased and *Nrf1* gene expression was increased, and these results were consistent with TCDCA in Fig. 4f. **c** Dissipation of $\Delta\Psi m$ were detected by the JC-1 assay. Red fluorescence represent JC-1 aggregates in healthy mitochondria, while green fluorescence represent mitochondrial membrane potential collapse. Primary hepatic cells were treated with 200-400 μM GCDCA and 100-200 μM TCDCA for 24 h. **d** Protein levels of Caspase-11 pyroptosis pathway (Apaf-1-Caspase-11-Caspase-3-GSDME), Caspase-1 pyroptosis pathway (NLRP3-Caspase-1-

GSDME/IL1β) and ferroptosis pathway (GPX4 and COX2) after treatment with 6.25-600 μM GCDCA for 24 h in primary hepatic cells. Cl-Caspase-1/3/11, N-GSDME/GSDMD, and IL1β-mature form are the active forms of the proteins. **e** Protein quantification of Caspase-11 pyroptosis pathway (Cl-Caspase-11 and Cl-Caspase-3) and ferroptosis pathway (GPX4 and COX2) after treatment with 6.25-600 μM GCDCA for 24 h in primary hepatic cell (n=3). **f** Gene (mRNA) expression analysis found that apoptosis pathway (*Bax* and *Bcl2*) and ferroptosis pathway (*Gpx4* and *Cox2*) plays an unimportant role in the toxicity of GCDCA compared with Caspase-11 pyroptosis in **e**. Primary hepatic cells were treated with 6.25-600 μM GCDCA for 24 h (n=6). Data are presented as the mean ± SD. **P*<0.05, ***P*<0.01, ****P*<0.001. For violin plot (**a-b**, **e-f**), violin represents kernel density estimation.

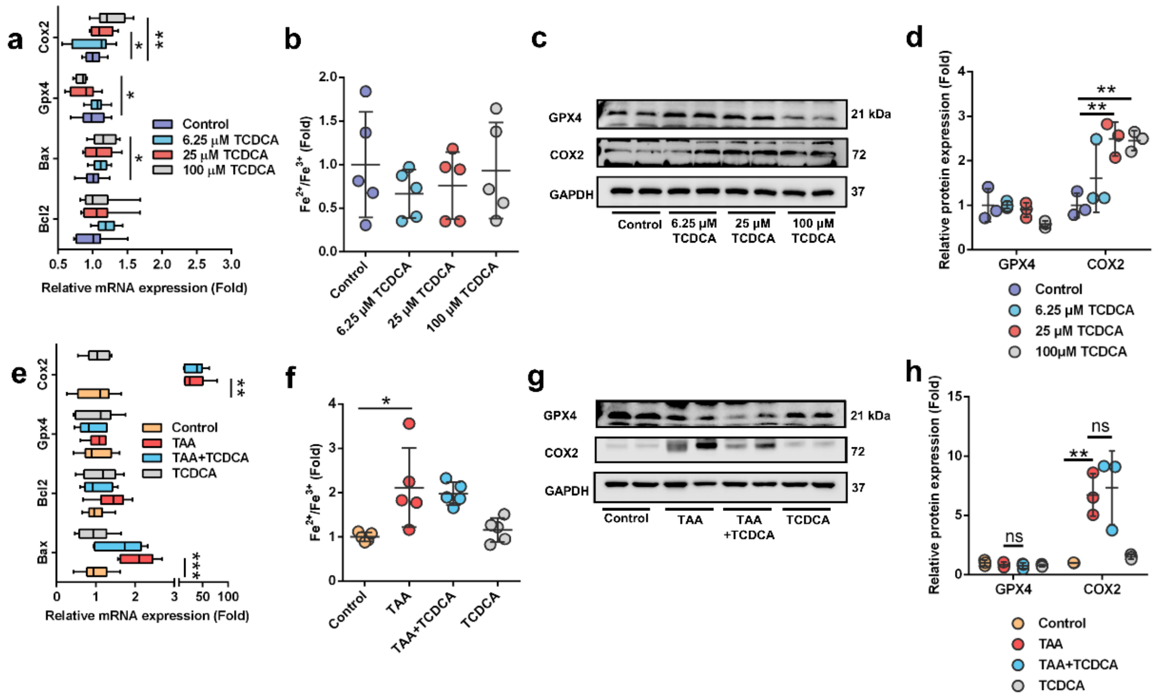

Supplementary Fig. 10 Apoptosis and ferroptosis play an unimportant role in the toxicity of TCDCA *in vitro* and *in vivo*. a Apoptosis gene (mRNA) expression (*Bcl2* and *Bax*) and ferroptosis (mRNA) gene expression (*Gpx4* and *Cox2*) after treat with 6.25-100 μM TCDCA for 24 h in primary hepatic cell (n=12 biologically independent cells). After treatment with TCDCA, the increased *Bax* mRNA expression implied the presence of apoptosis, and the decreased *Gpx4* mRNA expression and the increased *Cox2* mRNA expression implied the role of ferroptosis *in vitro*, but the increased fold was weaker than Caspase-11 pyroptosis (about 6 fold) in Fig. 5d. **b** Fe^{2+}/Fe^{3+} levels are important indexes in ferroptosis. Primary hepatic cells were treated with 6.25-100 μM TCDCA for 24 h (n=5 biologically independent cells). **c-d** GPX4 and COX2 protein levels in primary hepatic cells after treatment with 6.25-100 μM TCDCA for 24 h (n=3 for dot plot). **e** Apoptosis (mRNA) gene expression (*Bcl2* and *Bax*) and ferroptosis (mRNA) gene expression (*Gpx4* and *Cox2*) in mice. Mice were treated with 200 mg/kg TCDCA for 5 days and 300 mg/kg TAA for 1 day (n=6 biologically independent animals). **f** Fe^{2+}/Fe^{3+} levels in mice. Mice were treated as in **e**. n=5 per group. **g-h** GPX4 and COX2 protein levels in mice. Mice were treated as in **e** (n=3 for dot plot). Data are presented as the mean ± SD. *P<0.05, **P<0.01, ***P<0.001. In box plot (**a** and **e**), the center line indicates the median, the edges of the box represent the first and third quartiles, and the whiskers extend to span a 1.5 interquartile range from the edges.

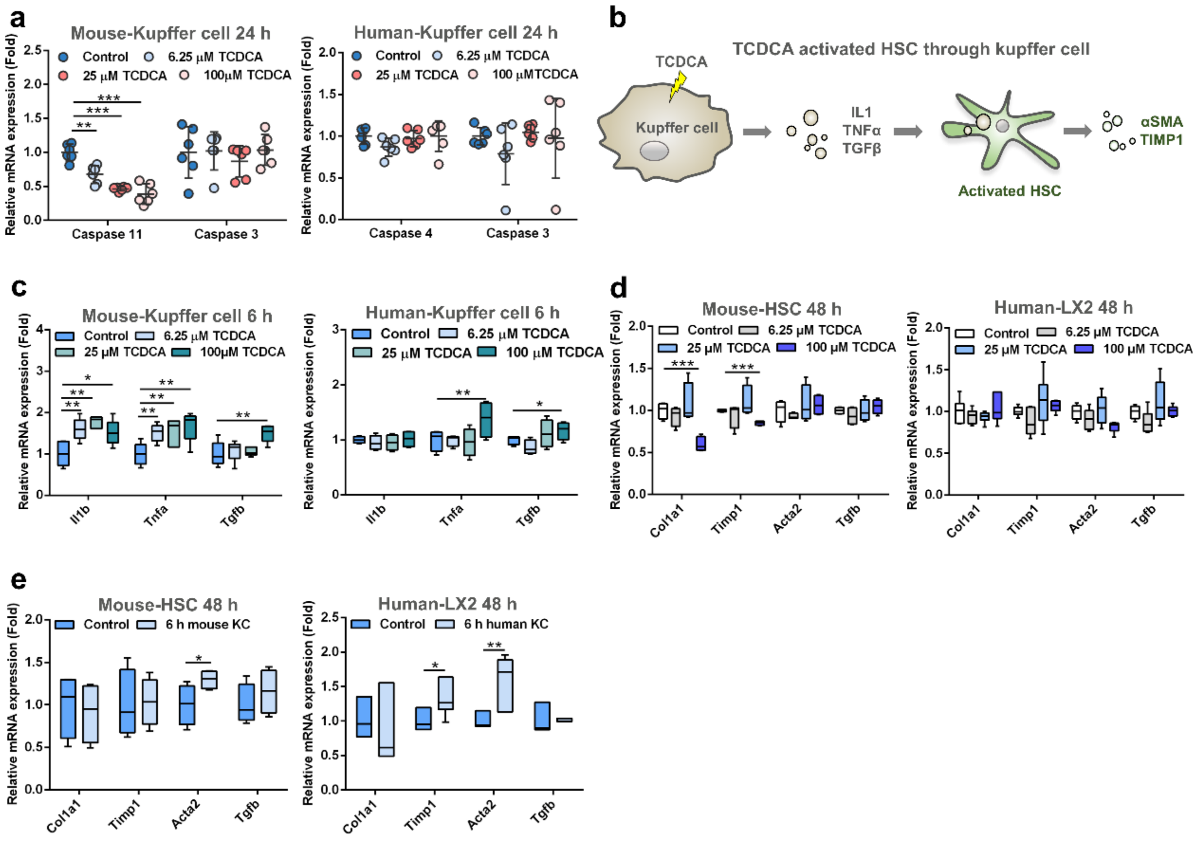

Supplementary Fig. 11 Kupffer cell activate HSC through secreting IL1β, TNFα and TGFβ proteins. a Caspase-11 pyroptosis gene expression after 6.25-100 µM TCDCA treatment in mice and human Kupffer cells for 24 h (n=6). Data are presented as the mean ± SD. **b** Experimental scheme: TCDCA induce the release of IL1, TNFα, and TGFβ proteins in Kupffer cells. The released proteins form Kupffer cell activated HSC, and finally increased the mRNA expression of *Timp1* and *Acta2*. **c** TCDCA activated mouse Kupffer cells (n=6) and human Kupffer cells (n=8) and increased *Tnfa* and *Tgfb* mRNA levels. Kupffer cells were treated with 6.25-100 µM TCDCA for 6 h. **d** Hepatic fibrosis gene expression was not directly simulated by TCDCA in mouse HSCs (n=4) and human HSCs (LX2, n=6). HSCs were treated with 6.25-100 µM TCDCA for 48 h. **e** Mouse and human Kupffer cell supernatants activated mouse HSCs (n=4) and human HSCs (LX2, n=6) respectively, and mouse and human HSCs increased *Acta2* mRNA levels. Kupffer cells were treated with 100 µM TCDCA for 24 h and the supernatant collected. HSCs were treated with the Kupffer cell supernatant (supernatant: medium=1:1). **P*<0.05, ***P*<0.01, ****P*<0.001. In box plot (**c-e**), the center line indicates the median, the edges of the box represent the first and third quartiles, and the whiskers extend to span a 1.5 interquartile range from the edges.

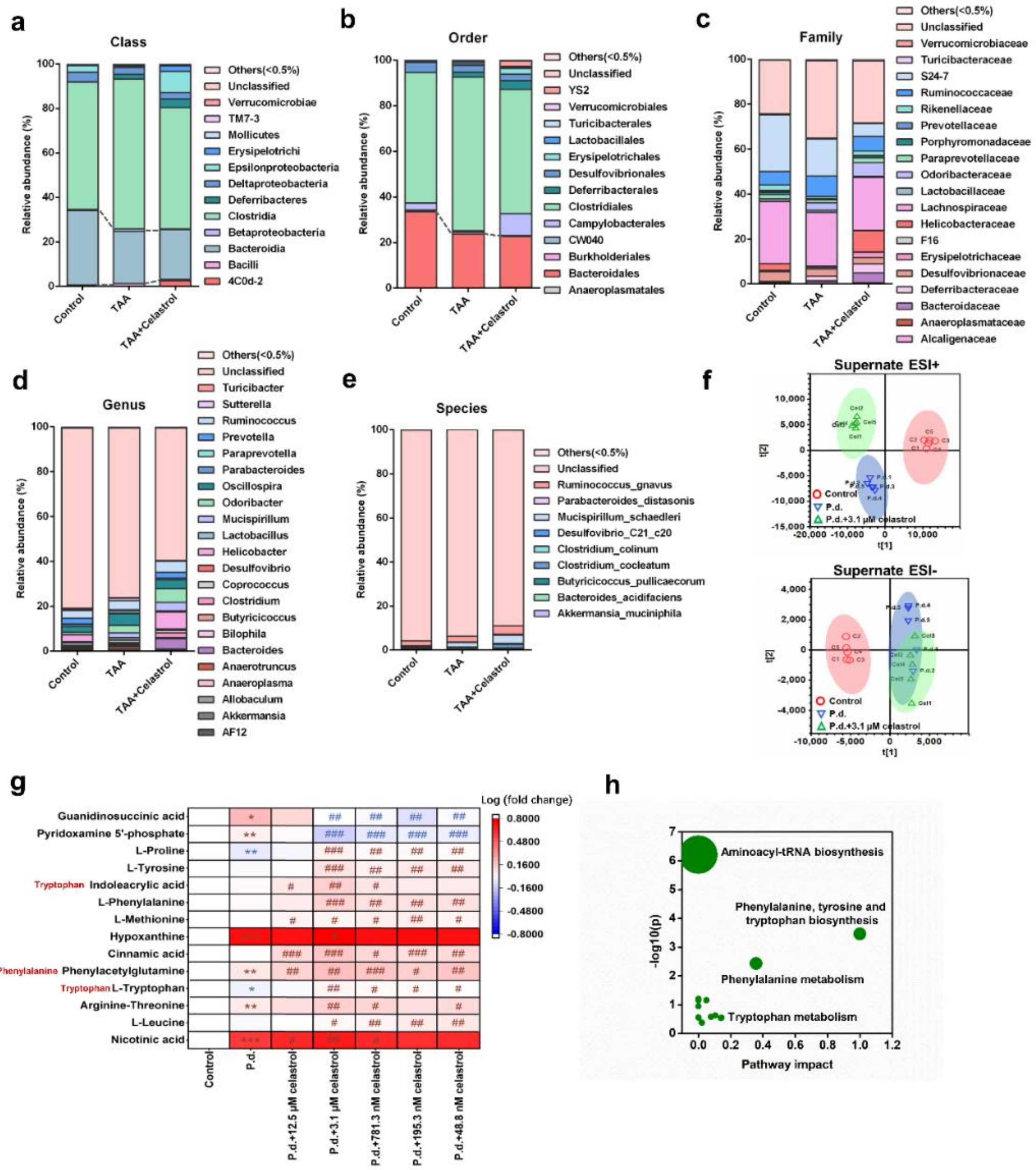

Supplementary Fig. 12 Celastrol increases *P. distasonis* levels *in vivo* and *in vitro*. a-e Relative abundance of class (**a**), order (**b**), family (**c**), genus (**d**) and species (**e**) in mouse cecum content after celastrol and TAA co-treatments (n=5). Mice were treated with 200 mg/kg TAA for 6 weeks. After TAA treatment for 1 week, the mice were treated with celastrol for 5 weeks. **f** Principal component analysis (PCA) score plot for the culture medium metabolome detected in ESI+ and ESI-. Each point represents a sample. *P. distasonis* was treated with 3.1 μM celastrol for 24 h in culture medium (n=5). The original concentration of *P. distasonis* was 22×10^5 CFU/mL. **g** Heatmap of 14 significantly changed endogenous metabolites in culture medium after 48.8 nM-12.5 μM celastrol treatment for 24 h (n=4). Red color shows higher metabolite levels and blue color shows lower metabolite levels. Heatmap plots were generated by log2 transformation of data. Cells were treated as in **f**. **P*<0.05, ***P*<0.01, ****P*<0.001 verses the Control group; #*P*<0.05, ##*P*<0.01, ###*P*<0.001 verse P.d. group. **h** Signaling pathway influenced by celastrol in culture medium. Cell was treated as in **f**.

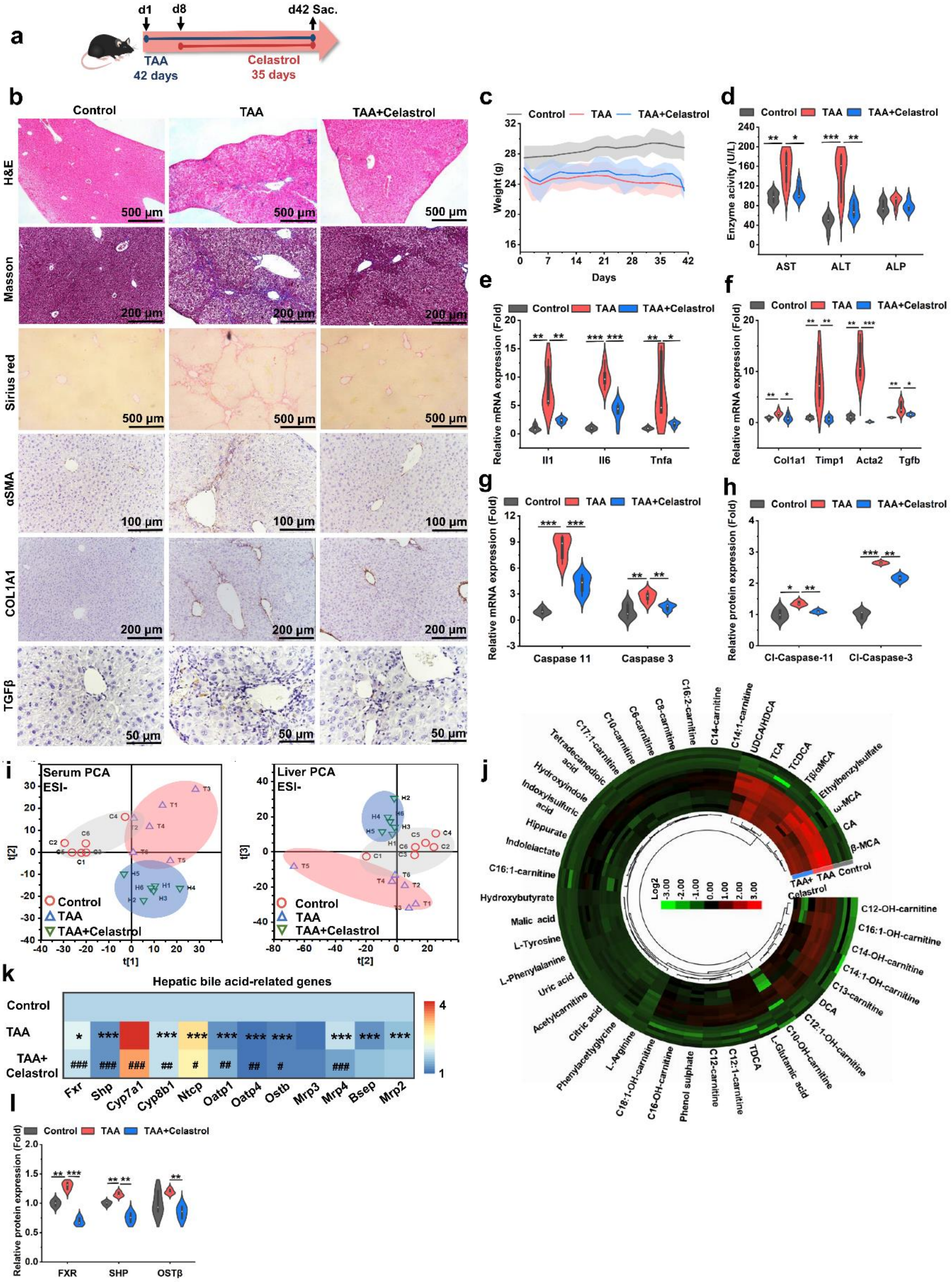

Supplementary Fig. 13 Celastrol protects against TAA-induced hepatic fibrosis in mice. **a** Experimental scheme: mice were treated with 200 mg/kg TAA for 6 weeks. After TAA treatment for 1 week, mice were orally treated with 10 mg/kg celastrol for 5 weeks (three times per week, n=6). **b** Hepatic H&E, Masson trichrome, Sirius red, and immunohistochemical staining for hepatic fibrosis proteins (αSMA, COL1A1 and TGFβ). **c** Body weights after TAA

and celastrol treatments. Data are presented as the mean ± SD. **d** Serum AST, ALT and ALP enzyme activities after TAA and celastrol treatments. **e** Proinflammatory factor gene expression in liver. **f** Hepatic fibrosis gene expression in liver. **g** Hepatic Caspase-11 pyroptosis gene expression. **h** Quantitative analysis of the hepatic Caspase-11 pyroptosis protein expression (n=3). The Cl-Caspase-3/11 was active form of the protein. The western blot images are shown in Fig. 6l. *P<0.05, **P<0.01, ***P<0.001. **i** Principal component analysis (PCA) score plot for the serum and liver metabolome detected in ESI- mode after TAA and celastrol treatments. Each point shows a sample. **j** Heatmap of 45 significantly changed endogenous metabolites including bile acids and gut microbiota metabolites in serum using non-target metabolomics. Red color shows higher metabolite levels and green color shows lower metabolite levels. Heatmap plots were generated by log2 transformation of data. **k** Bile acid gene expression in liver. Red color shows higher gene (mRNA) expression levels and blue color shows lower gene (mRNA) levels. *P<0.05, **P<0.01, ***P<0.001 verse Control group; #P<0.05, ##P<0.01, ###P<0.001 verse TAA group. **l** Quantitative analysis of ileal FXR protein expression (n=3). Western blot images are shown in Fig. 6j. *P<0.05, **P<0.01, ***P<0.001. For violin plot (**d**-**h**, **l**), boxplots represent median with the interquartile range, whiskers indicate adjacent values, violin represents kernel density estimation.

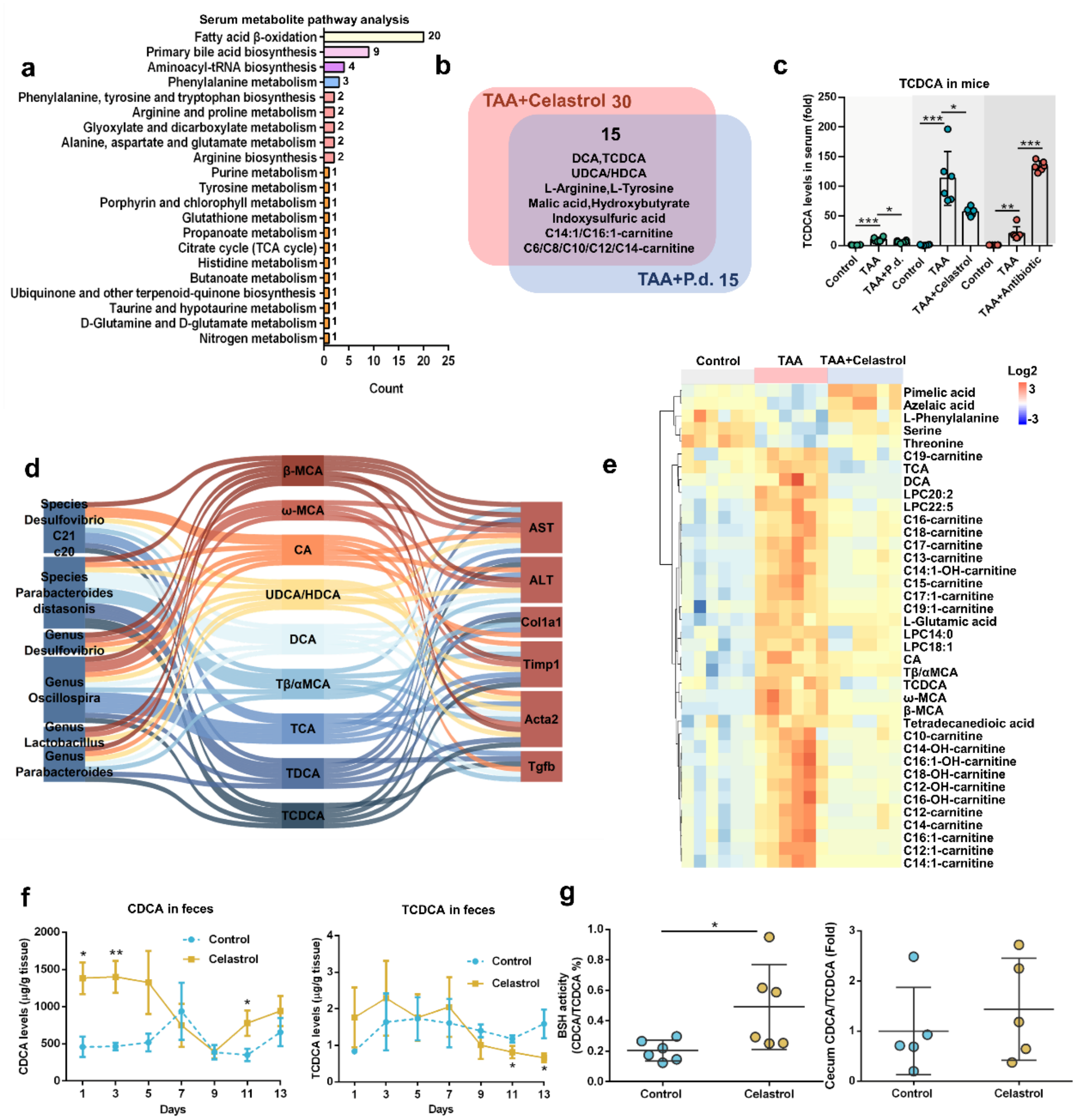

Supplementary Fig. 14 Celastrol improves TAA-induced hepatic fibrosis in mice. a Serum pathways were improved by 5 week 10 mg/kg celastrol treatment in 200 mg/kg TAA-induced hepatic fibrosis in mice. Bile acids play an important role in the protective effect of celastrol. **b** The 15 improved metabolites after celastrol and *P. distasonis* treatments in the serum. Mice were treated as in **a** and Fig. 2a. **c** TCDCA was improved after *P. distasonis* and celastrol treatments and potentiated after antibiotics treatment in mice (n=6). **d** Sankey plot showed the correlation between gut microbes, serum metabolome and host phenotypes after celastrol and TAA co-treatments. **e** Heatmap of 38 significantly changed endogenous metabolites in liver using non-target metabolomics, and various bile acids (e.g., TCA, DCA, CA, Tβ/αMCA,TCDCA, ω-MCA, and β-MCA) were improved (n=6). Red color shows higher metabolite level and blue shows lower metabolite level. Heatmap plots were generated by log2 transformation of data. Mice were treated as in **a**. **f** Celastrol increased CDCA level and decreased TCDCA level in the feces after 2 week 10 mg/kg celastrol treatment indicating that celastrol increased BSH activity (n=5 biologically independent animals). **g** Celastrol increased BSH activity and increased cecum content unconjugated/conjugated bile acid in healthy mice (n=6). Mice were treated with 10 mg/kg celastrol for 2 weeks. *P<0.05, **P<0.01, ***P<0.001.

Data are presented as the mean ± SD.

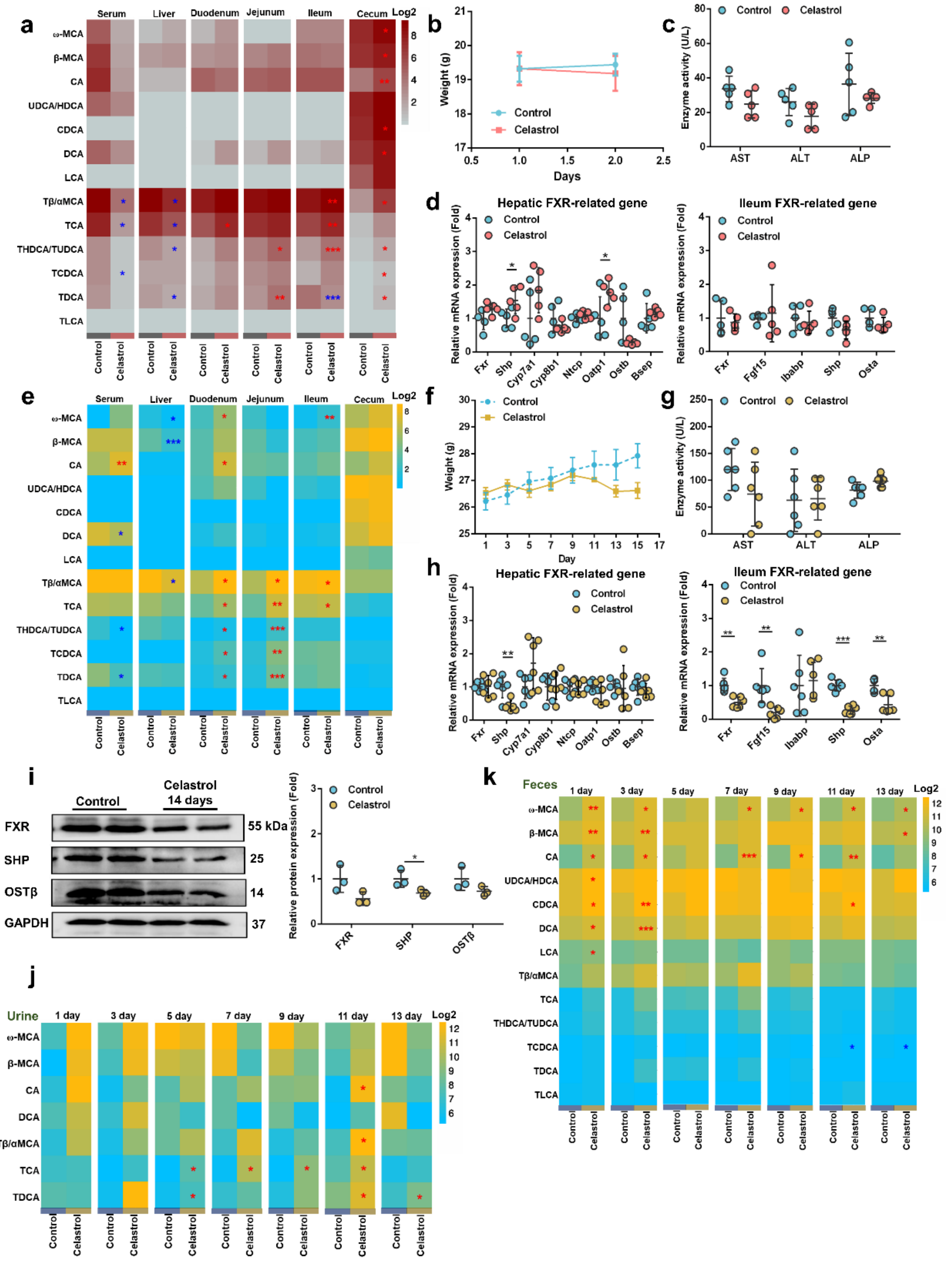

Supplementary Fig. 15 Celastrol promotes the excretion of bile acids in healthy mice. a Bile acid levels in the enterohepatic circulation (serum, liver, duodenum, jejunum, ileum, and cecum content) after one day 10 mg/kg celastrol treatment (n=5). Red color shows higher bile acid levels and gray color showes lower bile acid levels. Heatmap plots were generated by log2 transformation of data. **b-c** Body weight (**b**) and enzyme activities (**c**) after one day 10 mg/kg celastrol treatment (n=5 biologically independent animals). **d** Liver and ileal bile acid gene

expression after one day 10 mg/kg celastrol treatment (n=5 biologically independent animals). **e** Bile acid level in the enterohepatic circulation (serum, liver, duodenum, jejunum, ileum, and cecum content) after a 2 week 10 mg/kg celastrol treatment (n=6 biologically independent animals). Yellow color shows higher bile acid levels and blue color shows lower bile acid levels. Heatmap plots were generated by log2 transformation of data. **f-g** Body weight (**f**) and enzyme activities (**g**) after 2 week 10 mg/kg celastrol treatment (n=5 biologically independent animals). **h** Liver and ileal bile acid gene expression after 2 week 10 mg/kg celastrol treatment (n=6 biologically independent animals). **i** Ileal FXR protein levels after 2 week 10 mg/kg celastrol treatment (n=3 for dot plot). **j-k** Celastrol promoted the excretion of bile acids in urine (**j**) and feces (**k**). Yellow shows higher bile acid levels and blue shows lower bile acid levels. . Heatmap plots were generated by log2 transformation of data. (n=5). *P<0.05, **P<0.01, ***P<0.001. Data are presented as the mean ± SD.

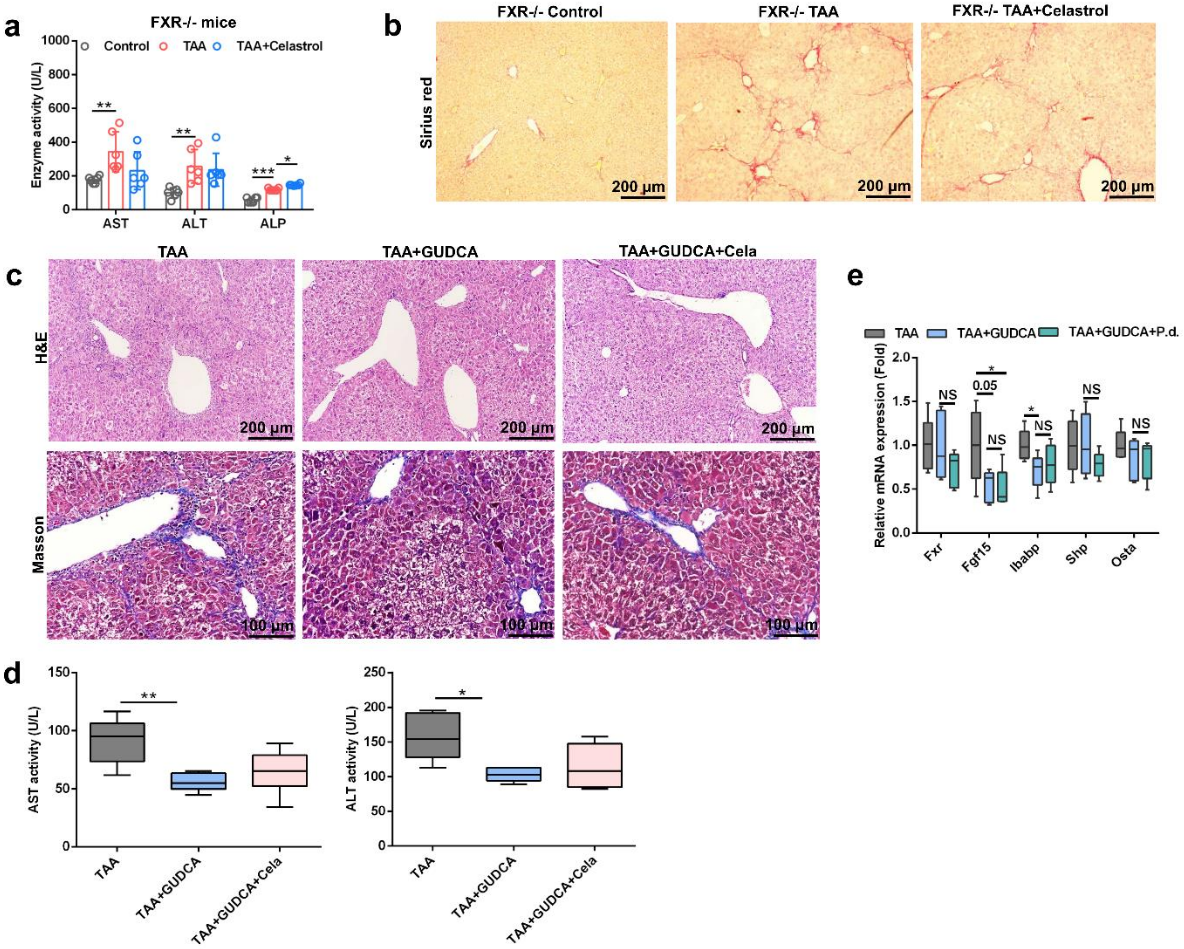

Supplementary Fig. 16 Inhibition of ileal FXR pathway improves TAA-induced hepatic fibrosis in mice. a-b Serum AST, ALT and ALP enzyme activities (**a**) and Sirius red staining (**b**) in *Fxr*-null mice. Mice were treated with 200 mg/kg TAA for 6 weeks and 10 mg/kg celastrol for 5 weeks (n=6 biologically independent animals). Data are presented as the mean ± SD. **c-d** H&E and Masson trichrome staining (**c**) and serum AST and ALT enzyme activities (**d**) after GUDCA treatment. Mice were treated with 200 mg/kg TAA for 6 weeks, 50 mg/kg GUDCA for 5 weeks, and 10 mg/kg celastrol for 5 weeks (n=5 biologically independent animals). *P<0.05, **P<0.01, ***P<0.001. In box plot (**d-e**), the center line indicates the median, the edges of the box represent the first and third quartiles, and the whiskers extend to span a 1.5 interquartile range from the edges.

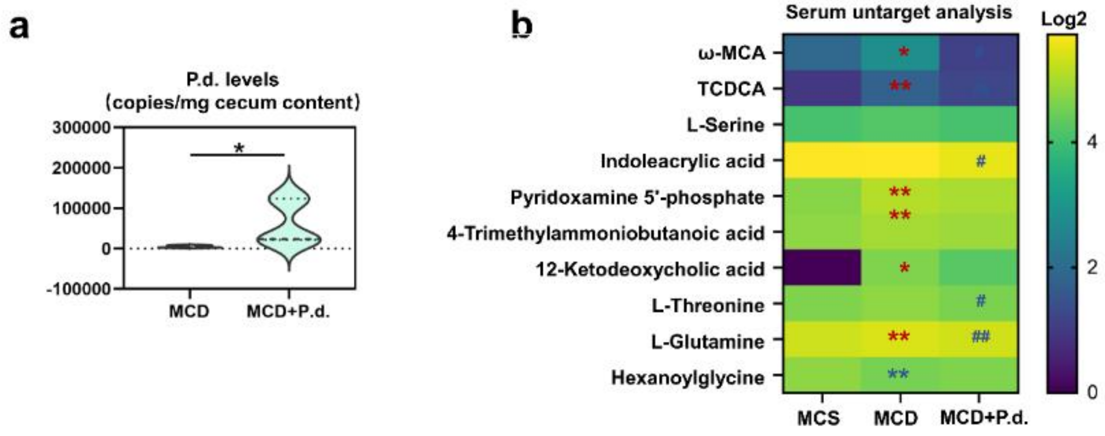

Supplementary Fig. 17 *P. distasonis* protects against MCD diet-induced hepatic fibrosis in mice. Mice were given MCD diet for 6 weeks and 2×10^8 CFU *P. distasonis* for 4 weeks. **a** *P. distasonis* level (n=5). For violin plot, dotted line represent median with the interquartile range, violin represents kernel density estimation. **b** Heatmap of 10 changed endogenous metabolites in serum using non-target metabolomics (n=5). Yellow showed higher metabolite level and blue showed lower metabolite level. Heatmap plots were generated by log2 transformation of data. **P*<0.05, ***P*<0.01 verse MCS group; #*P*<0.05, ##*P*<0.01 verse MCD group.

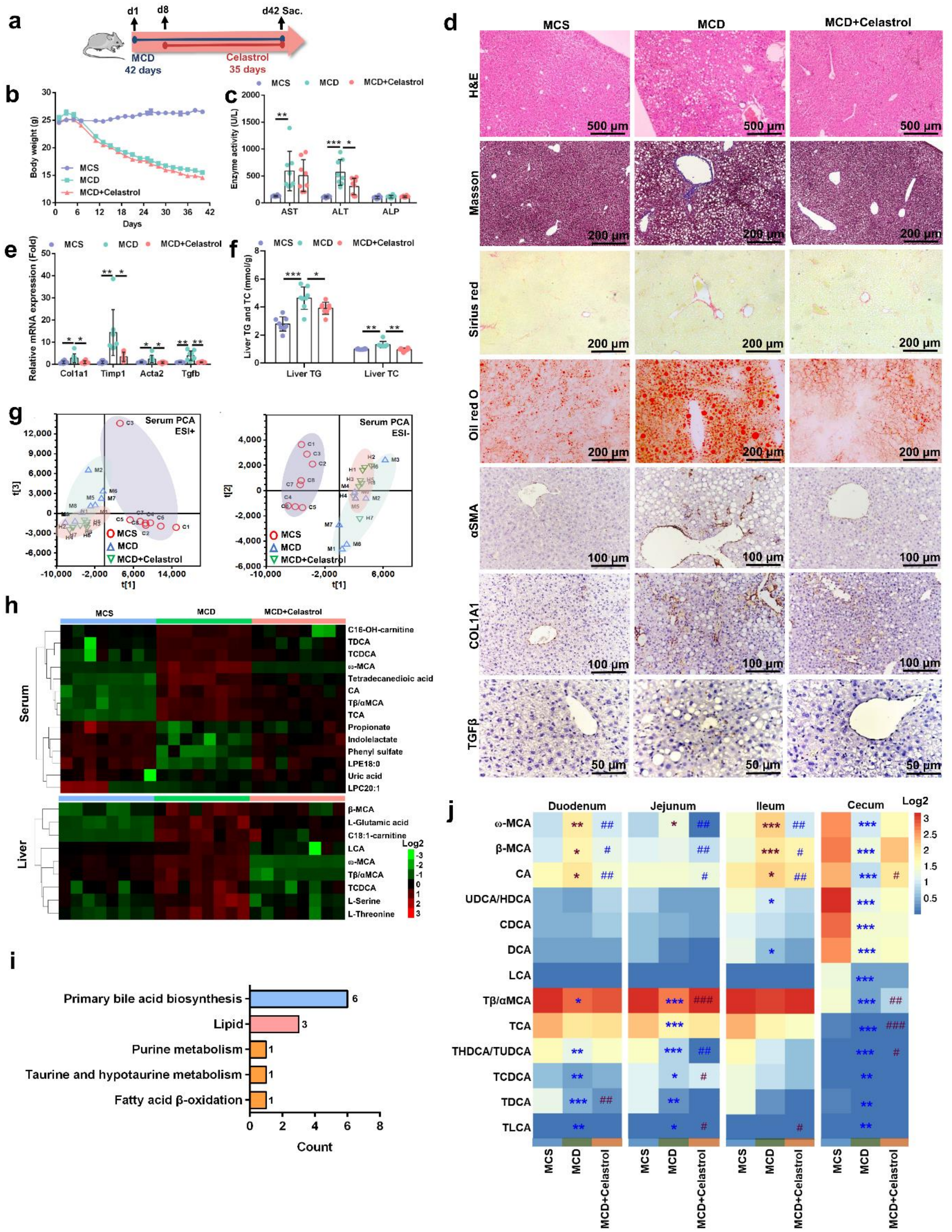

Supplementary Fig. 18 Celastrol improves MCD diet-induced hepatic fibrosis in mice. a Experimental scheme: mice were treated with MCD diet for 6 weeks. After MCD diet treatment for 1 week, mice were treated with 10 mg/kg celastrol for 5 weeks (n=8 biologically independent animals). **b** Body weights. **c** Serum AST, ALT and ALP enzyme activities. **d** H&E, Masson trichrome, Sirius red, Oil red O and immunohistochemistry staining for hepatic fibrosis

proteins (αSMA, COL1A1, and TGFβ). **e** Hepatic fibrosis gene expression. **f** Total triglyceride (TG) and total cholesterol (TC) levels in liver. **P*<0.05, ***P*<0.01, ****P*<0.001. **g** Principal component analysis (PCA) score plot for the serum metabolome detected in ESI+ and ESI- modes. Each point showed a sample. **h** Heatmap of 14 and 9 changed endogenous metabolites in serum and liver using non-target metabolomics. Red color showed higher metabolite level and green showed lower metabolite level. Heatmap plots were generated by log2 transformation of data. **i** Pathway analysis for metabolites in serum after MCD diet and celastrol treatments. **j** Celastrol promoted the excretion of bile acid in duodenum, jejunum, ileum, and cecum content. Red color shows higher bile acid and blue shows lower bile acid. Heatmap plots were generated by log2 transformation of data. **P*<0.05, ***P*<0.01, ****P*<0.001 verse MCS group; #*P*<0.05, ##*P*<0.01, ###*P*<0.001 verse MCD group. Data are presented as the mean ± SD.

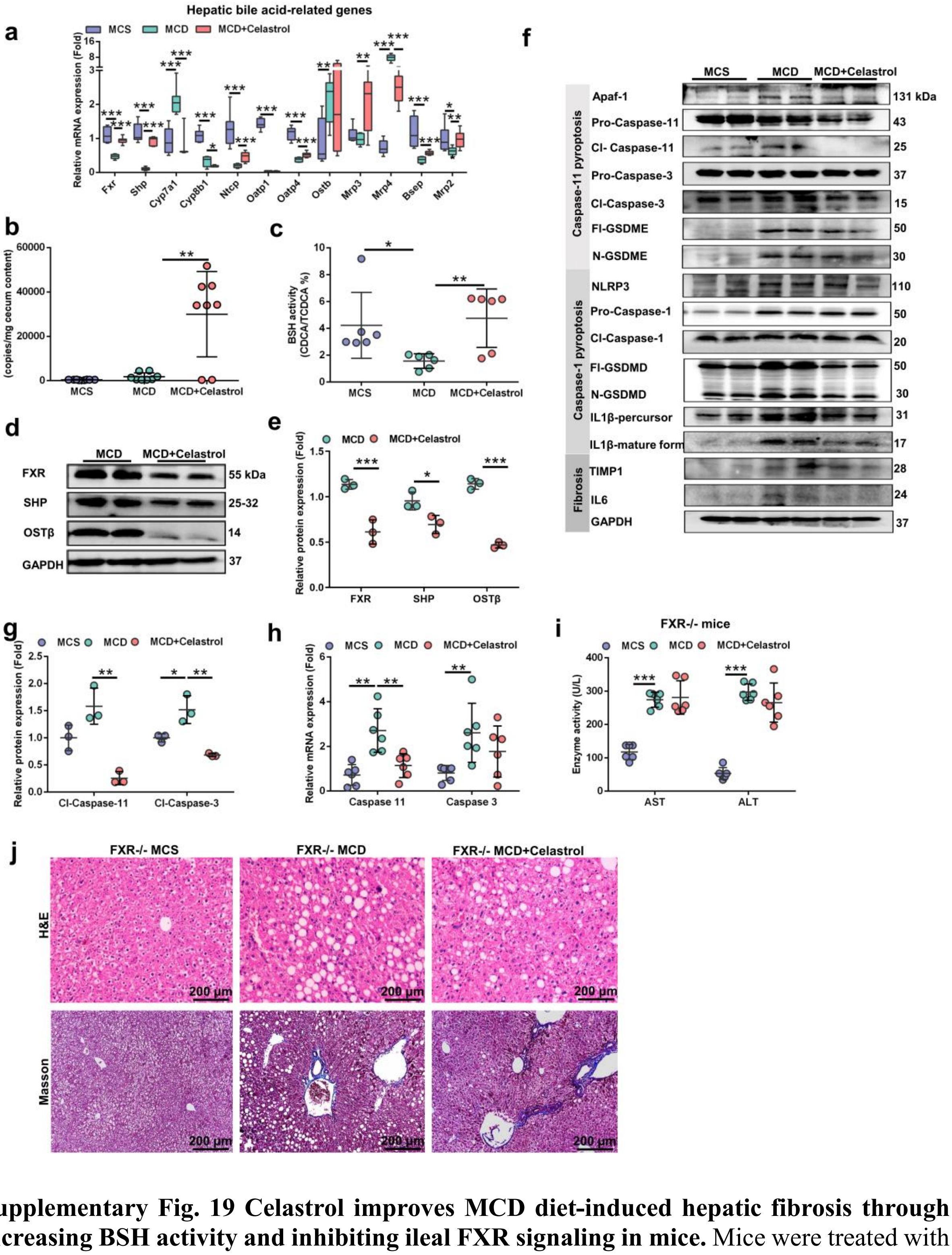

Supplementary Fig. 19 Celastrol improves MCD diet-induced hepatic fibrosis through increasing BSH activity and inhibiting ileal FXR signaling in mice. Mice were treated with MCD diet for 6 weeks. After MCD diet treatment for 1 week, the mice were treated with 10 mg/kg celastrol for 5 weeks (n=8 biologically independent animals). **a** Hepatic bile acid gene expression after MCD diet and celastrol treatments. In box plot, the center line indicates the median, the edges of the box represent the first and third quartiles, and the whiskers extend to span a 1.5 interquartile range from the edges. **b** Celastrol increased *P. distasonis* level in cecum content. **c** Celastrol increased BSH activity in cecum content. **d-e** Ileal FXR protein level. **f**

Caspase-11 pyroptosis pathway (Apaf-1-Caspase-11-Caspase-3-GSDME), Caspase-1 pyroptosis pathway (NLRP3-Caspase-1-GSDME/IL1β), and hepatic fibrosis (TIMP1) protein expression. Cl-Caspase-1/3/11, N-GSDME/GSDMD, and IL1β-mature form were active form of the protein. **g-h** Caspase-11 pyroptosis protein (**g**) and gene (**h**) expression. **i** AST and ALT levels in *Fxr*-null mice after MCD diet and celastrol treatments (n=6). **j** H&E and Masson trichrome staining in *Fxr*-null mice after MCD diet and celastrol treatments. **P*<0.05, ***P*<0.01, ****P*<0.001. Data are presented as the mean ± SD.
