# Supplementary material for: Parabacteroides distasonis ameliorates hepatic fibrosis potentially via modulating intestinal bile acid metabolism and hepatocyte pyroptosis in male mice
Source: Nat Commun. 2023 Apr 1;14:1829. doi: 10.1038/s41467-023-37459-z (PMC10067939; doi:10.1038/s41467-023-37459-z)
Supplement: Supplementary file 2 — Reporting Summary [file 41467_2023_37459_MOESM2_ESM.pdf]

nature portfolio

Corresponding author(s): Fei Li &Yingmei Tang

Last updated by author(s): 2022.12.13

Reporting Summary

Nature Portfolio wishes to improve the reproducibility of the work that we publish. This form provides structure for consistency and transparency in reporting. For further information on Nature Portfolio policies, see our Editorial Policies and the Editorial Policy Checklist.

Statistics

For all statistical analyses, confirm that the following items are present in the figure legend, table legend, main text, or Methods section.

n/a	Confirmed	
☐	☒	The exact sample size (*n*) for each experimental group/condition, given as a discrete number and unit of measurement
☐	☒	A statement on whether measurements were taken from distinct samples or whether the same sample was measured repeatedly
☐	☒	The statistical test(s) used AND whether they are one- or two-sided<br>*Only common tests should be described solely by name; describe more complex techniques in the Methods section.*
☐	☒	A description of all covariates tested
☐	☒	A description of any assumptions or corrections, such as tests of normality and adjustment for multiple comparisons
☐	☒	A full description of the statistical parameters including central tendency (e.g. means) or other basic estimates (e.g. regression coefficient) AND variation (e.g. standard deviation) or associated estimates of uncertainty (e.g. confidence intervals)
☐	☒	For null hypothesis testing, the test statistic (e.g. *F*, *t*, *r*) with confidence intervals, effect sizes, degrees of freedom and *P* value noted<br>*Give P values as exact values whenever suitable.*
☒	☐	For Bayesian analysis, information on the choice of priors and Markov chain Monte Carlo settings
☒	☐	For hierarchical and complex designs, identification of the appropriate level for tests and full reporting of outcomes
☐	☒	Estimates of effect sizes (e.g. Cohen's *d*, Pearson's *r*), indicating how they were calculated

Our web collection on statistics for biologists contains articles on many of the points above.

Software and code

Policy information about availability of computer code

Data collection	Mass SpectRometry data was collected with Thermo Xcalibur Qual Browser (Thermo-Fisher).
Data analysis	Graphpad Prism 6.01, OriginPro 9.0, Simcap 13.0, MetaboAnalyst 4.0

For manuscripts utilizing custom algorithms or software that are central to the research but not yet described in published literature, software must be made available to editors and reviewers. We strongly encourage code deposition in a community repository (e.g. GitHub). See the Nature Portfolio guidelines for submitting code & software for further information.

Data

Policy information about availability of data

All manuscripts must include a data availability statement. This statement should provide the following information, where applicable:

- Accession codes, unique identifiers, or web links for publicly available datasets
- A description of any restrictions on data availability
- For clinical datasets or third party data, please ensure that the statement adheres to our policy

The 16S rRNA gene sequencing data generated in this study were deposited in the NCBI SRA https://www.ncbi.nlm.nih.gov/sra/ (SRA accession: SRR22703511-SRR22703525). All raw mass spectrometry data are available through the MetaboLights with the identifier MTBLS6742, MTBLS6732, and MTBLS6728. Source data are provided with this paper.

Human research participants

Policy information about studies involving human research participants and Sex and Gender in Research.

Reporting on sex and gender	Number of female participants for each of the groups analyzed are given in Supplementary Table 1-3. Information for individual participant level is given in raw data.<br>Differences between male and female participants were not analyzed due to small group sizes.
Population characteristics	Patient characteristics are described in the Supplementary Table 1-3.
Recruitment	All volunteers were evaluated by clinicians during the study ensuring that they remained in good health. Eligible patients were recruited by investigators at the Secondary Affiliated Hospital of Kunming Medical University. All patients were hospitalized patients. Severe hepatic fibrosis was selected by liver puncture and Child score. Other biases such as regional difference may be present because these patients only showed the result in southwest china.
Ethics oversight	The study protocol was approved by the Conjoint Health Research Ethics Board of the Secondary Affiliated Hospital of Kunming Medical University (Registration number: PJ-2017-25). Informed consent has been obtained from study participants.

Note that full information on the approval of the study protocol must also be provided in the manuscript.

Field-specific reporting

Please select the one below that is the best fit for your research. If you are not sure, read the appropriate sections before making your selection.

☒ Life sciences ☐ Behavioural & social sciences ☐ Ecological, evolutionary & environmental sciences

For a reference copy of the document with all sections, see nature.com/documents/nr-reporting-summary-flat.pdf

Life sciences study design

All studies must disclose on these points even when the disclosure is negative.

Sample size	No sample size calculation for clinical samples was performed to pre-determine sample size. It is based on the amount for clinical patient in hospital in a certain period of time. The number of animal and cell samples in this study is comparable to the other studies.
Data exclusions	No data were excluded.
Replication	Reproducibility of experimental findings was verified through multiplicating. Clinical samples were performed in 3 biological replicates to ensure reproducibility of our findings, and data from all replicates were included in this study. Animal experiments were carried out with twice and cell experiments were carried out with twice, all attempts at replication were successful.
Randomization	Animals were assigned into experimental groups randomly. The patients were randomly selected, we do not choose specific etiology. Cell were random allocation and given drugs,
Blinding	For mass spectrometry experiments, the samples were provided to the mass spectrometrists in a blinded fashion with only replicates. In the cell and animal experiment, investigators were not blinded to group allocation because the investigators should give the drug to the mice and cell on specific days.

Reporting for specific materials, systems and methods

We require information from authors about some types of materials, experimental systems and methods used in many studies. Here, indicate whether each material, system or method listed is relevant to your study. If you are not sure if a list item applies to your research, read the appropriate section before selecting a response.

Materials & experimental systems

n/a	Involved in the study	
☐	☒	Antibodies
☐	☒	Eukaryotic cell lines
☒	☐	Palaeontology and archaeology
☐	☒	Animals and other organisms
☒	☐	Clinical data
☒	☐	Dual use research of concern

Methods

n/a	Involved in the study	
☒	☐	ChIP-seq
☒	☐	Flow cytometry
☒	☐	MRI-based neuroimaging

Antibodies

Antibodies used

Apaf-1 (sc-65891, Santa Cruz Biotechnology, USA; WB 1:200 dilution), Caspase-11 (ab180673, Abcam, UK; WB 1:1000 dilution), Caspase-3 (9662s, Cell Signaling Technology, USA; WB 1:1000 dilution), GSDME (ab215191, Abcam, UK; WB 1:1000 dilution), αSMA (D4K9N, 19245s, Cell Signaling Technology, USA; WB 1:1000 dilution; IF 1:100 dilution; IHC 1:800 dilution), COL1A1 (E8F4L, 72026s, Cell Signaling Technology, USA; WB 1:1000 dilution; IHC 1:100 dilution), TIMP1 (ab179580, Abcam, UK; WB 1:1000 dilution), TGFβ (ab215715, Abcam, UK; WB 1:1000 dilution; IHC 1:200 dilution), IL6 (D5W4V, 12912s, Cell Signaling Technology, USA; WB 1:1000 dilution; IF 1:200 dilution), IL1β (D3H1Z, 12507s, Cell Signaling Technology, USA; WB 1:1000 dilution), FXR (sc-25309, Santa Cruz Biotechnology, USA; WB 1:100 dilution), SHP (ab232841, Abcam, UK; WB 1:500 dilution), OSTβ (bs-2128R, Bioss, China; WB 1:1000 dilution), NLRP3 (D4D8T, 15101s, Cell Signaling Technology, USA; WB 1:1000 dilution), Caspase-1 (E2G2I, 89332s, Cell Signaling Technology, USA; WB 1:1000 dilution), GSDMD (sc-393656, Santa Cruz Biotechnology, USA; WB 1:200 dilution), CK19 (ab254186, Abcam, UK; IF 1:100 dilution), CD31 (ab7388, Abcam, UK; IF 1:100 dilution), GPX4 (sc-166570, Santa Cruz Biotechnology, USA; WB 1:200 dilution), COX2 (sc-376861, Santa Cruz Biotechnology, USA; WB 1:200 dilution) and GAPDH (14C10, 2119s, Cell Signaling Technology, USA; WB 1:1000 dilution).

Validation

Commercial available Western blot and immunofluorescence antibodies were selected based on their antigen specificity and suggested application as described on the manufacturer's website and data sheets.
Apaf-1 (sc-65891, Santa Cruz Biotechnology, USA) https://www.scbt.com/p/apaf-1-antibody-5e11?requestFrom=search
FXR (sc-25309, Santa Cruz Biotechnology, USA) https://www.scbt.com/p/fxr-antibody-d-3?requestFrom=search
GSDMD (sc-393656, Santa Cruz Biotechnology, USA) https://www.scbt.com/p/gsdmdc1-antibody-a-7?requestFrom=search
GPX4 (sc-166570, Santa Cruz Biotechnology, USA) https://www.scbt.com/p/gpx-4-antibody-e-12?requestFrom=search
COX2 (sc-376861, Santa Cruz Biotechnology, USA) https://www.scbt.com/p/cox-2-antibody-h-3?requestFrom=search
Caspase-11 (ab180673, Abcam, UK) https://www.abcam.cn/caspase-11-antibody-epr18628-ab180673.html
GSDME (ab215191, Abcam, UK) https://www.abcam.cn/dfna5gsdme-antibody-epr19859-n-terminal-ab215191.html
TIMP1 (ab179580, Abcam, UK) https://www.abcam.cn/timp1-antibody-epr16616-ab179580.html
TGFβ (ab215715, Abcam, UK) https://www.abcam.cn/tgf-beta-1-antibody-epr21143-ab215715.html
SHP (ab232841, Abcam, UK) https://www.abcam.cn/nr0b2-antibody-ab232841.html
CK19 (ab254186, Abcam, UK) https://www.abcam.cn/cytokeratin-19-antibody-lp2k-ab254186.html
CD31 (ab7388, Abcam, UK) https://www.abcam.cn/cd31-antibody-mec-746-ab7388.html
Caspase-3 (9662s, Cell Signaling Technology, USA) https://www.cellsignal.cn/products/primary-antibodies/caspase-3-antibody/9662?site-search-type=Products&N=4294956287&Ntt=9662s&fromPage=plp&_requestid=4068160
αSMA (D4K9N, Cell Signaling Technology, USA) https://www.cellsignal.cn/products/primary-antibodies/a-smooth-muscle-actin-d4k9n-xp-rabbit-mab/19245?site-search-type=Products&N=4294956287&Ntt=d4k9n&fromPage=plp
COL1A1 (E8F4L, Cell Signaling Technology, USA) https://www.cellsignal.cn/products/primary-antibodies/col1a1-e8f4l-xp-rabbit-mab/72026?site-search-type=Products&N=4294956287&Ntt=e8f4l&fromPage=plp
IL6 (D5W4V, Cell Signaling Technology, USA) https://www.cellsignal.cn/products/primary-antibodies/il-6-d5w4v-xp-rabbit-mab-mouse-specific/12912?site-search-type=Products&N=4294956287&Ntt=d5w4v&fromPage=plp
IL1β (D3H1Z, Cell Signaling Technology, USA) https://www.cellsignal.cn/products/primary-antibodies/il-1b-d3h1z-rabbit-mab-mouse-specific/12507?site-search-type=Products&N=4294956287&Ntt=d3h1z&fromPage=plp
NLRP3 (D4D8T, Cell Signaling Technology, USA) https://www.cellsignal.cn/products/primary-antibodies/nlrp3-d4d8t-rabbit-mab/15101?site-search-type=Products&N=4294956287&Ntt=d4d8t&fromPage=plp&_requestid=4068447
Caspase-1 (E2G2I, Cell Signaling Technology, USA) https://www.cellsignal.cn/products/primary-antibodies/cleaved-caspase-1-asp296-e2g2i-rabbit-mab/89332?site-search-type=Products&N=4294956287&Ntt=e2g2i&fromPage=plp
GAPDH (14C10, Cell Signaling Technology, USA) https://www.cellsignal.cn/products/primary-antibodies/gapdh-14c10-rabbit-mab/2118?site-search-type=Products&N=4294956287&Ntt=14c10&fromPage=plp
OSTβ (bs-2128R, Bioss, China) https://www.biossusa.com/products/bs-2128r

Eukaryotic cell lines

Policy information about cell lines and Sex and Gender in Research

Cell line source(s)	Caco2 and HEK293 were obtained from American Type Culture Collection (Manassas, VA, USA).
Authentication	None of the cell lines have been authenticated.
Mycoplasma contamination	The cell line has tested negative for bacteria, fungi, and mycoplasma.
Commonly misidentified lines (See ICLAC register)	None

Palaeontology and Archaeology

Specimen provenance

Provide provenance information for specimens and describe permits that were obtained for the work (including the name of the issuing authority, the date of issue, and any identifying information). Permits should encompass collection and, where applicable, export.

Specimen deposition

Indicate where the specimens have been deposited to permit free access by other researchers.

Dating methods

If new dates are provided, describe how they were obtained (e.g. collection, storage, sample pretreatment and measurement), where they were obtained (i.e. lab name), the calibration program and the protocol for quality assurance OR state that no new dates are

provided.

☐ Tick this box to confirm that the raw and calibrated dates are available in the paper or in Supplementary Information.

Ethics oversight	*Identify the organization(s) that approved or provided guidance on the study protocol, OR state that no ethical approval or guidance was required and explain why not.*

Note that full information on the approval of the study protocol must also be provided in the manuscript.

Animals and other research organisms

Policy information about studies involving animals; ARRIVE guidelines recommended for reporting animal research, and Sex and Gender in Research

Laboratory animals	Male 6-week-old Fxr-null mice (C57BL/6J background) were previously described. Male 6-week-old C57BL/6J mice were purchased from GemPharmatech Co., Ltd. (Jiangsu, China) and maintained under a standard 12-h light/12-h dark cycle environment with free access to water and rodent chow. Mice were housed in standard cages (45×29×12cm) and maintained in a temperature 22±1°C and humidity-controlled room (40–65%) on a 12 h light cycle with ad libitum access to water and a standard laboratory chow diet.
Wild animals	No wild animals were used in the study
Reporting on sex	Male mice were used in the study.
Field-collected samples	The study did not involve sample collected from the field.
Ethics oversight	All animal experiments were approved by the Animal Care and Use Committee of West China Hospital, Sichuan University (20220217001).

Note that full information on the approval of the study protocol must also be provided in the manuscript.

Clinical data

Policy information about clinical studies
All manuscripts should comply with the ICMJE guidelines for publication of clinical research and a completed CONSORT checklist must be included with all submissions.

Clinical trial registration	The study protocol was approved by the Conjoint Health Research Ethics Board of the Secondary Affiliated Hospital of Kunming Medical University (Registration number: PJ-2017-25).
Study protocol	*Note where the full trial protocol can be accessed OR if not available, explain why.*
Data collection	*Describe the settings and locales of data collection, noting the time periods of recruitment and data collection.*
Outcomes	*Describe how you pre-defined primary and secondary outcome measures and how you assessed these measures.*

Dual use research of concern

Policy information about dual use research of concern

Hazards

Could the accidental, deliberate or reckless misuse of agents or technologies generated in the work, or the application of information presented in the manuscript, pose a threat to:

No	Yes	
☐	☐	Public health
☐	☐	National security
☐	☐	Crops and/or livestock
☐	☐	Ecosystems
☐	☐	Any other significant area

Experiments of concern

Does the work involve any of these experiments of concern:

No	Yes	
☐	☐	Demonstrate how to render a vaccine ineffective
☐	☐	Confer resistance to therapeutically useful antibiotics or antiviral agents
☐	☐	Enhance the virulence of a pathogen or render a nonpathogen virulent
☐	☐	Increase transmissibility of a pathogen
☐	☐	Alter the host range of a pathogen
☐	☐	Enable evasion of diagnostic/detection modalities
☐	☐	Enable the weaponization of a biological agent or toxin
☐	☐	Any other potentially harmful combination of experiments and agents

ChIP-seq

Data deposition

☐ Confirm that both raw and final processed data have been deposited in a public database such as GEO.

☐ Confirm that you have deposited or provided access to graph files (e.g. BED files) for the called peaks.

Data access links<br>*May remain private before publication.*	*For "Initial submission" or "Revised version" documents, provide reviewer access links. For your "Final submission" document, provide a link to the deposited data.*
Files in database submission	*Provide a list of all files available in the database submission.*
Genome browser session<br>(e.g. UCSC)	*Provide a link to an anonymized genome browser session for "Initial submission" and "Revised version" documents only, to enable peer review. Write "no longer applicable" for "Final submission" documents.*

Methodology

Replicates	*Describe the experimental replicates, specifying number, type and replicate agreement.*
Sequencing depth	*Describe the sequencing depth for each experiment, providing the total number of reads, uniquely mapped reads, length of reads and whether they were paired- or single-end.*
Antibodies	*Describe the antibodies used for the ChIP-seq experiments; as applicable, provide supplier name, catalog number, clone name, and lot number.*
Peak calling parameters	*Specify the command line program and parameters used for read mapping and peak calling, including the ChIP, control and index files used.*
Data quality	*Describe the methods used to ensure data quality in full detail, including how many peaks are at FDR 5% and above 5-fold enrichment.*
Software	*Describe the software used to collect and analyze the ChIP-seq data. For custom code that has been deposited into a community repository, provide accession details.*

Flow Cytometry

Plots

Confirm that:

☐ The axis labels state the marker and fluorochrome used (e.g. CD4-FITC).

☐ The axis scales are clearly visible. Include numbers along axes only for bottom left plot of group (a 'group' is an analysis of identical markers).

☐ All plots are contour plots with outliers or pseudocolor plots.

☐ A numerical value for number of cells or percentage (with statistics) is provided.

Methodology

Sample preparation	*Describe the sample preparation, detailing the biological source of the cells and any tissue processing steps used.*
Instrument	*Identify the instrument used for data collection, specifying make and model number.*

Software	*Describe the software used to collect and analyze the flow cytometry data. For custom code that has been deposited into a community repository, provide accession details.*
Cell population abundance	*Describe the abundance of the relevant cell populations within post-sort fractions, providing details on the purity of the samples and how it was determined.*
Gating strategy	*Describe the gating strategy used for all relevant experiments, specifying the preliminary FSC/SSC gates of the starting cell population, indicating where boundaries between "positive" and "negative" staining cell populations are defined.*

☐ Tick this box to confirm that a figure exemplifying the gating strategy is provided in the Supplementary Information.

Magnetic resonance imaging

Experimental design

Design type	*Indicate task or resting state; event-related or block design.*
Design specifications	*Specify the number of blocks, trials or experimental units per session and/or subject, and specify the length of each trial or block (if trials are blocked) and interval between trials.*
Behavioral performance measures	*State number and/or type of variables recorded (e.g. correct button press, response time) and what statistics were used to establish that the subjects were performing the task as expected (e.g. mean, range, and/or standard deviation across subjects).*

Acquisition

Imaging type(s)	*Specify: functional, structural, diffusion, perfusion.*
Field strength	*Specify in Tesla*
Sequence & imaging parameters	*Specify the pulse sequence type (gradient echo, spin echo, etc.), imaging type (EPI, spiral, etc.), field of view, matrix size, slice thickness, orientation and TE/TR/flip angle.*
Area of acquisition	*State whether a whole brain scan was used OR define the area of acquisition, describing how the region was determined.*

Diffusion MRI ☐ Used ☐ Not used

Preprocessing

Preprocessing software	*Provide detail on software version and revision number and on specific parameters (model/functions, brain extraction, segmentation, smoothing kernel size, etc.).*
Normalization	*If data were normalized/standardized, describe the approach(es): specify linear or non-linear and define image types used for transformation OR indicate that data were not normalized and explain rationale for lack of normalization.*
Normalization template	*Describe the template used for normalization/transformation, specifying subject space or group standardized space (e.g. original Talairach, MNI305, ICBM152) OR indicate that the data were not normalized.*
Noise and artifact removal	*Describe your procedure(s) for artifact and structured noise removal, specifying motion parameters, tissue signals and physiological signals (heart rate, respiration).*
Volume censoring	*Define your software and/or method and criteria for volume censoring, and state the extent of such censoring.*

Statistical modeling & inference

Model type and settings	*Specify type (mass univariate, multivariate, RSA, predictive, etc.) and describe essential details of the model at the first and second levels (e.g. fixed, random or mixed effects; drift or auto-correlation).*
Effect(s) tested	*Define precise effect in terms of the task or stimulus conditions instead of psychological concepts and indicate whether ANOVA or factorial designs were used.*

Specify type of analysis: ☐ Whole brain ☐ ROI-based ☐ Both

Statistic type for inference (See Eklund et al. 2016)	*Specify voxel-wise or cluster-wise and report all relevant parameters for cluster-wise methods.*
Correction	*Describe the type of correction and how it is obtained for multiple comparisons (e.g. FWE, FDR, permutation or Monte Carlo).*

Models & analysis

n/a	Involved in the study
☐	☐ Functional and/or effective connectivity
☐	☐ Graph analysis
☐	☐ Multivariate modeling or predictive analysis

Functional and/or effective connectivity

Report the measures of dependence used and the model details (e.g. Pearson correlation, partial correlation, mutual information).

Graph analysis

Report the dependent variable and connectivity measure, specifying weighted graph or binarized graph, subject- or group-level, and the global and/or node summaries used (e.g. clustering coefficient, efficiency, etc.).

Multivariate modeling and predictive analysis

Specify independent variables, features extraction and dimension reduction, model, training and evaluation metrics.
